# Supplementary material for: Cell cycle-driven transcriptome maturation confers multilineage competence to cardiopharyngeal progenitors
Source: EMBO J. 2025 Nov 3;44(24):7649–76. doi: 10.1038/s44318-025-00613-y (PMC12705688; doi:10.1038/s44318-025-00613-y)
Supplement: Supplementary file 1 — Appendix [file 44318_2025_613_MOESM1_ESM.pdf]

Appendix Figures and Legends

Appendix Figure S1 - Figure 1-S1. Inhibition of mitotic entry in TVC lineage resulted in reduced Tbx1/10 expression in STVC. 2
Appendix Figure S2 - Figure 1-S2. Cdc25-overexpressing TVCs undergo abnormal cell division with the loss of asymmetry and loss of medio-lateral orientation. 3
Appendix Figure S3 - Figure 1-S3. Representative confocal images of Cdc25OE perturbation time series. 4
Appendix Figure S4 - Figure 1-S4. The accumulation of Tbx1/10 in the TVC descendants is affected by the mitotic perturbation of Cdc25, with niche-driven mechanisms involved. 5
Appendix Figure S5 - Figure 1-S5. Cell cycle progression dynamics of TVCs under perturbations and in controls. 6
Appendix Figure S6 - Figure 2-S1. Initial analysis of the scRNA-seq data 7
Appendix Figure S7 - Figure 2-S2. Supplemental RNA velocity analysis 8
Appendix Figure S8 - Figure 2-S3. Un-denoised trajectories of the B7.5 cardiopharyngeal lineage. 9
Appendix Figure S9 - Figure 3-S1. Supplemental 10
Appendix Figure S10 - Figure 4-S1. Propagation of time stamps to the whole embryo dataset through label transfer. 11
Appendix Figure S11 - Figure 4-S2. Expression of tissue-specific markers across the whole embryo scRNA-seq dataset. 13
Appendix Figure S12 - Figure 4-S3. Endomesodermal trajectories. 14
Appendix Figure S13 - Figure 6-S1. G2 progression permits accumulation of Depdc1b mRNAs in the TVCs. 15
Appendix Figure S14 to S20 - Figure 7-S1 to -S7 (below). Detailed expression profiles of candidate transcription factor coding genes. 15
Appendix Figure S14 16
Appendix Figure S15 17
Appendix Figure S16 20
Appendix Figure S17 22
Appendix Figure S18 24
Appendix Figure S19 26
Appendix Figure S20 28
Appendix Figure S21 - Figure 7-S8. Sample barcode recovery in the CRISPR x scRNA-seq experiment. 29
Appendix Figure S22 - Figure 7-S9. CRISPR/Cas9-mediated mutagenesis targeting Foxf, Gata4/5/6, and Foxtun4, followed by scRNA-seq of FACS-purified cardiopharyngeal lineage cellss. 30
Appendix Figure S23 - Figure 8-S1. Putative Transcription Factor Binding Motifs in the Depdc1b enhancer, which are conserved between Ciona robusta and Ciona savignyi, and in the Depdc1b proximal promoter region. 31

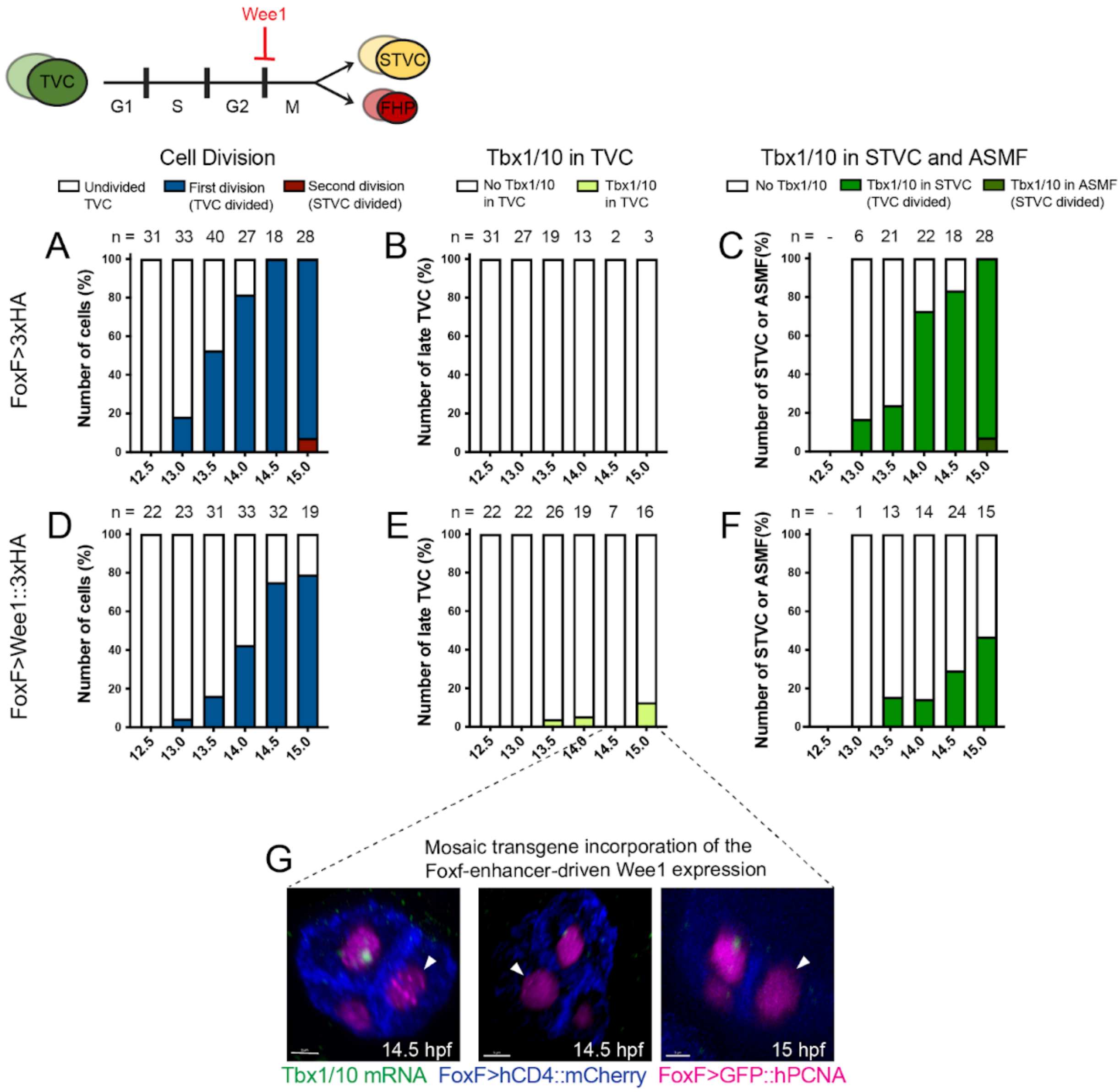

Appendix Figure S1 - Figure 1-S1. Inhibition of mitotic entry in TVC lineage resulted in reduced Tbx1/10 expression in STVC.

A-F. Cell division patterns (A and D) and *Tbx1/10* expression upon cell mitosis suppression perturbations (Wee1::3xHA) in the late TVCs (B and E) and STVCs (C and F). Note that the results of A, B, D, E used the same dataset as Figure 1F, 1H, 1F’, and 1H’.

G. Confocal images showing mosaic transgene inheritance of Wee1::3xHA under the Foxf enhancer in some samples. Magenta: nuclei (GFP::PCNA); Blue: cell membranes (hCD4::mCherry); White arrowhead: late TVC, Scale bar = 5 μm. No blinding was included in the analysis.

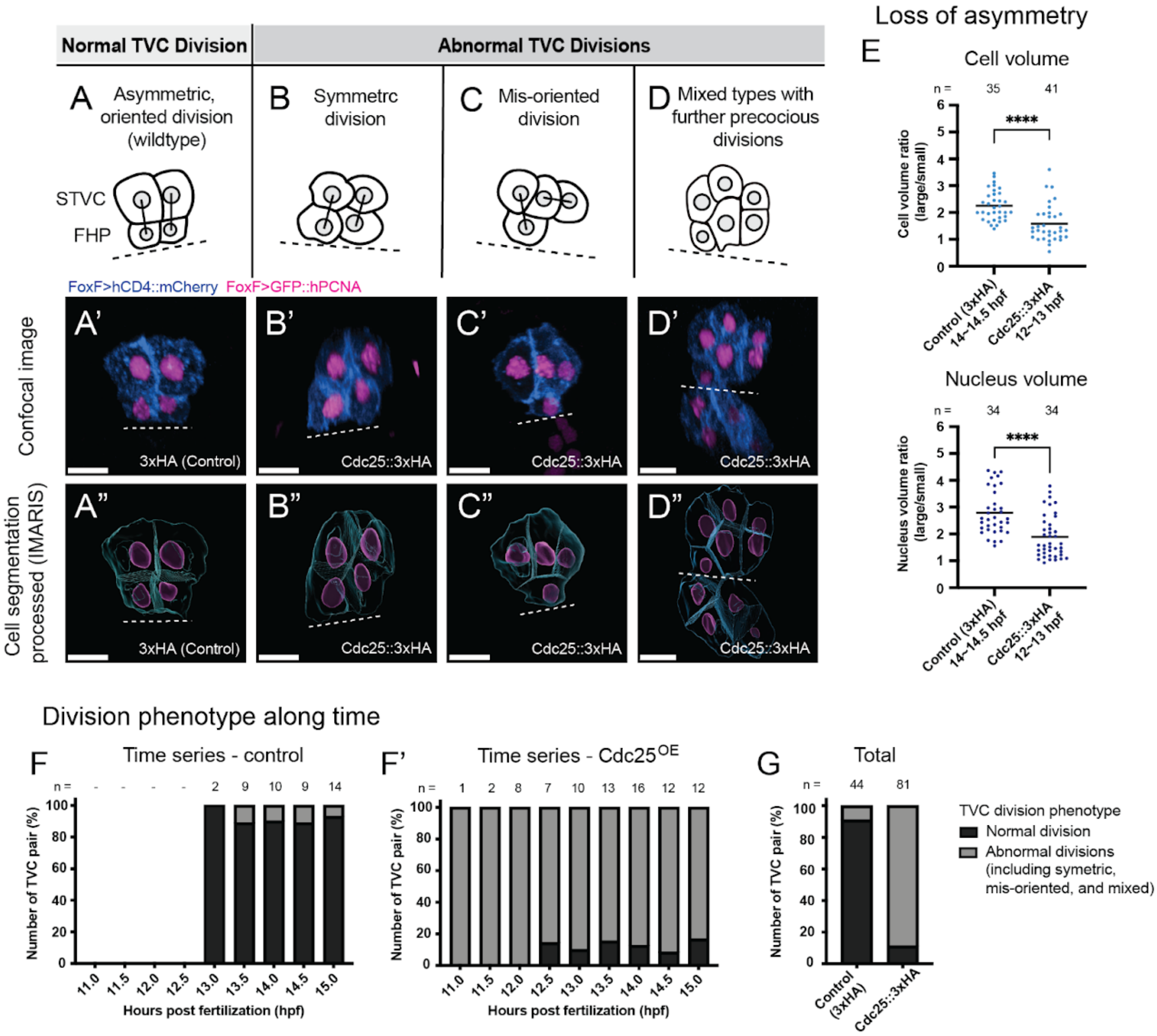

Appendix Figure S2 - Figure 1-S2. Cdc25-overexpressing TVCs undergo abnormal cell division with the loss of asymmetry and loss of medio-lateral orientation.

A-D. Schematic diagrams of four categories of normal and abnormal division phenotypes of TVC. Normal division (A, asymmetric oriented) produces a pair of lateral large STVCs and small medial FHPs. Abnormal divisions include symmetric (B), mis-oriented (C), and the mixed types (D). A'-D' and A"-D". Representative images of each division category under the control and mitosis perturbations (Cdc25) conditions. Magenta: nuclei (GFP::PCNA); Blue: cell membranes (hCD4::mCherry); Dashed line: embryo midline, Scale bar = 10mm.

E-F. Division phenotype of TVC throughout developmental time points in the control (E) and Cdc25-overexpressing (E') samples. With results from different time points combined, F shows the total effect of Cdc25 on mis-regulating mitosis. G. Quantification of the cell volume ratio (left) and the nuclear volume ratio (right) of TVC descendant cells in the control and Cdc25-overexpression conditions. **** P < 0.0001.

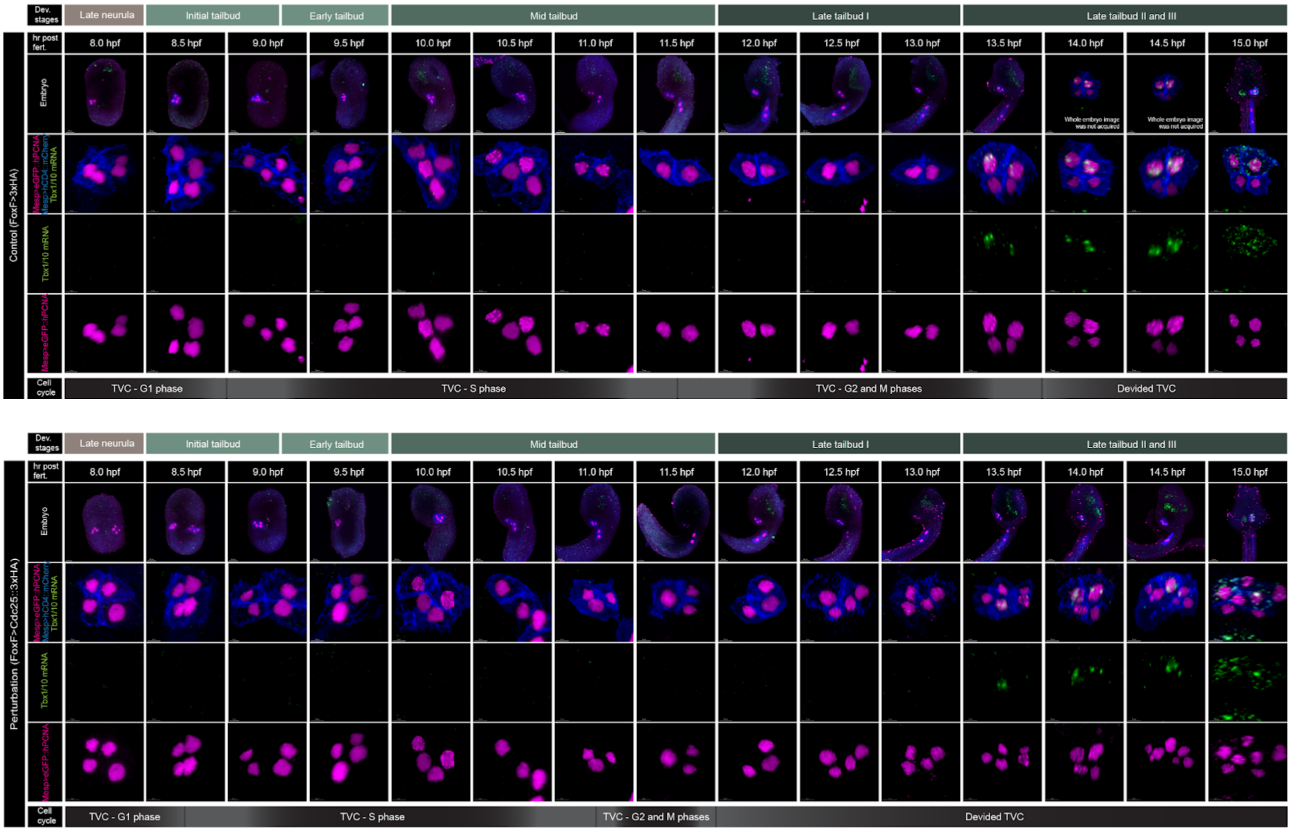

<u>Appendix Figure S3 - Figure 1-S3. Representative confocal images of Cdc25^{OE} perturbation time series.</u>

The division and Tbx1/10 expression phenotypes can be seen in the control (Top) and the Cdc25-overexpressing (Bottom) samples. Embryogenesis developmental stages and the cell cycle details of TVC are shown. Magenta: nuclei (GFP::PCNA); Blue: cell membranes (hCD4::mCherry); Scale bar = 20 μm for embryos, 5 μm for enlarged images.

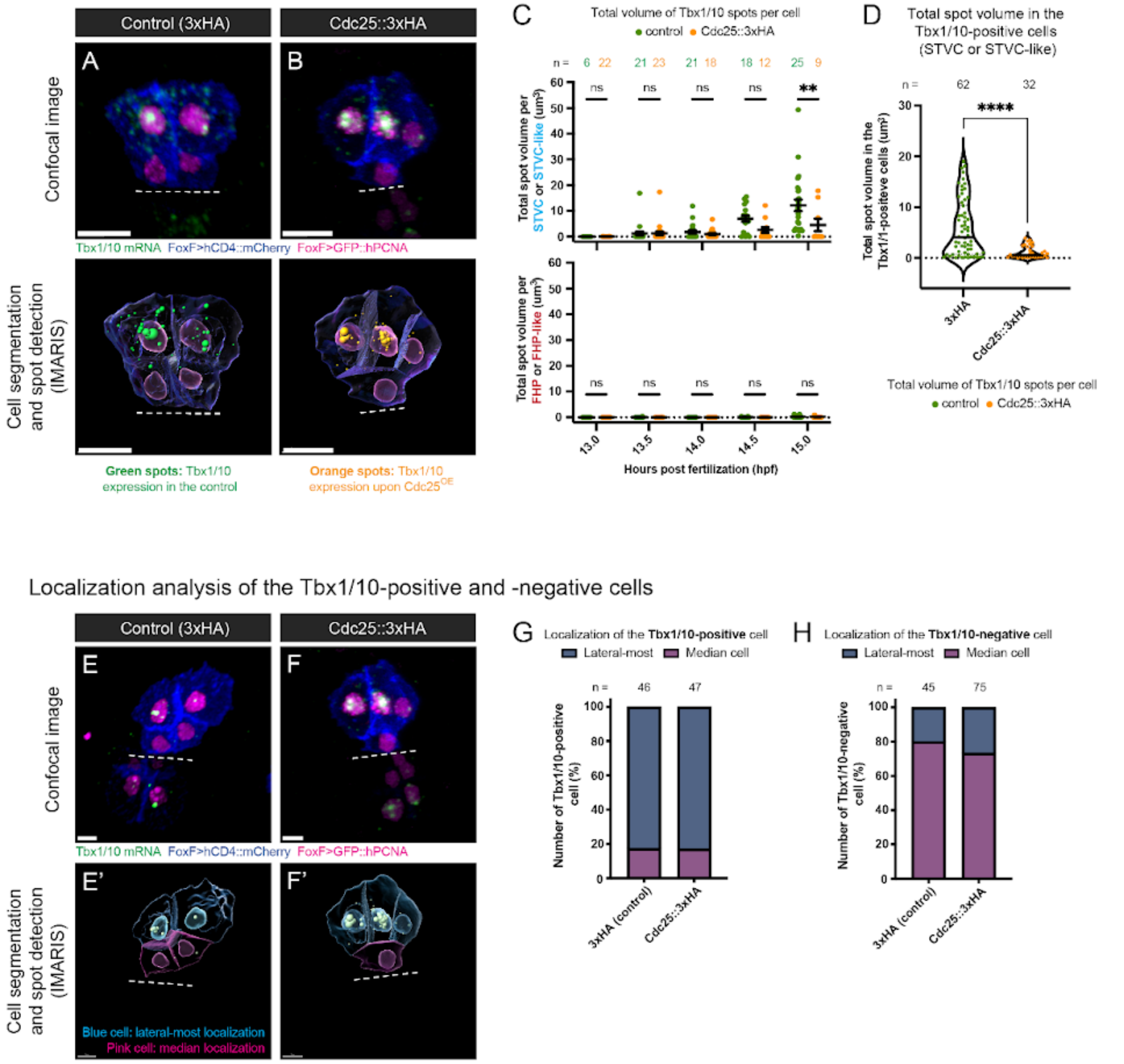

Appendix Figure S4 - Figure 1-S4. The accumulation of *Tbx1/10* in the TVC descendants is affected by the mitotic perturbation of Cdc25, with niche-driven mechanisms involved.

A-B. Representative confocal images of Tbx1/10 expressed in TVC descendants in the control and Cdc25OE samples. Magenta: nuclei (GFP::PCNA); Blue: cell membranes (hCD4::mCherry); Dashed line = embryo midline; Scale bar = 10mm. Note that panel B uses the same image data as Figure 2S panel C for representation.

A'-B'. Processed images with cell segmented and Tbx1/10 FISH spot signal detected in STVC or STVC-like cells. Green spot: FISH signal detected in the control; Orange spot: FISH signal detected in the Cdc25OE cells.

C. Developmental quantification of total volume of spots detected in individual STVC(-like) cells (top) and individual FHP(-like) cells (bottom) under the control and Cdc25OE conditions. ns, not significant; ** $P < 0.01$.

D. Total spot volume in Tbx1/10-expressing cells under the control and Cdc25OE conditions. **** $P < 0.0001$.

E-F and E'-F'. Representative confocal images of TVC descendants in the control and Cdc25OE samples. Images are processed by cell segmentation, FISH spot detection, and labeled by colors

based on cell localization. Processed blue cell: lateral-most localization; Processed magenta cell: median localization. Scale bar = 5mm.
G-H. Localization analysis of Tbx1/10-expressing (G) and Tbx1/10-negative (H) cells under the control conditions and Cdc25 perturbations.

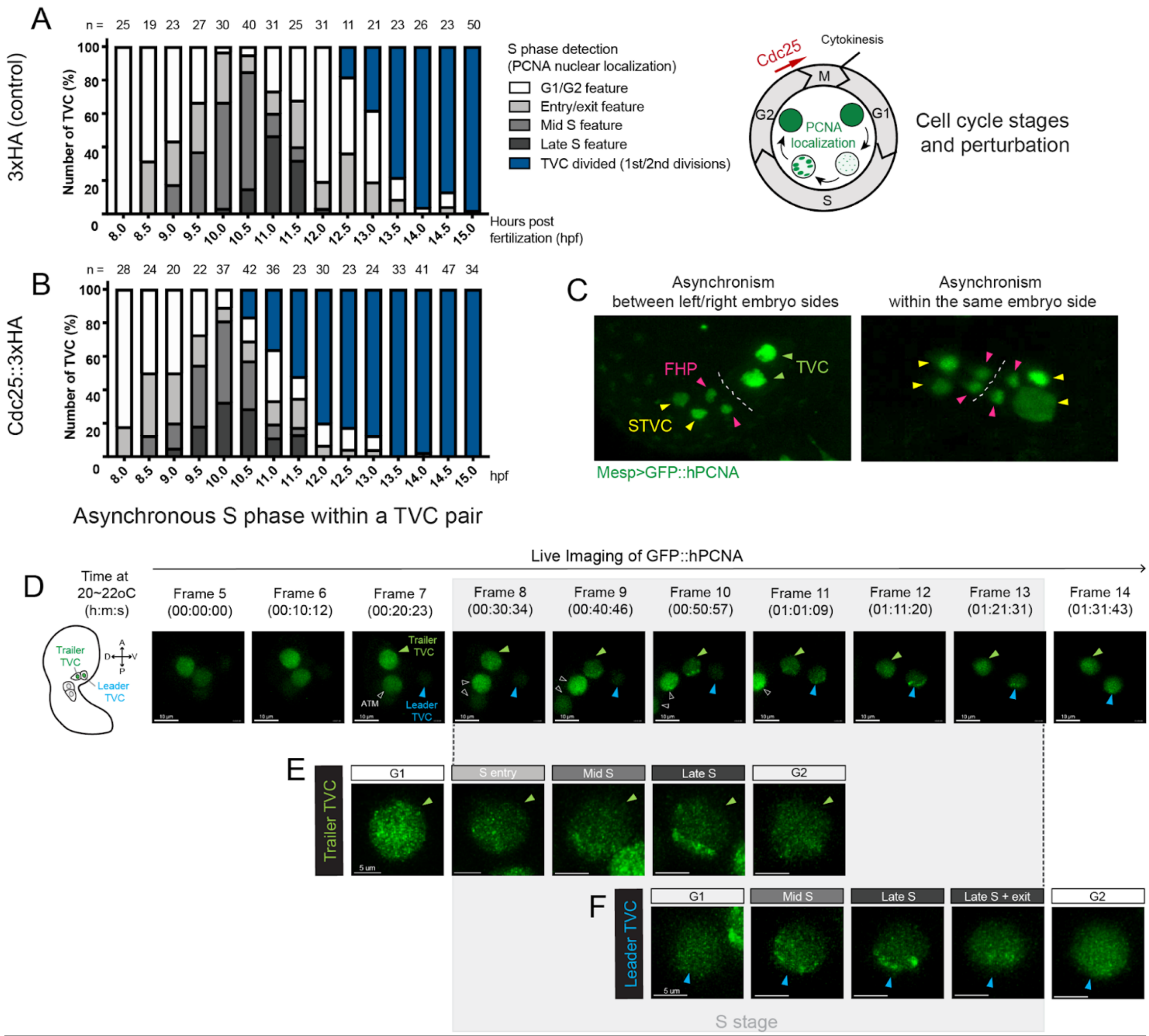

Appendix Figure S5 - Figure 1-S5. Cell cycle progression dynamics of TVCs under perturbations and in controls.

A. Schematic of cell cycle stages, genetic perturbation (Foxf>Cdc25) of G2/M transition, and variability of PCNA puncta size in the TVC nuclei associated with cell cycle progression.
B-C. Developmental distribution of S phase (four PCNA localization patterns) and cell division of TVC in the control and Cdc25OE samples. Cdc25-overexpressing TVCs are permitted to divide right after passing through S/G2 transition (C).
D. Confocal images showing asynchronous division of TVC-lineage cells. (Left) Asynchronous TVC division between left/right body sides. (Right) Asynchronous division within the same body side. Green: nuclei (GFP::PCNA); Green arrowhead: TVC; Yellow arrowhead: STVC; Magenta arrowhead: FHP.
E. Live imaging of PCNA puncta dynamics in the leader and trailer TVCs along TVC cell cycle progression revealed asynchronous S phase within collective migrating TVC pair. Green: nuclei (GFP::PCNA); Blue arrowhead: leader TVC; Green arrowhead: trailer TVC; Scale bar = 10mm.

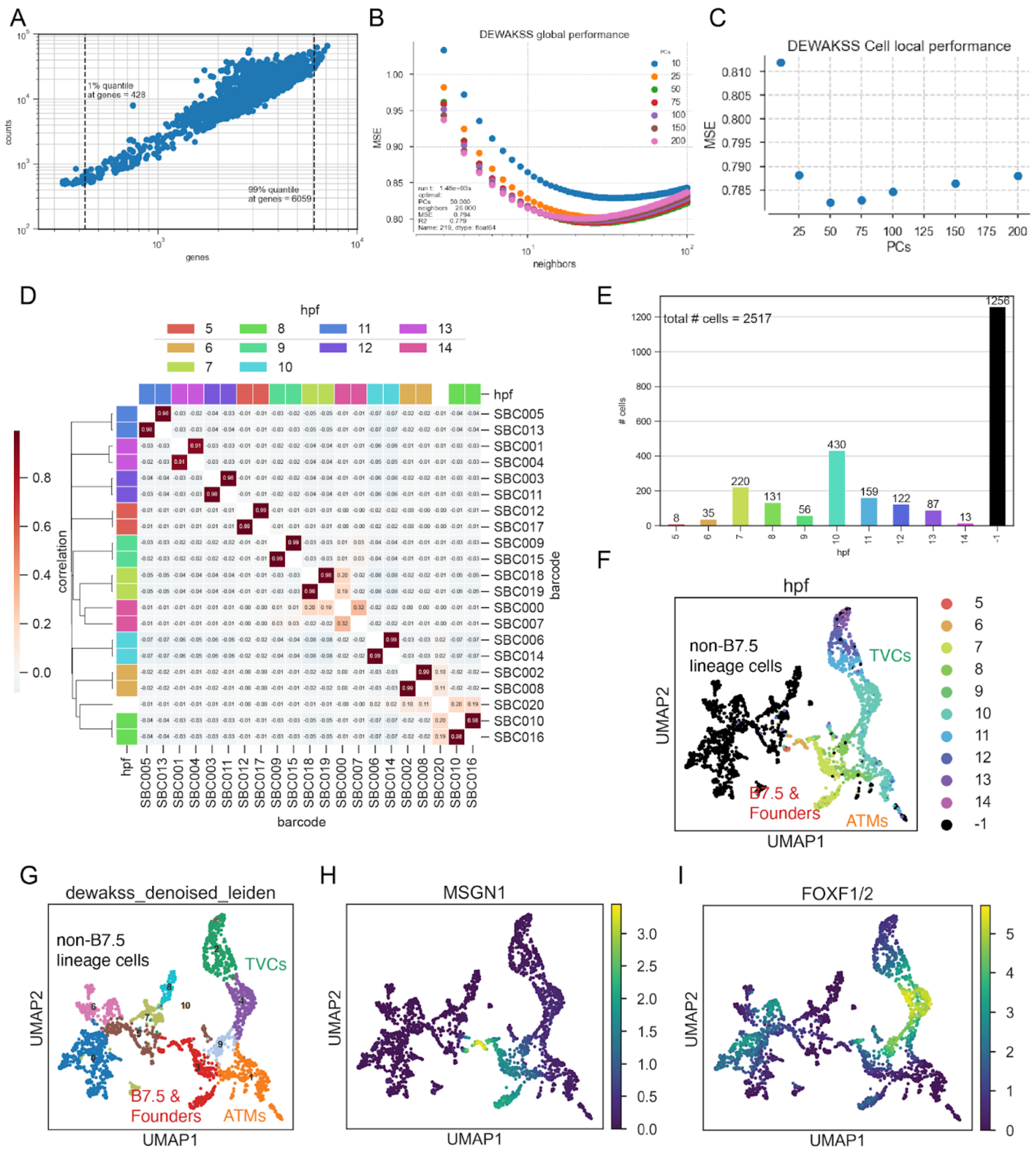

Appendix Figure S6 - Figure 2-S1. Initial analysis of the scRNA-seq data

A. Number of genes detected vs. the number of unique molecular identifiers (UMIs) per cell.

B. Denoising with DEWAKSS hyperparameter grid search. Each color represents the number of PCs used. X-axis is the number of neighbors, performance of DEWAKSS in mean square error (MSE) on y-axis.

C. Performance when optimal number of PCs and neighbors per cell are used.

D. Correlations of double barcode system to assign cells to real developmental time. Pearson correlation coefficient is shown.

E. Number of cells detected per real-time barcode pair. -1 are cells that did not pass barcode annotation threshold selection (see Methods).

F. UMAP showing inferred real-time barcodes and lineage annotations.

G. Leiden clustering given derived kNN graph from DEWAKSS.

H,I. TVC-lineage marker genes for expected trajectory root (MSGN1, expressed in founder cells) and branch (Foxf, expressed in cardiopharyngeal lineage).

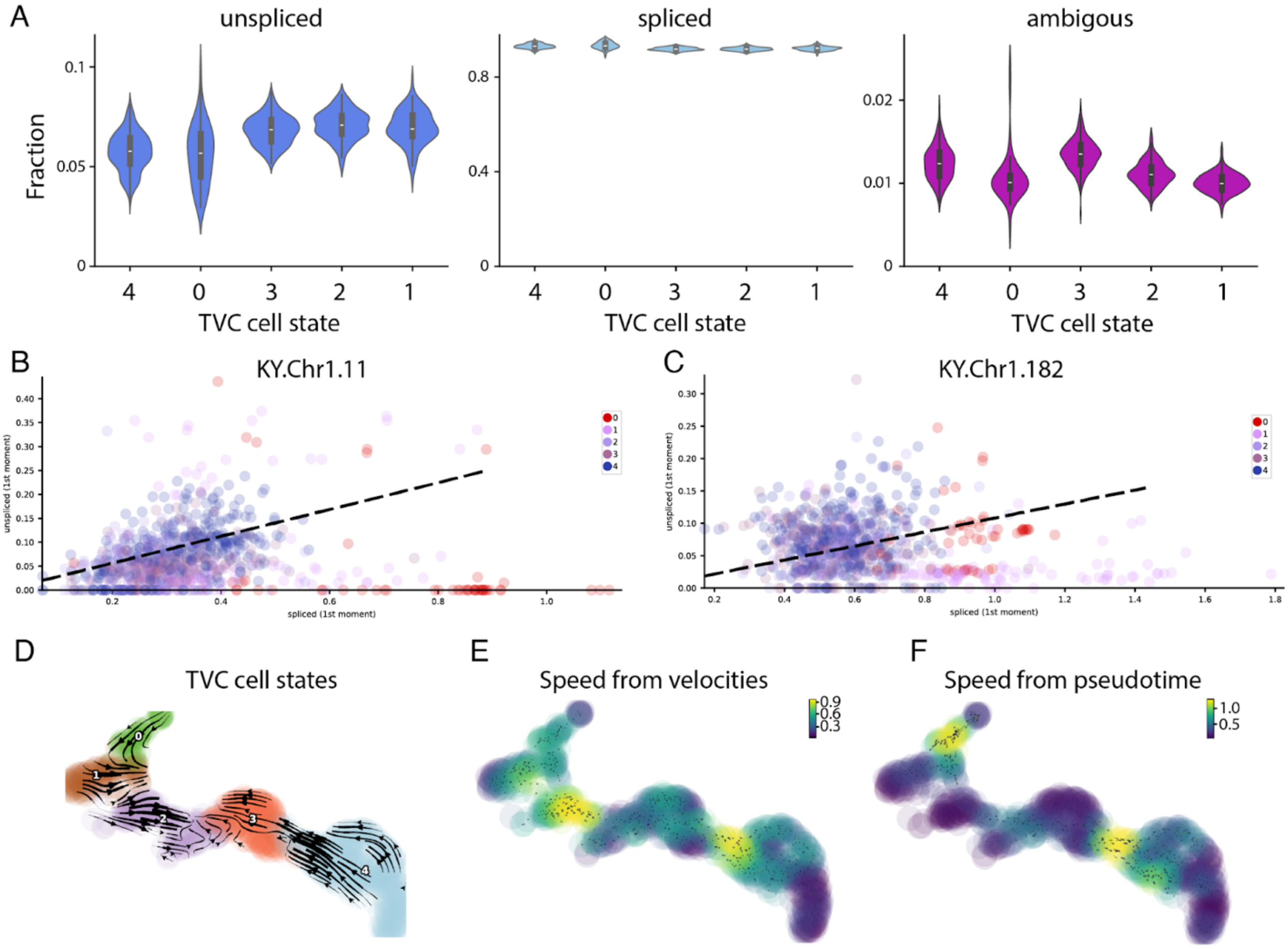

<u>**Appendix Figure S7 - Figure 2-S2. Supplemental RNA velocity analysis**</u>

A. Violin plots of the fraction of spliced, unspliced and ambiguous reads grouped by each cell state of the TVC trajectory.

B,C. Phase plot of the spliced and unspliced first order moments of the RNA velocities of KY.Chr1.11 and KY.Chr1.182, respectively. Both genes were selected by dynamo to calculate the dynamics. Dots are colored by cell state of the TVC trajectory.

D. UMAP representation of the TVC only trajectory colored by cell state and overlayed by RNA velocities calculated using dynamos deterministic mode and spliced and unspliced reads. Represented by streamlines.

E. UMAP representation of the TVC only trajectory colored by speed (i.e. the length) of the velocity vector of each cell. Velocity for this plot was calculated using dynamos deterministic mode and spliced and unspliced reads.

F. UMAP representation of the TVC only trajectory colored by speed (i.e. the length) of the velocity vector of each cell. Velocity for this plot was calculated from denoised data and diffusion pseudotime.

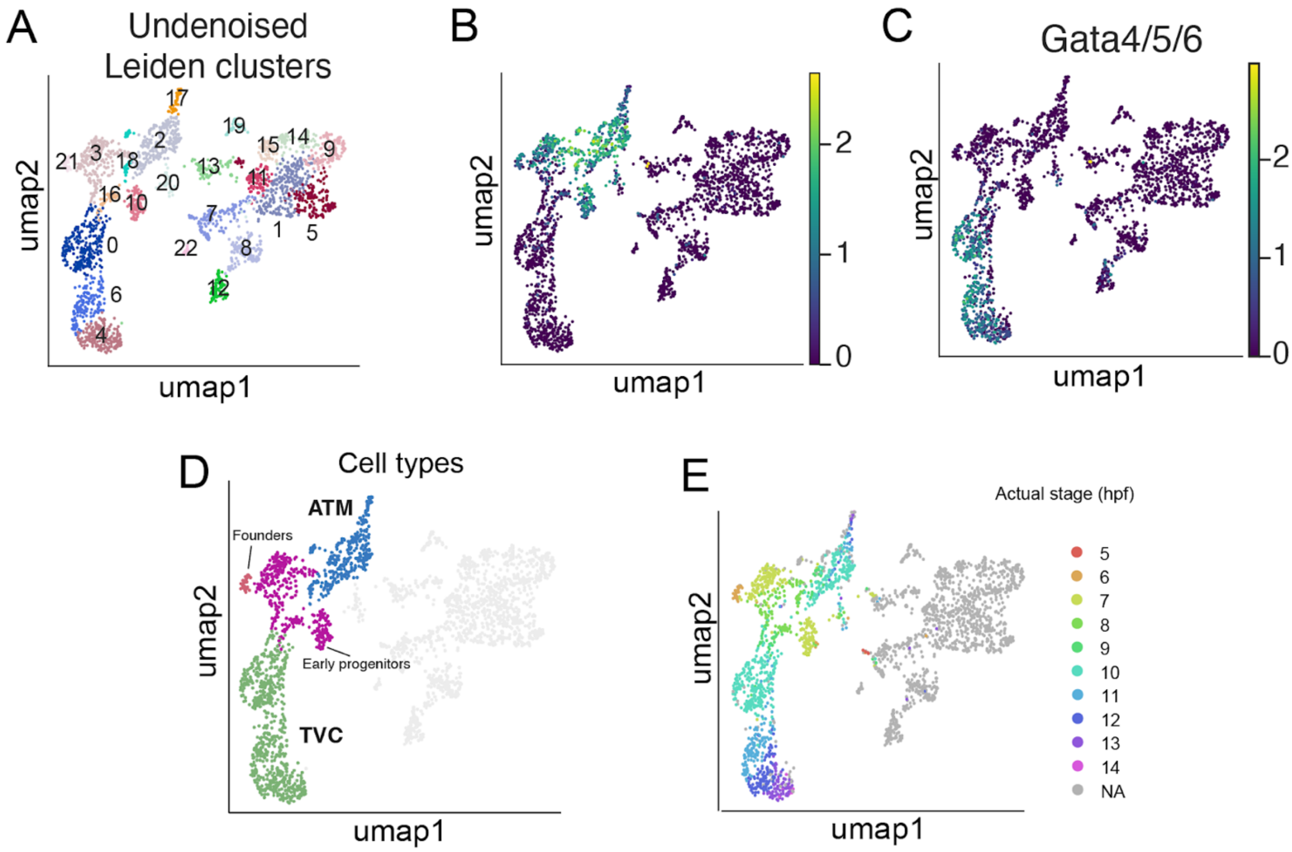

<u>Appendix Figure S8 - Figure 2-S3. Un-denoised trajectories of the B7.5 cardiopharyngeal lineage.</u>

A. Un-denoised Leiden clustering of anterior tail muscles and cardiopharyngeal trajectories. B-C. Trajectory-specific expression of founder and ATM-specific Mrf/Myod/Myf5 transcripts and TVC-specific Gata4/5/6 transcripts

D. Assignment of cell type to trajectories based on lineage-specific gene expression.

E. Reconstruction of cell trajectories using recovered real-time barcodes.

In both D and E grayed out cells represent unassigned cell contamination collected during the linage enrichment stage. These cells are removed from final analysis presented in the main figures.

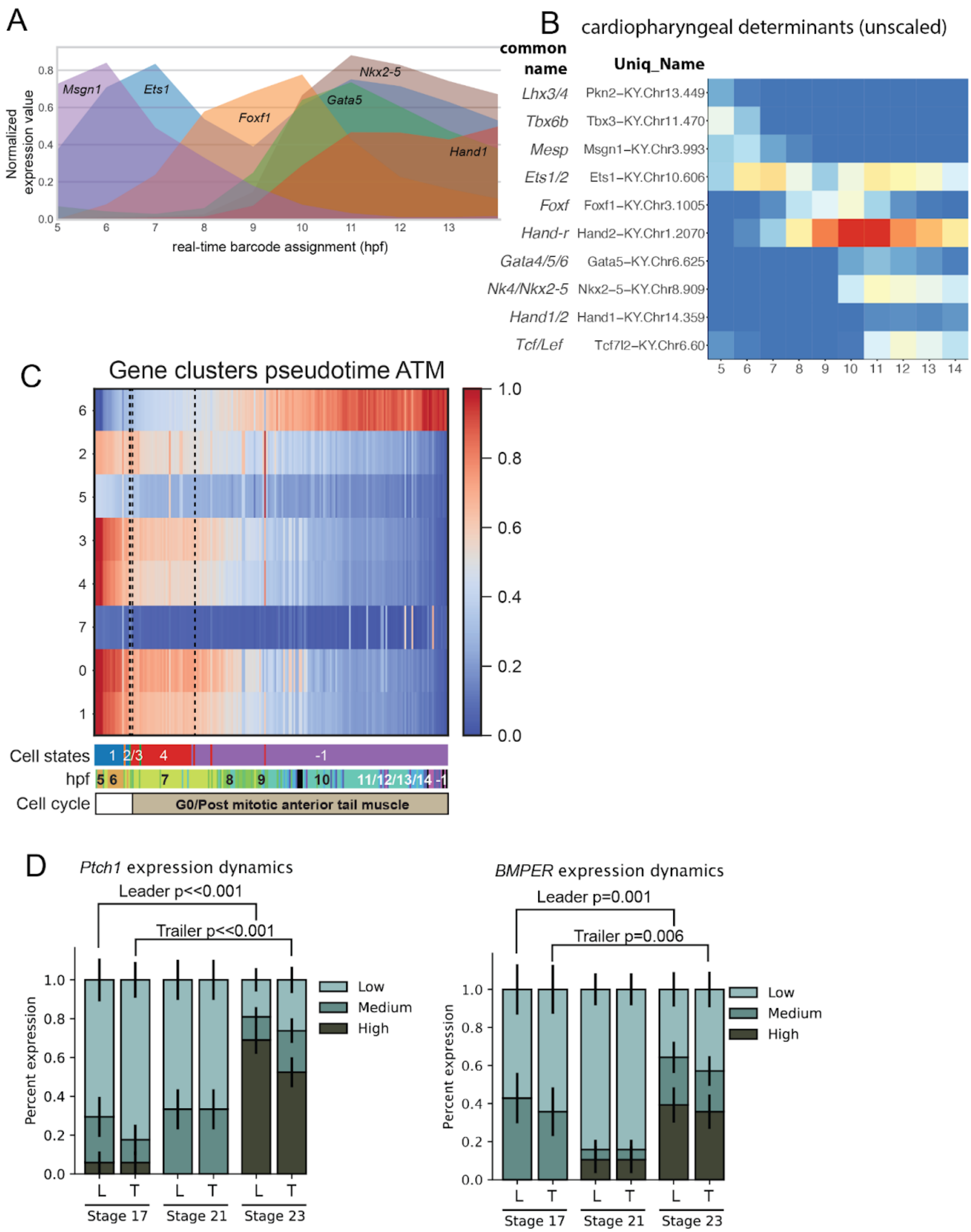

<u>Appendix Figure S9 - Figure 3-S1. Supplemental</u>

A. Normalized expression values of core cardiopharyngeal precursor genes (Mesp1, Ets1,Foxf1, Gata5, Nkx2-5, Hand1) as a function of real-time barcodes.

B. Unscaled expression of known cardiopharyngeal determination across time points.

C. Cell states and gene expression dynamics of post-mitotic Anterior Tail Muscles (ATMs).

D. Dynamics of Ptch1 and BMPER expression based on FISH analysis (Figure 2L). Embryos from indicated stages are scored based on qualitative expression level (low, medium, high). Standard error of proportion is shown. P-values were determined using the Pearson's c2 test.

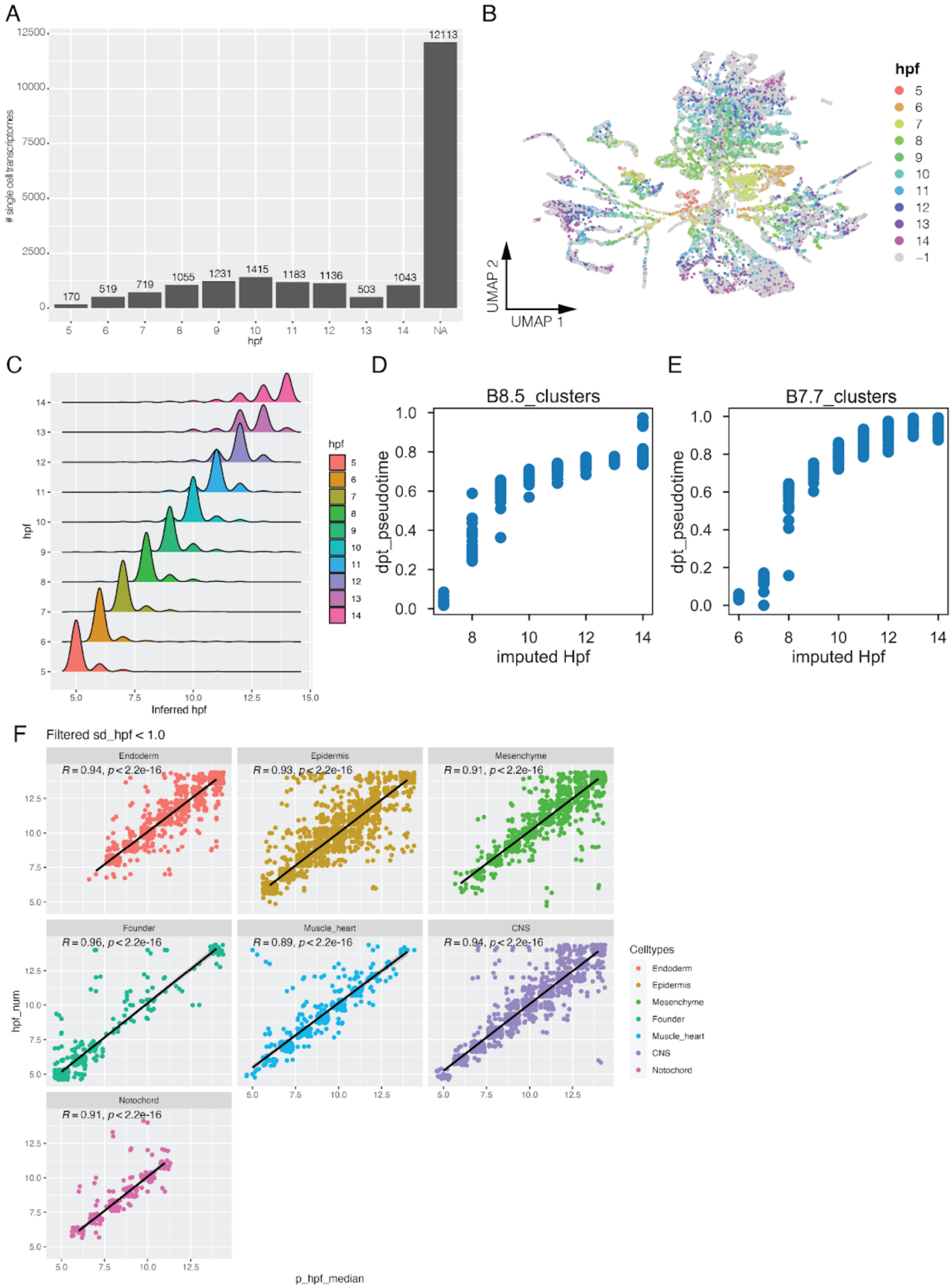

<u>**Appendix Figure S10 - Figure 4-S1. Propagation of time stamps to the whole embryo dataset through label transfer.**</u>

A. Frequency distribution of time specific barcodes across the developmental times profiled for the whole embryo dataset. Most cells did not express a time stamp.

B. UMAP representation of the whole embryo scRNA-seq dataset labeled with barcodes-derived time stamps only, also shown in Figure3-B.
C. Ridge plot showing the distribution of predicted developmental time through label transfer across all cells with a valid barcode-derived time stamp and binned by barcode-derived time stamp on the y axis.
D, E. Dot plot showing the correlation of inferred developmental time with diffusion pseudotime (dpt) for the cells of the mesenchymal B8.5 lineage (D) and the mesenchymal B7.7 lineage (E).
F. Correlation of barcode-derived developmental time with developmental time inferred through label transfer, shown for cells of each main tissue independently, as indicated in the panel.

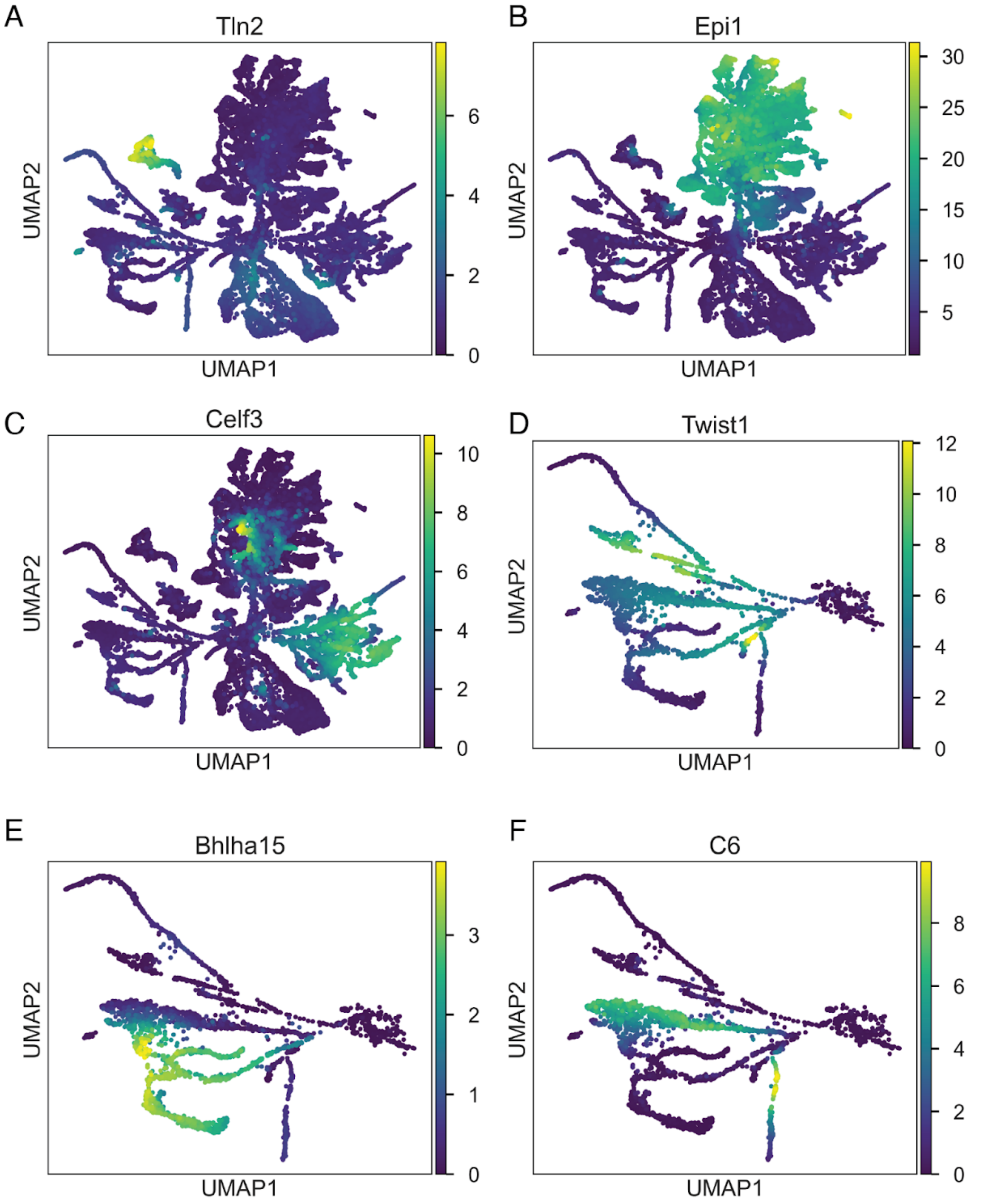

Appendix Figure S11 - Figure 4-S2. Expression of tissue-specific markers across the whole embryo scRNA-seq dataset.

A-C. Expression of a notochord specific marker Talin (A, KY.Chr3.779), an epidermis specific marker Epi1 (B, KY.Chr1.2380"), a CNS specific marker Celf3 (C, KY.Chr6.59) are shown on the UMAP representation of the whole embryo dataset.

D-F. Expression of the A7.6 specific marker Twist1 (D,, KY.Chr5.357), the B7.7 specific marker Bhlha15 (E, KY.Chr3.1309), and the B8.5 specific marker C6 (F, KY.Chr14.191), are shown on the UMAP representation of mesenchymal subset.

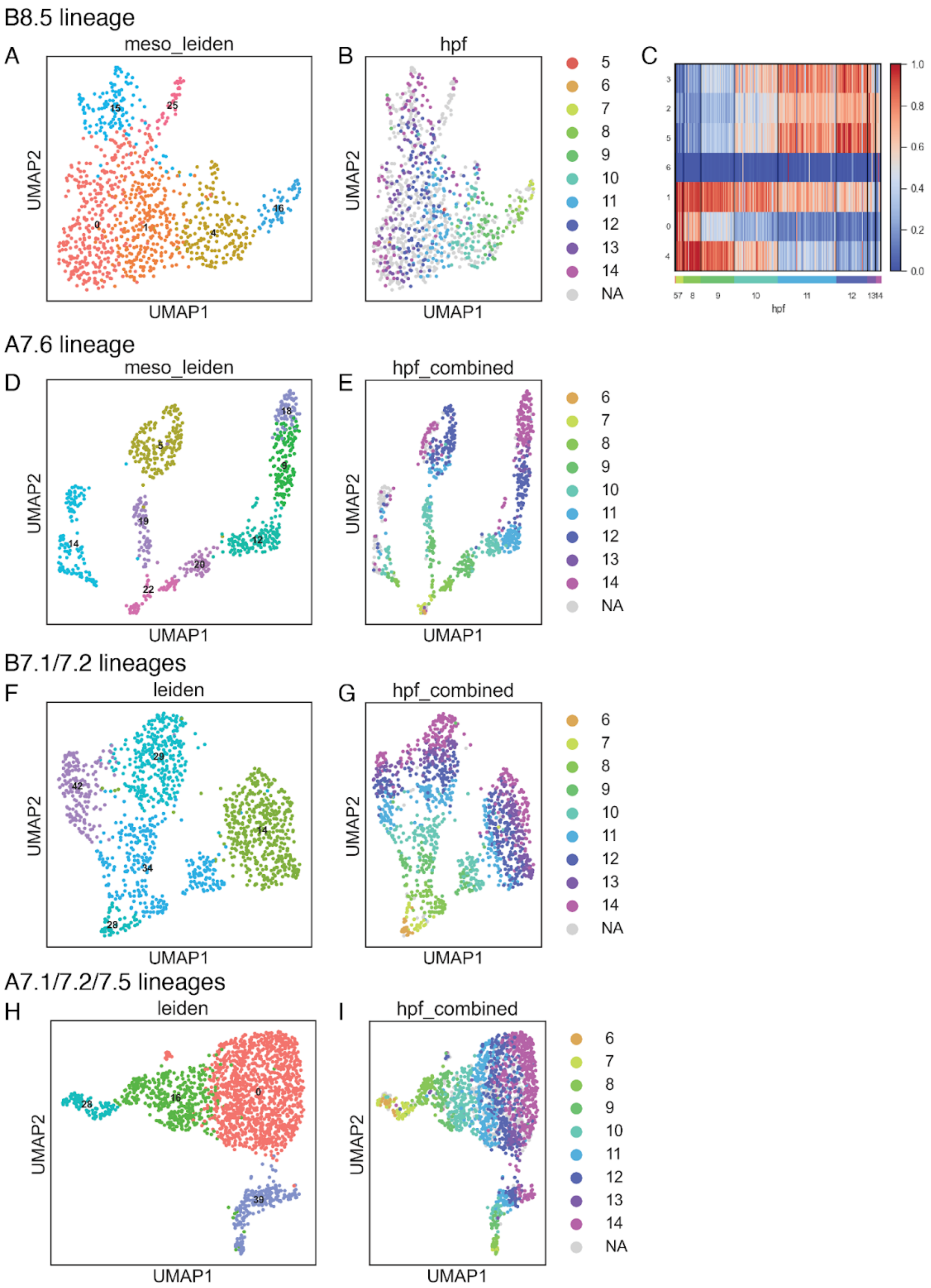

Appendix Figure S12 - Figure 4-S3. Endomesodermal trajectories.

A,B. Differentiation trajectory of the B8.5 mesenchymal lineage colored by inferred developmental time (A) and denoised Leiden clustering (B). Note that cluster 16 corresponds to an immature state and cluster 1 to a mature transcriptomic state.
C. Differential expression of genes during differentiation of the B8.5 mesenchymal lineage. Gene clusters 4 and 1 correspond to the immature and mature states, respectively.
D,E. Differentiation trajectory of the A7.6 mesenchymal lineage colored by inferred developmental time (D) and denoised Leiden clustering (E). Due to the rapid specification of these lineages early in development, no immature and mature cell state could be identified at the resolution of the present dataset for the A7.6 lineage.
F,G. Differentiation trajectory of the B7.1 and B7.2 endodermal lineages colored by inferred developmental time (F) and denoised Leiden clustering (G).
H,I. Differentiation trajectory of the A7.1, A7.2 and A7.5 endodermal lineage colored by inferred developmental time (H) and denoised Leiden clustering (I). Note that due to the unclear clonal relationship of the trajectories in the endoderm, we did not attempt to identify compatible mature and immature states.

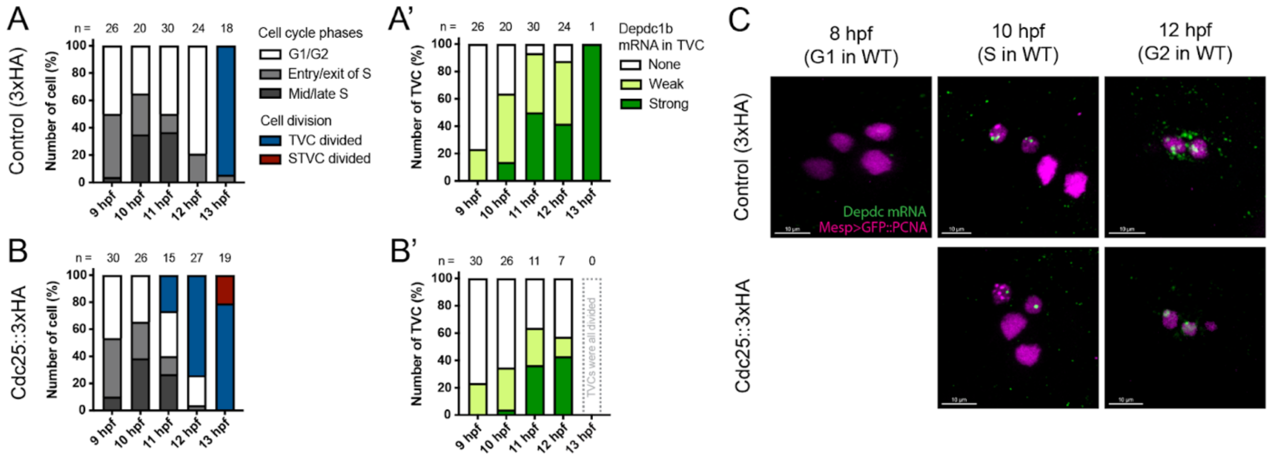

<u>**Appendix Figure S13 - Figure 6-S1. G2 progression permits accumulation of *Depdc1b* mRNAs in the TVCs.**</u>
A-B. Developmental distribution of S phase and cell division of TVC under the control and Cdc25$_{OE}$ conditions.
A'-B'. The corresponding *Depdc1b* expression phenotype along these developmental time points. Perforated bar in B' indicates no cells can be analyzed.
C. Representative images of Depdc1b expression at 8, 10, and 12 hpf in the control and the Cdc25-overexpressing cells. Magenta: nuclei (GFP::PCNA); Green: *Depdc1b* mRNA. Scale bar = 10 μm.

Appendix Figure S14 to S20 - Figure 7-S1 to -S7 (below). Detailed expression profiles of candidate transcription factor coding genes.
Candidate transcription factor coding genes are separated by clusters (S14 to S20 for clusters 0 to 6, respectively), and subclustered using scaled (top row) or unscaled (left column) data using the Heatmap function on the ShinyApp. Genes tend to subcluster primarily according to temporal profiles using scaled data, and according to expression levels using unscaled data.

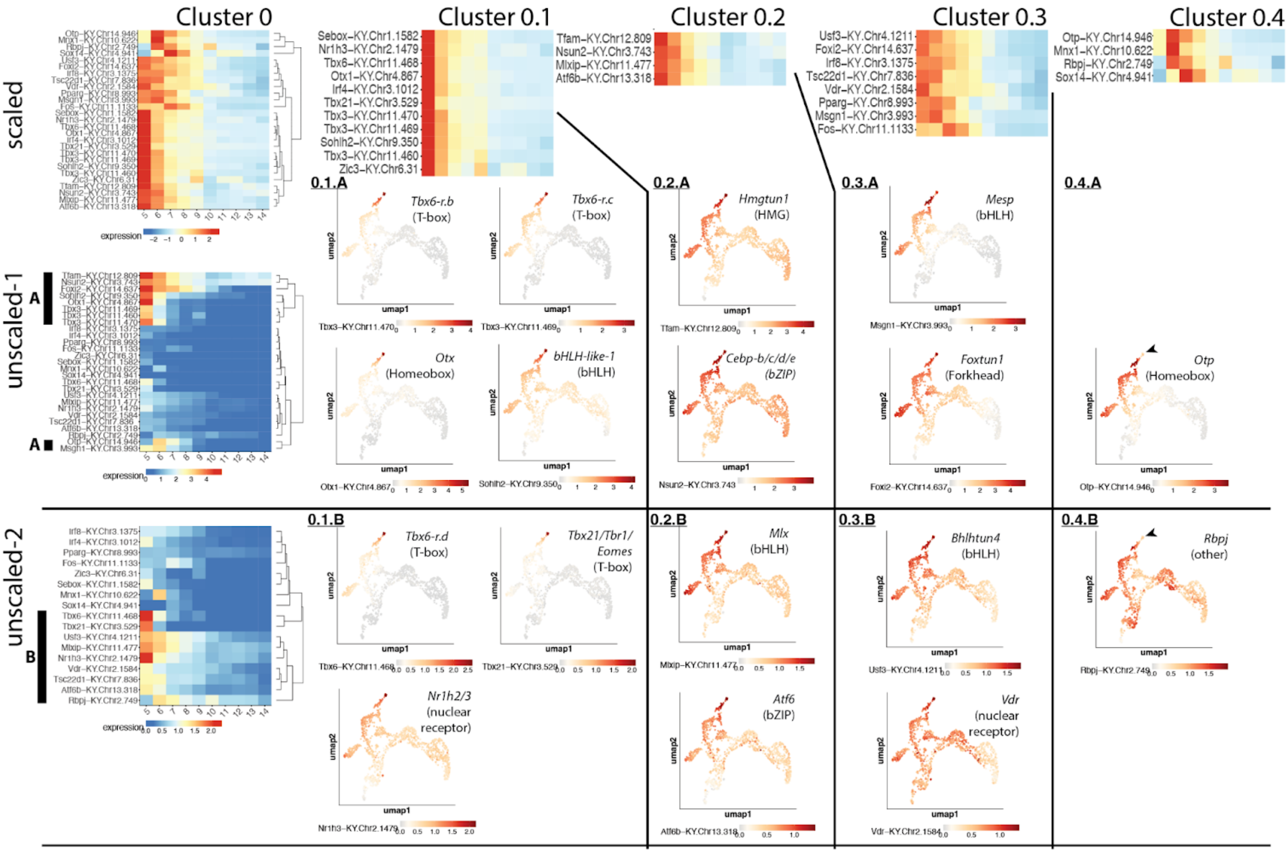

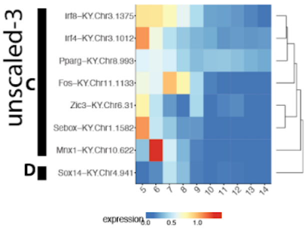

Cluster0	0.1	0.2	0.3	0.4
**A**				
	bHLH-like1	*Cebpb/c/d/e*	*Foxtun1*	*Otp*
	Otx	*Hmgtun1*	*Mesp*	
	Tbx6-r.a			
	Tbx6-r.b			
	Tbx6-r.c			
**B**				
	Nr1h2/3	*Atf6*	*Bhlhtun4/Usf3*	*Rbpj*
	Tbx21/Tbr1/Eomes	*Mlx*	*Tsc22*	
	Tbx6-r.d		*Vdr.a*	
**C**				
	IRF-like-5		*Fos*	*Mnx*
	Prdtun2		*IRF-like-7*	
	Zic-r.g/ZicL		*Ppar*	
**D**				
				Sox30

Appendix Figure S14

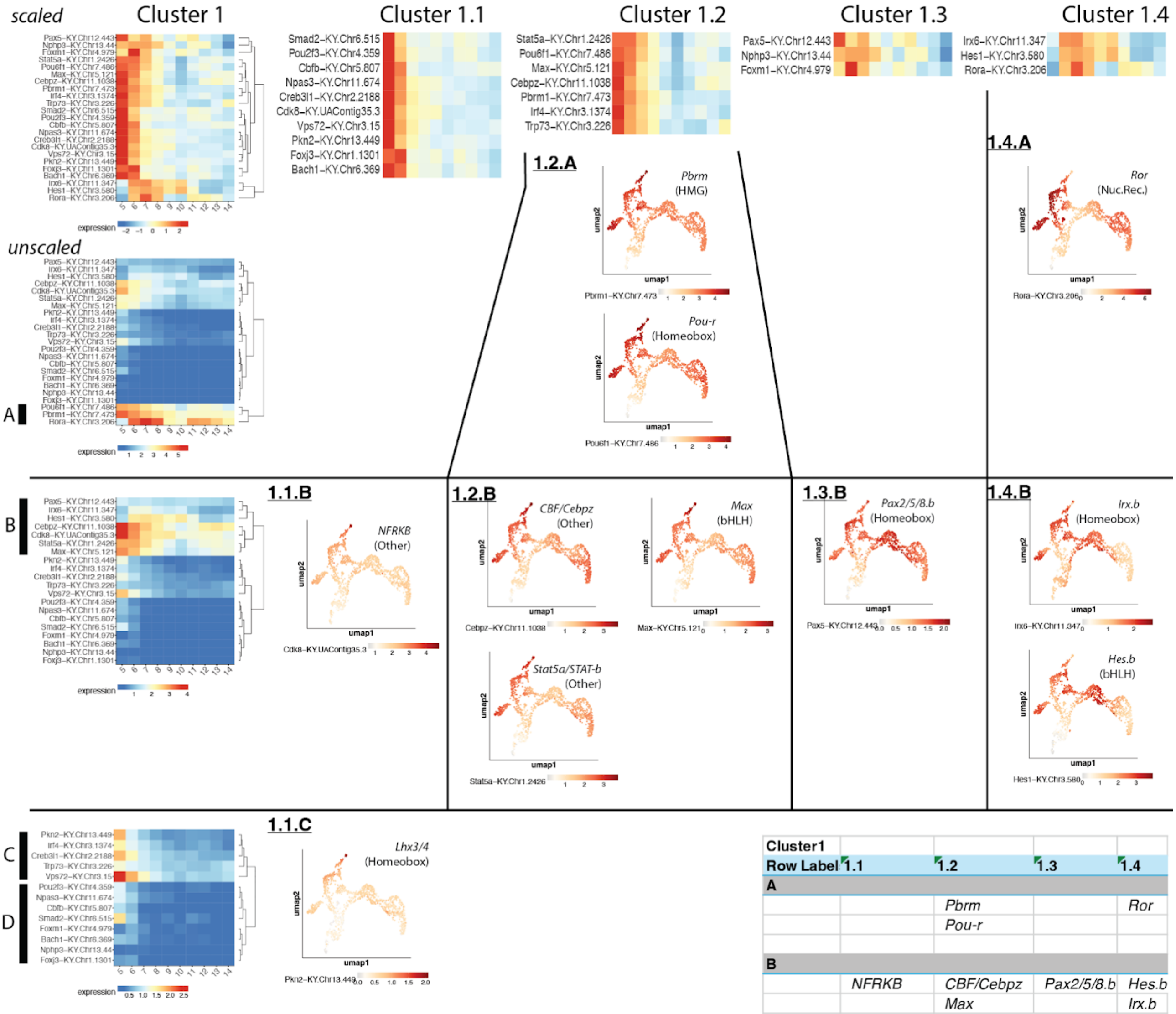

Cluster1				
Row Label	1.1	1.2	1.3	1.4
A				
		Pbrm		*Ror*
		Pou-r		
B				
	NFRKB	*CBF/Cebpz*	*Pax2/5/8.b*	*Hes.b*
		Max		*Irx.b*
		Stat5a/STAT-b		
C				
	Creb3l1	*IRF-like-8*		
	Lhx3/4	*Tp53/73.b*		
	TCFL1/Vps72			
	Zhx1-r			
D				
	Bach		*Bziptun1*	
	Cbfb		*Foxm*	
	Foxj2/3			
	Pou2F			
	Smad2/3.a			
	Trh			

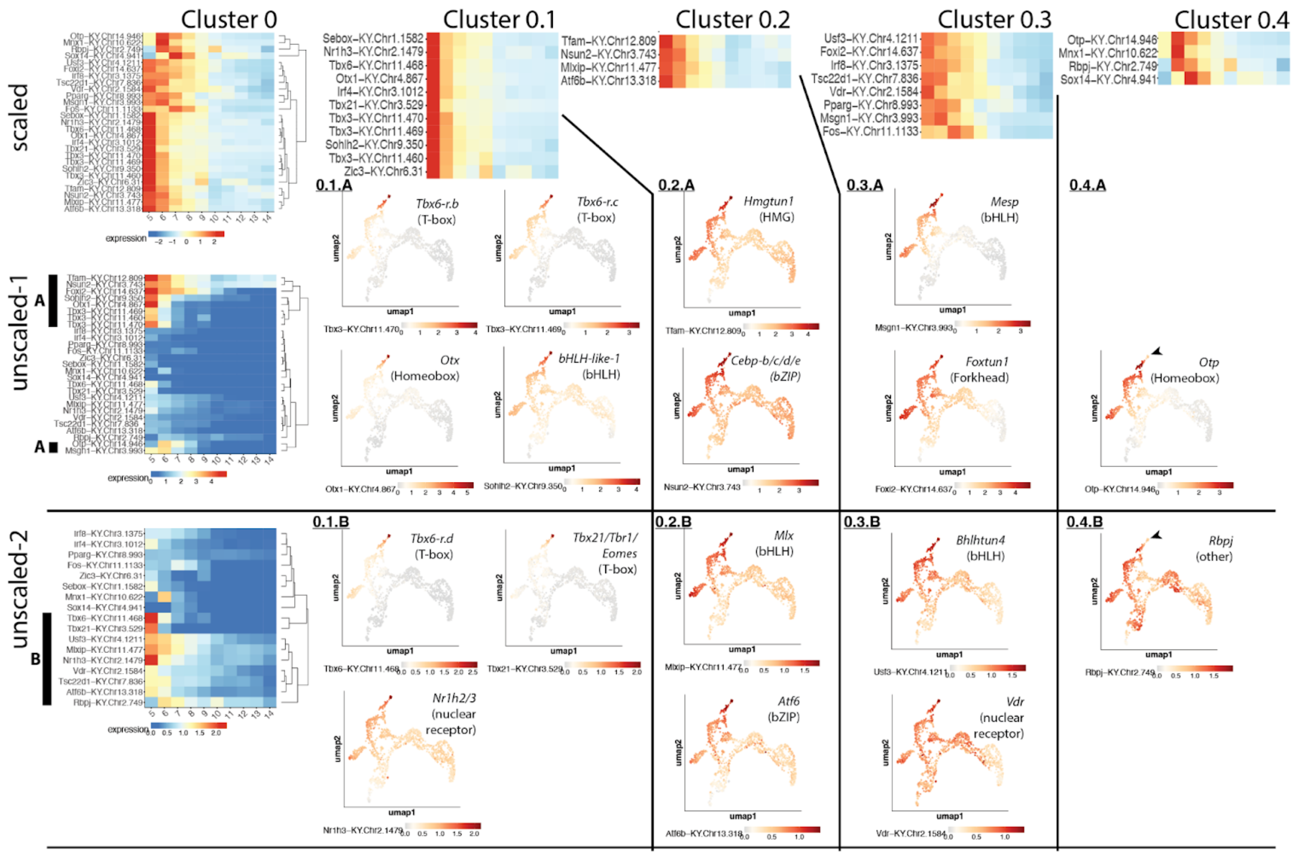

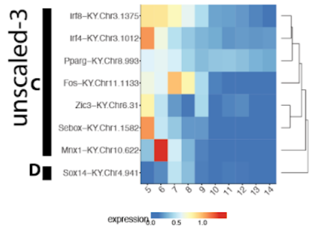

Cluster0				
	0.1	0.2	0.3	0.4
A				
	bHLH-like1	Cebpb/c/d/e	Foxtun1	Otp
	Otx	Hmgtun1	Mesp	
	Tbx6-r.a			
	Tbx6-r.b			
	Tbx6-r.c			
B				
	Nr1h2/3	Atf6	Bhlhtun4/Usf3	Rbpj
	Tbx21/Tbr1/Eomes	Mlx	Tsc22	
	Tbx6-r.d		Vdr.a	
C				
	IRF-like-5		Fos	Mnx
	Prdtun2		IRF-like-7	
	Zic-r.g/ZicL		Ppar	
D				
				Sox30

Appendix Figure S15

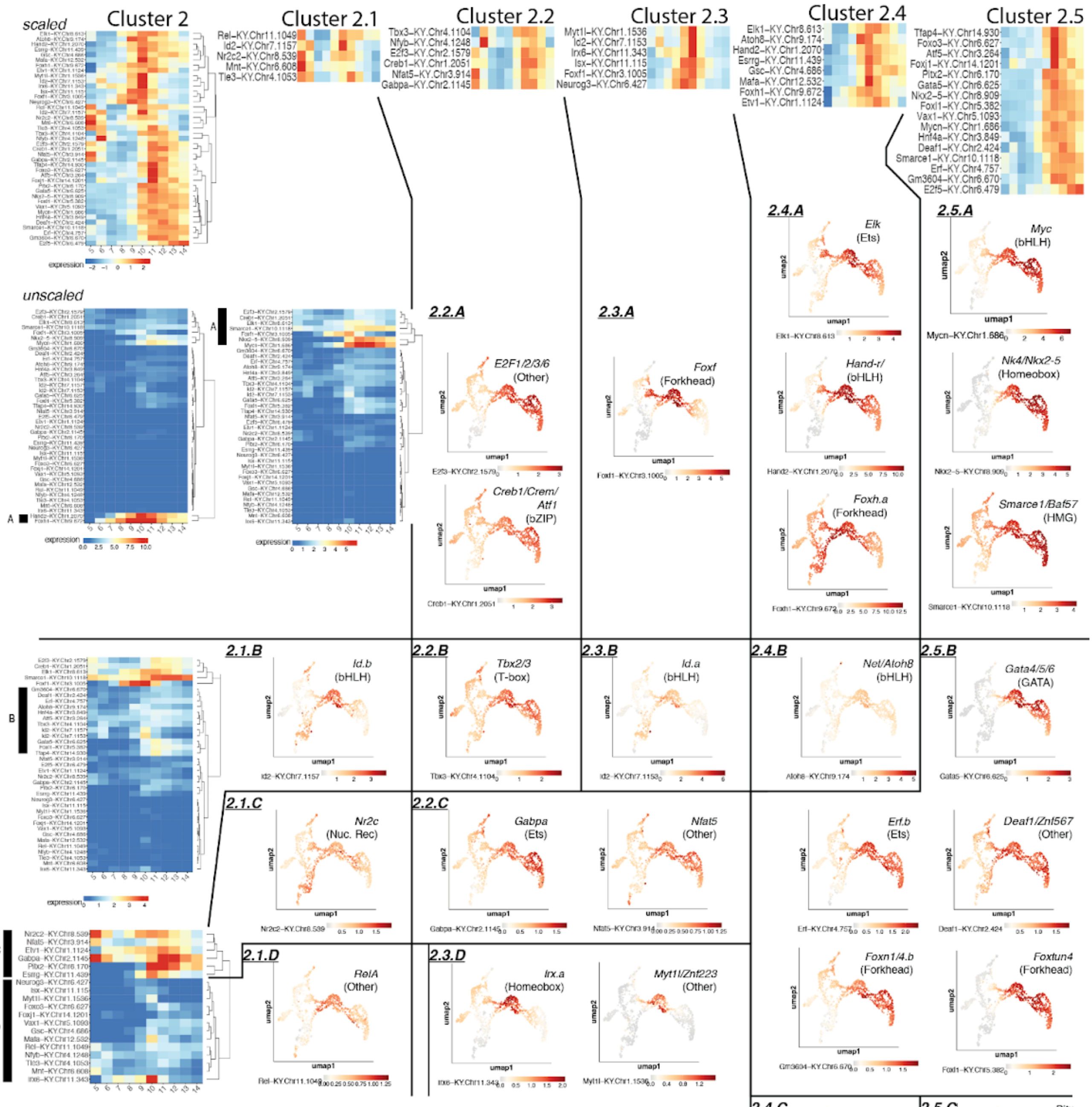

Cluster 2					
Row Labels	2.1	2.2	2.3	2.4	2.5
A					
		Creb1/Crem/Atf1	Foxf	Elk	Myc
		E2F1/2/3/6		Foxh.a	Nk4/Nkx2-5
				Hand-r/Notric	Smarce1/Baf57
B					
	Id.b	Tbx2/3	Id.a	Net/Atoh8	Atf4/5
					Deaf1/Znf567
					Erf.b
					Foxn1/4.b
					Foxtun4/Znf248
					Gata4/5/6/Gata.a
					Hnf4
					Tfap4
C					
	Nr2c	Gabpa		Esrr	E2f5
		Nfat5		Etv	Pitx
D					
	Groucho1/Tl	Nfyb	Irx.a	Gsc	Foxo
	Mnt-r		Myt1l/Znf2	Maf.a/b	Foxtun2
	RelA		Neurog		Noto/Vax1
			Prdtun1/Isx		

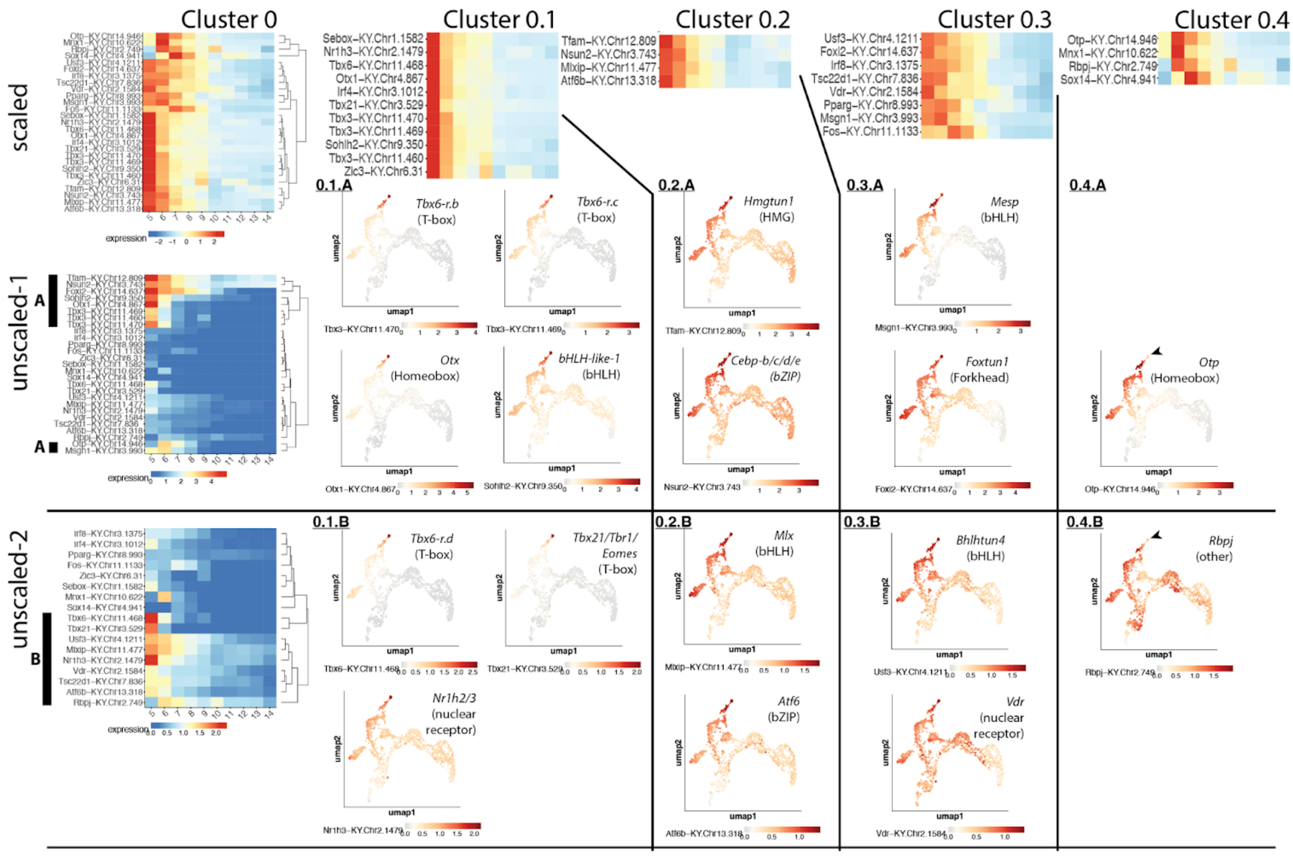

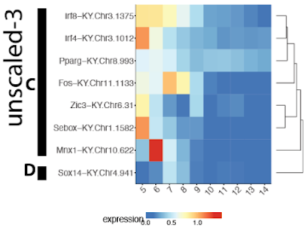

Cluster0				
	0.1	0.2	0.3	0.4
A				
	bHLH-like1	Cebpb/c/d/e	Foxtun1	Otp
	Otx	Hmgtun1	Mesp	
	Tbx6-r.a			
	Tbx6-r.b			
	Tbx6-r.c			
B				
	Nr1h2/3	Atf6	Bhlhtun4/Usf3	Rbpj
	Tbx21/Tbr1/Eomes	Mlx	Tsc22	
	Tbx6-r.d		Vdr.a	
C				
	IRF-like-5		Fos	Mnx
	Prdtun2		IRF-like-7	
	Zic-r.g/ZicL		Ppar	
D				
				Sox30

Appendix Figure S16

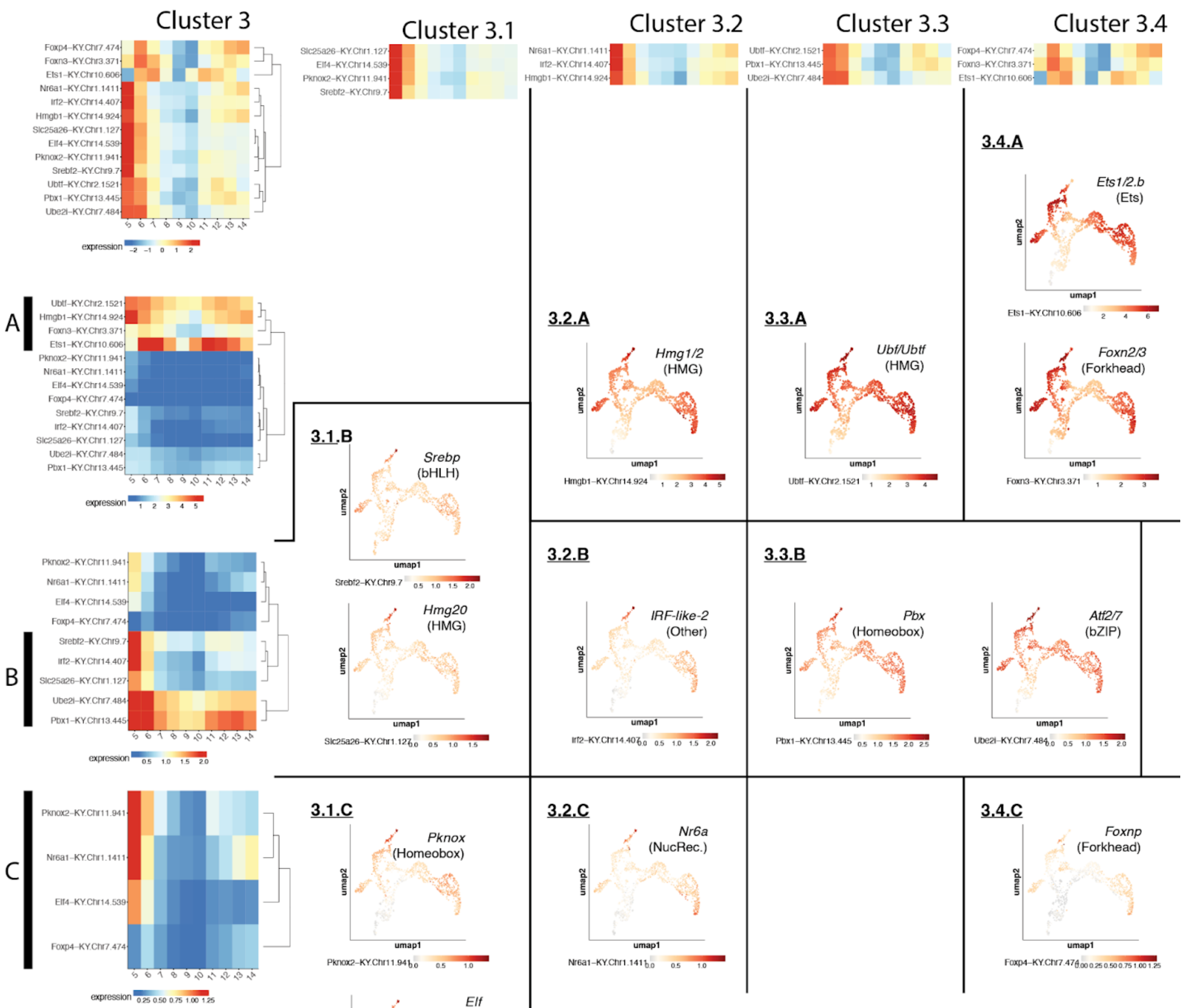

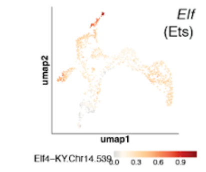

Cluster 3				
**Row Label**	**3.1**	**3.2**	**3.3**	**3.4**
**A**		2	1	3
		Hmg1/2	Ubf/Ubtf	Ets1/2.b
				Foxn2/3
**B**	10	1	7	
	Hmg20	IRF-like-2	Atf2/7	
	Srebp		Pbx	
**C**	2	2		1
	Elf	Nr6a		Foxp/Znf255
	Pknox			

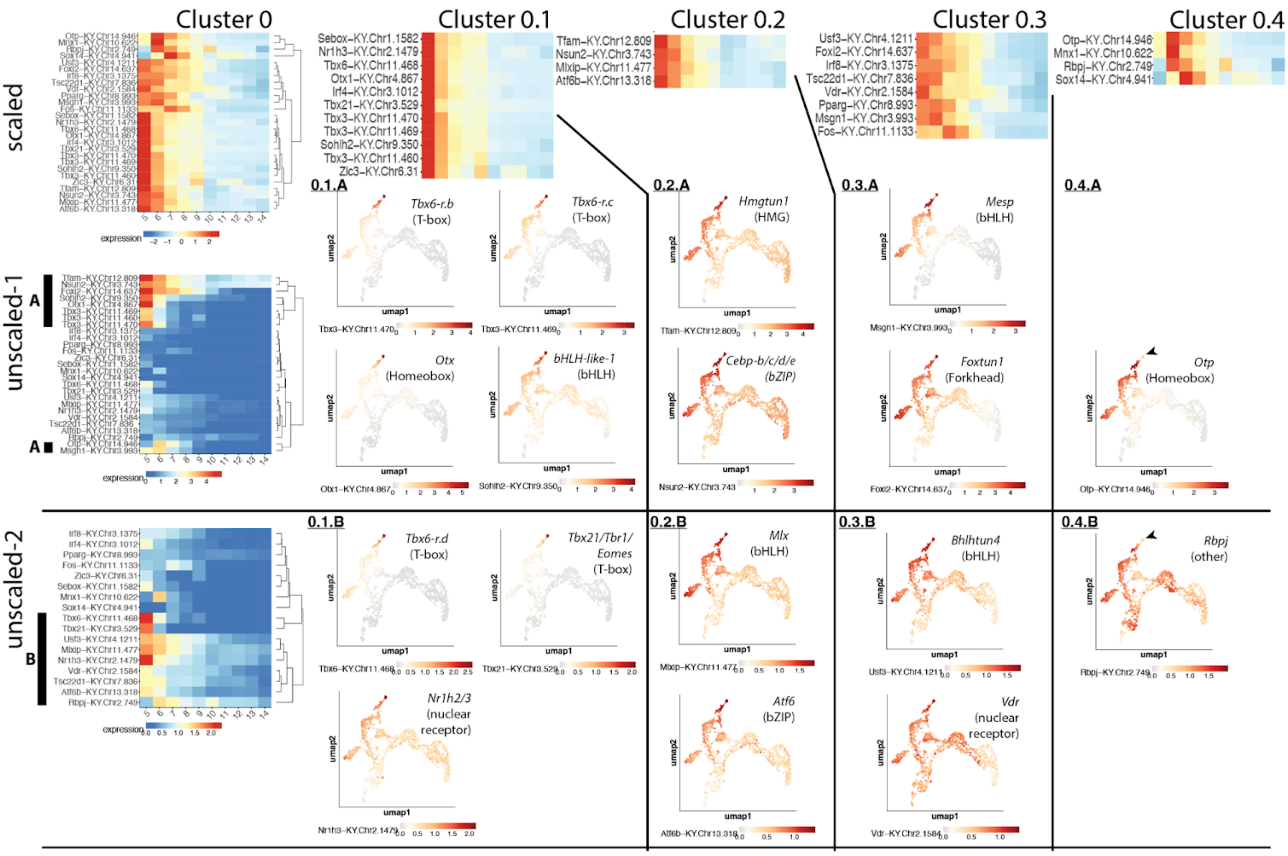

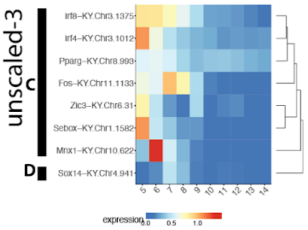

Cluster0				
	0.1	0.2	0.3	0.4
**A**				
	bHLH-like1	Cebpb/c/d/e	Foxtun1	Otp
	Otx	Hmgtun1	Mesp	
	Tbx6-r.a			
	Tbx6-r.b			
	Tbx6-r.c			
**B**				
	Nr1h2/3	Atf6	Bhlhtun4/Usf3	Rbpj
	Tbx21/Tbr1/Eomes	Mlx	Tsc22	
	Tbx6-r.d		Vdr.a	
**C**				
	IRF-like-5		Fos	Mnx
	Prdtun2		IRF-like-7	
	Zic-r.g/ZicL		Ppar	
**D**				
				Sox30

Appendix Figure S17

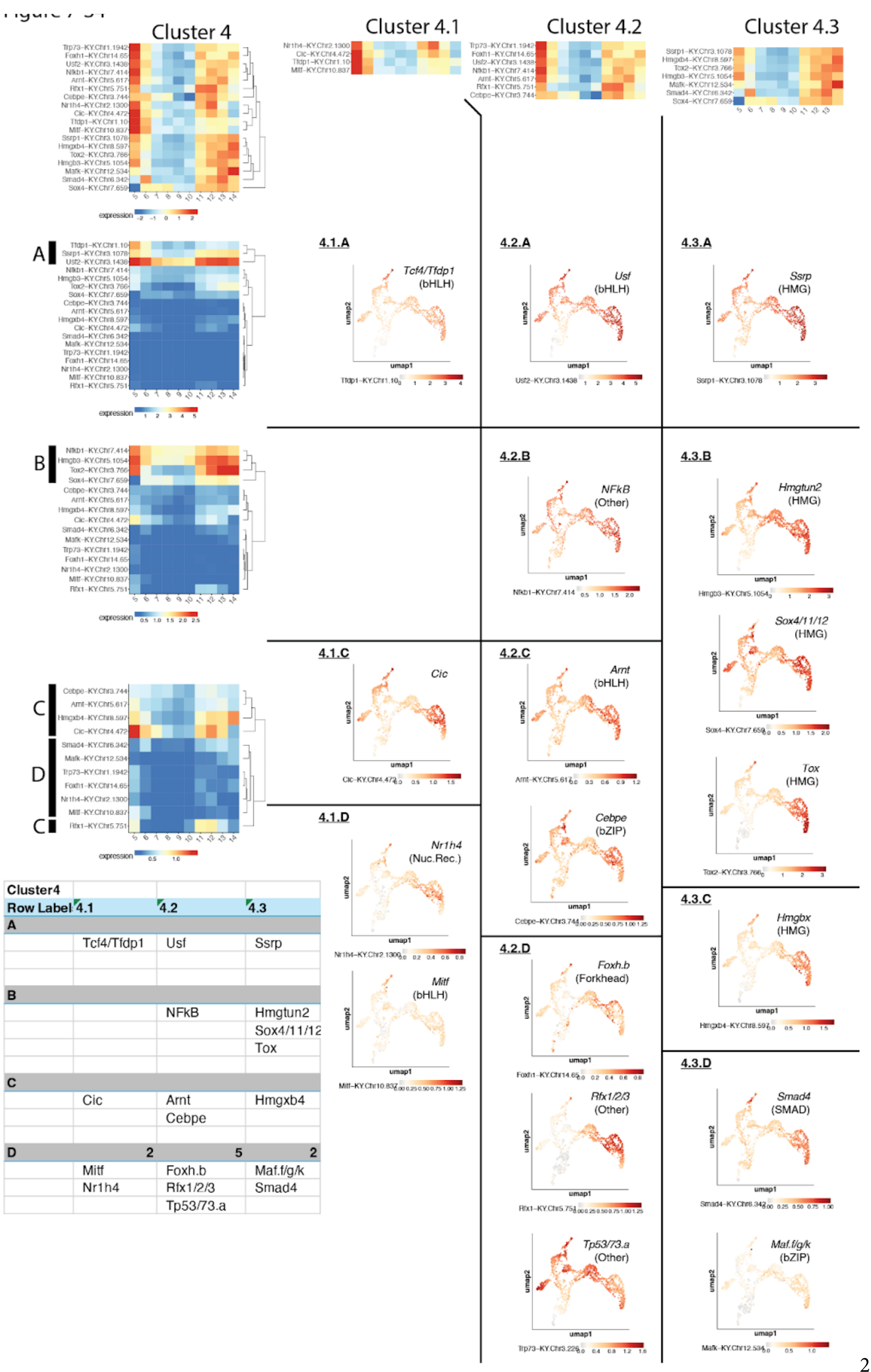

Cluster4			
Row Label	4.1	4.2	4.3
A			
	Tcf4/Tfdp1	Usf	Ssrp
B			
		NFkB	Hmgtun2
			Sox4/11/12
			Tox
C			
	Cic	Arnt	Hmgxb4
		Cebpe	
D	2	5	2
	Mitf	Foxh.b	Maf.f/g/k
	Nr1h4	Rfx1/2/3	Smad4
		Tp53/73.a	

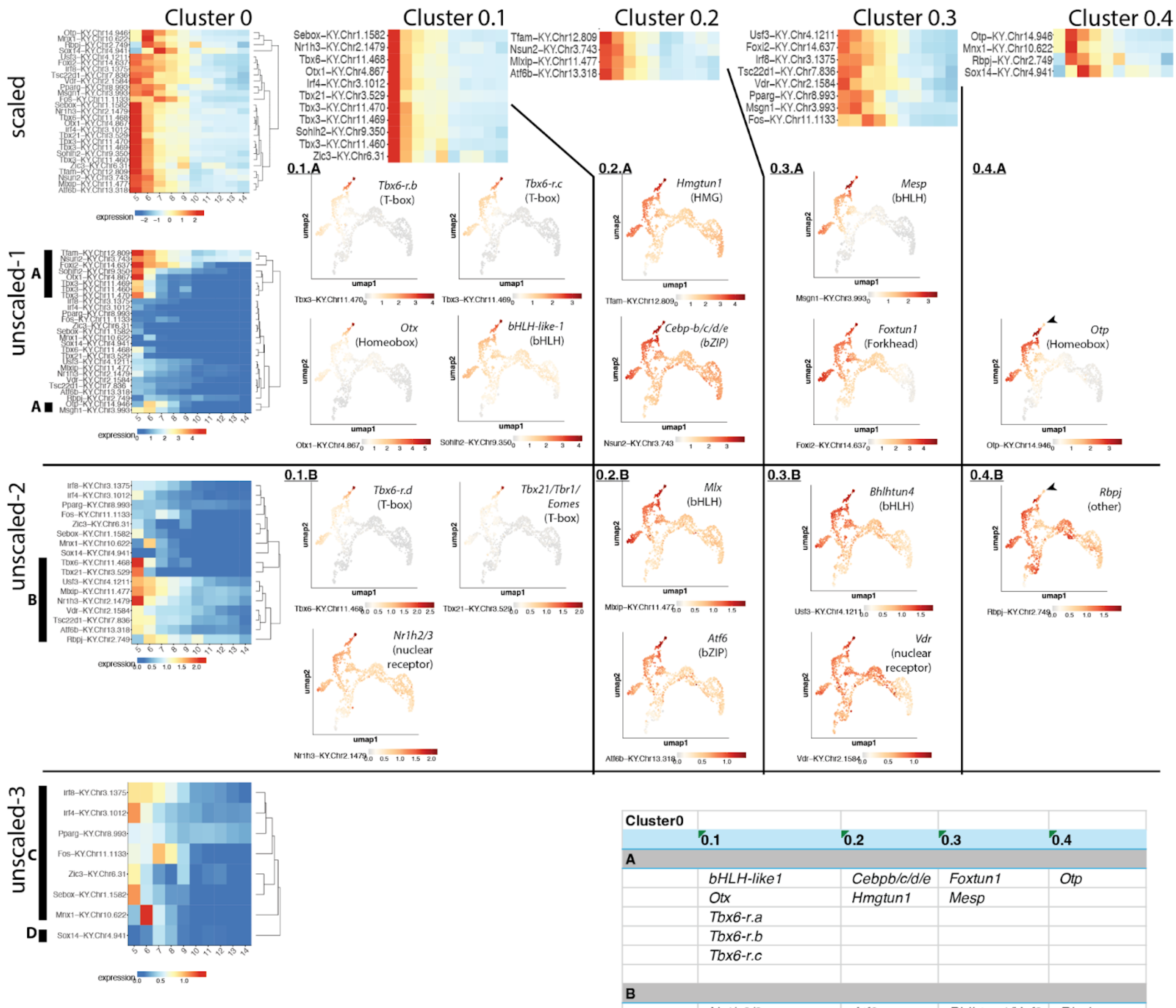

Cluster0				
	0.1	0.2	0.3	0.4
**A**				
	bHLH-like1	*Cebpb/c/d/e*	*Foxtun1*	*Otp*
	Otx	*Hmgtun1*	*Mesp*	
	Tbx6-r.a			
	Tbx6-r.b			
	Tbx6-r.c			
**B**				
	Nr1h2/3	*Atf6*	*Bhlhtun4/Usf3*	*Rbpj*
	Tbx21/Tbr1/Eomes	*Mlx*	*Tsc22*	
	Tbx6-r.d		*Vdr.a*	
**C**				
	IRF-like-5		*Fos*	*Mnx*
	Prdtun2		*IRF-like-7*	
	Zic-r.g/ZicL		*Ppar*	
**D**				
				Sox30

Appendix Figure S18

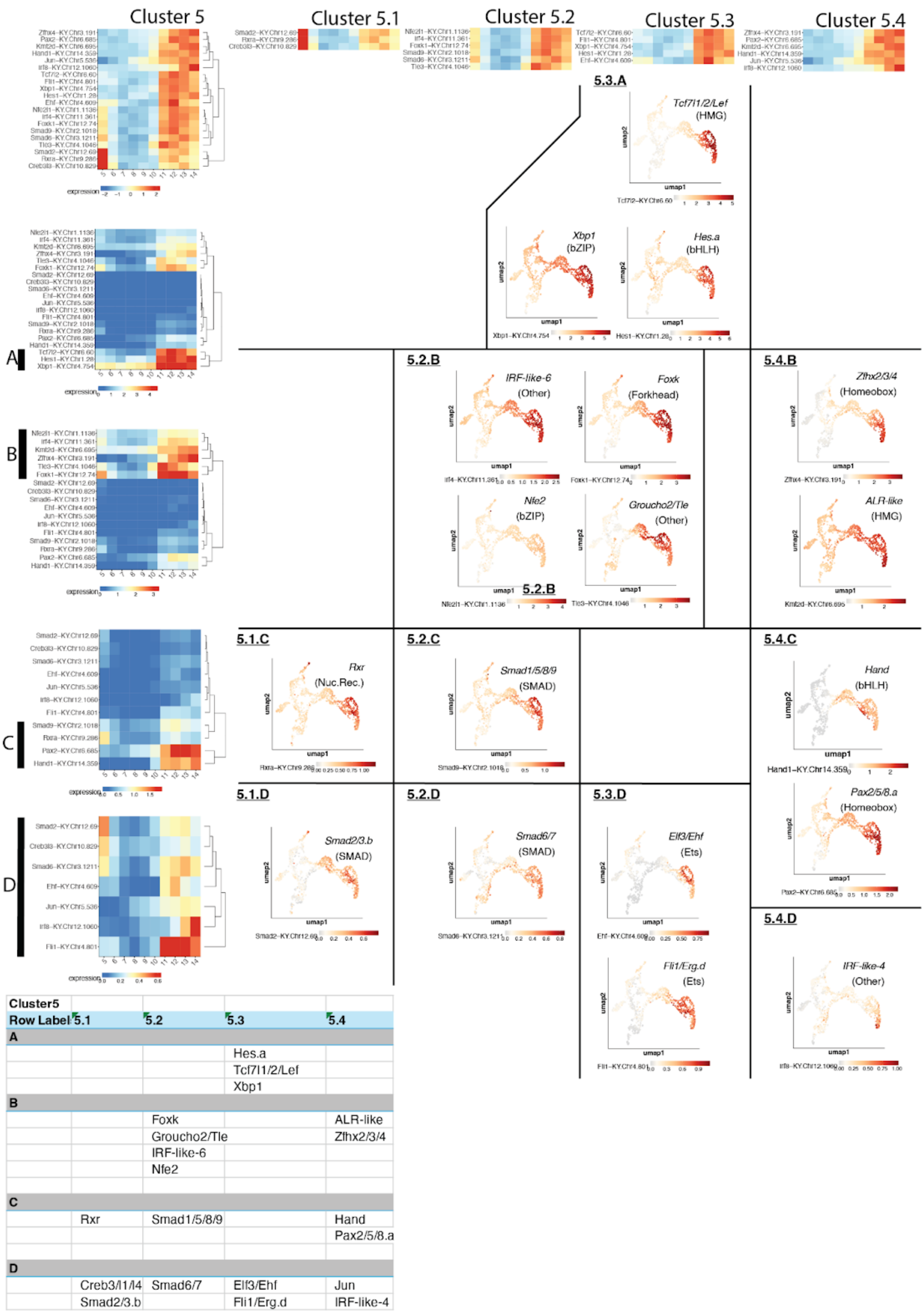

Cluster5				
**Row Label**	**5.1**	**5.2**	**5.3**	**5.4**
**A**				
			Hes.a	
			Tcf7l1/2/Lef	
			Xbp1	
**B**				
		Foxk		ALR-like
		Groucho2/Tle		Zfhx2/3/4
		IRF-like-6		
		Nfe2		
**C**				
	Rxr	Smad1/5/8/9		Hand
				Pax2/5/8.a
**D**				
	Creb3/l1/l4	Smad6/7	Elf3/Ehf	Jun
	Smad2/3.b		Fli1/Erg.d	IRF-like-4

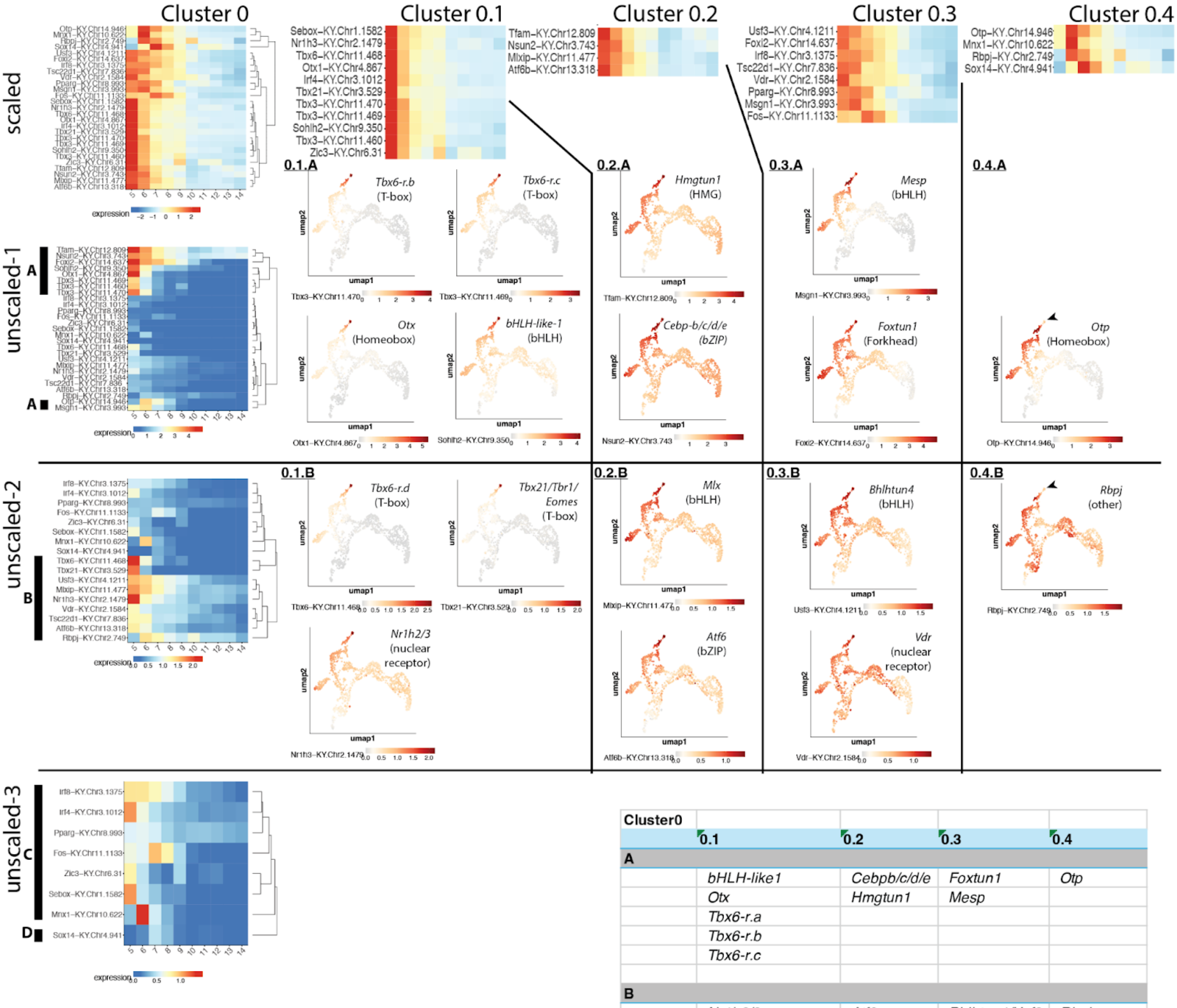

Cluster0				
	0.1	0.2	0.3	0.4
**A**				
	bHLH-like1	*Cebpb/c/d/e*	*Foxtun1*	*Otp*
	Otx	*Hmgtun1*	*Mesp*	
	Tbx6-r.a			
	Tbx6-r.b			
	Tbx6-r.c			
**B**				
	Nr1h2/3	*Atf6*	*Bhlhtun4/Usf3*	*Rbpj*
	Tbx21/Tbr1/Eomes	*Mlx*	*Tsc22*	
	Tbx6-r.d		*Vdr.a*	
**C**				
	IRF-like-5		*Fos*	*Mnx*
	Prdtun2		*IRF-like-7*	
	Zic-r.g/ZicL		*Ppar*	
**D**				
				Sox30

Appendix Figure S19

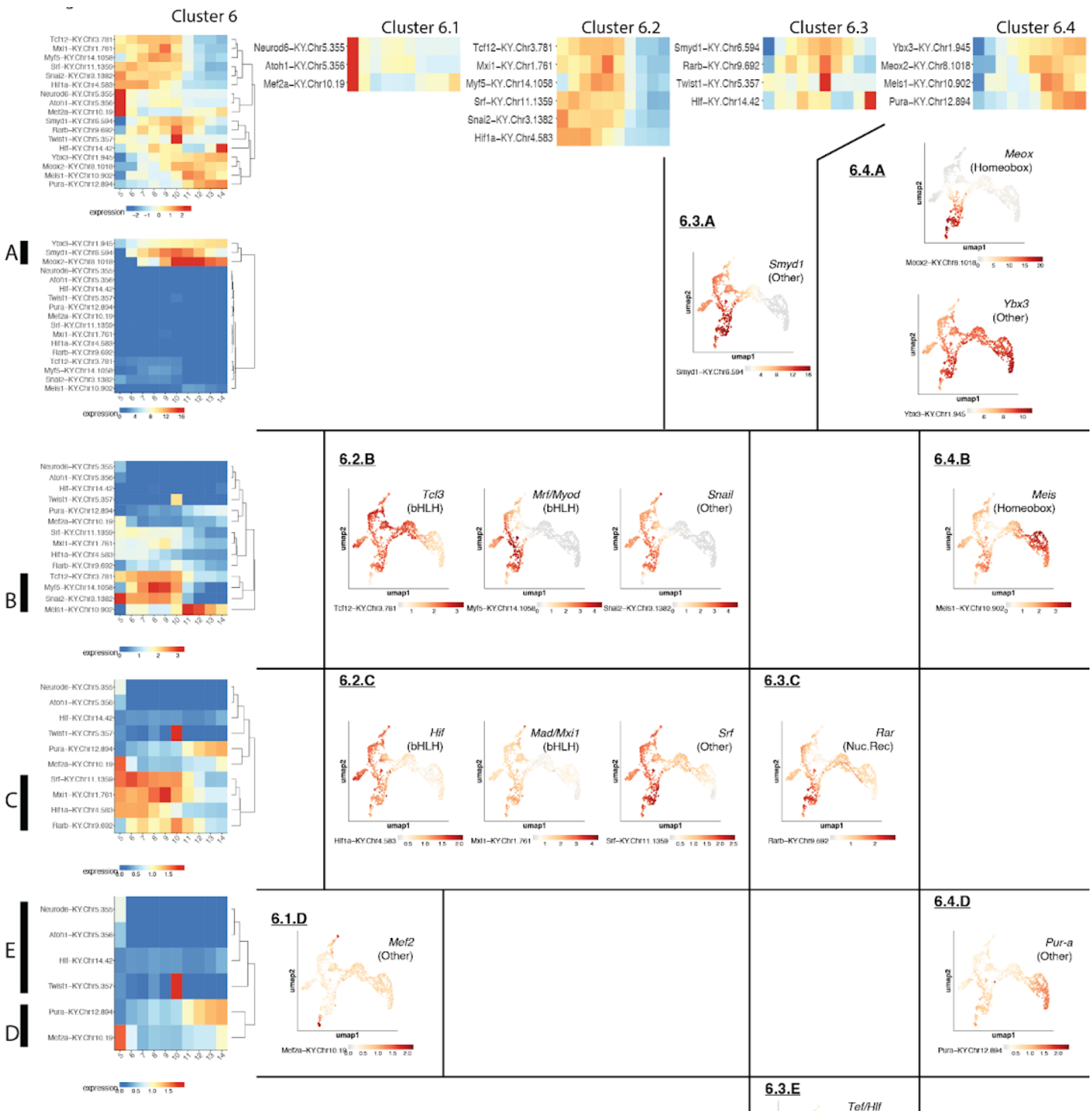

Cluster6				
**Row Labels**	**6.1**	**6.2**	**6.3**	**6.4**
**A**				
			Smyd1/Znf438	Meox
				Ybx3
**B**				
		Mrf/Myod/Myf5		Meis
		Snail		
		Tcf3		
**C**				
		Hif	Rar	
		Mad/Mxi1		
		Srf		
**D**				
	Mef2			Pur-a
**E**				
	Twist-r.a/Atoh1		Tef/Hlf	
	Twist-r.b/Neurod6		Twist-r.c/Twist1	

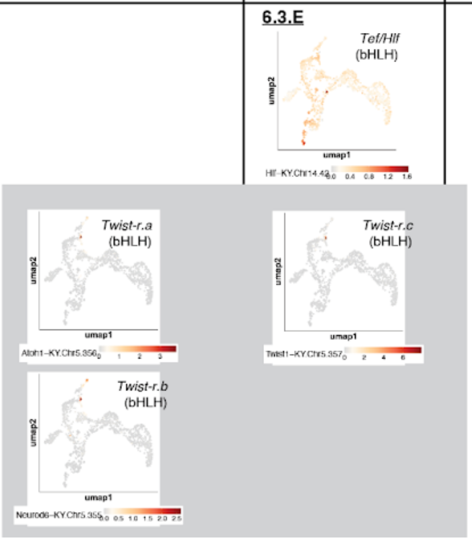

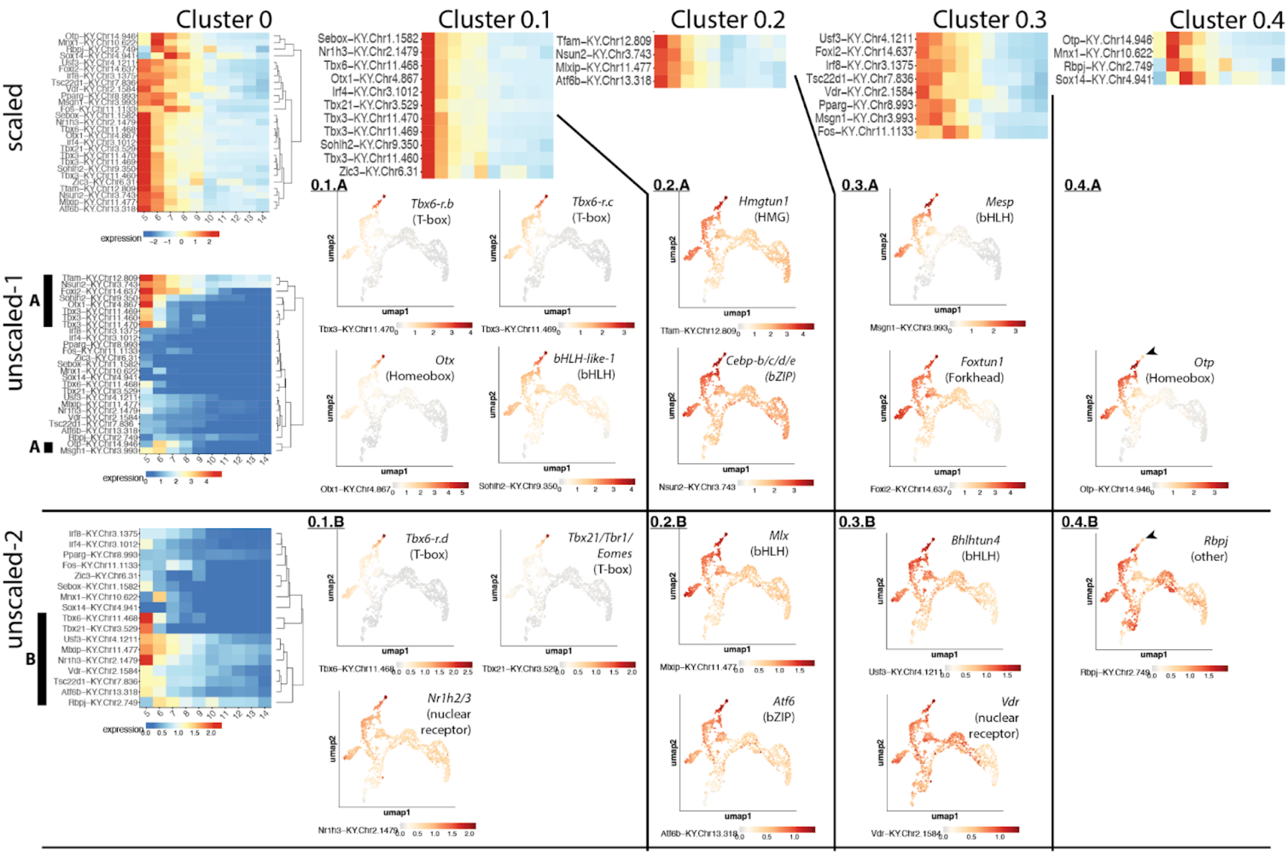

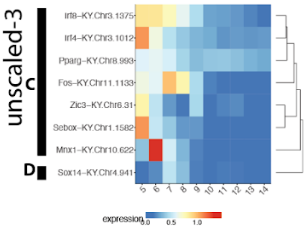

Cluster0				
	0.1	0.2	0.3	0.4
A				
	bHLH-like1	Cebpb/c/d/e	Foxtun1	Otp
	Otx	Hmgtun1	Mesp	
	Tbx6-r.a			
	Tbx6-r.b			
	Tbx6-r.c			
B				
	Nr1h2/3	Atf6	Bhlhtun4/Usf3	Rbpj
	Tbx21/Tbr1/Eomes	Mlx	Tsc22	
	Tbx6-r.d		Vdr.a	
C				
	IRF-like-5		Fos	Mnx
	Prdtun2		IRF-like-7	
	Zic-r.g/ZicL		Ppar	
D				
				Sox30

Appendix Figure S20

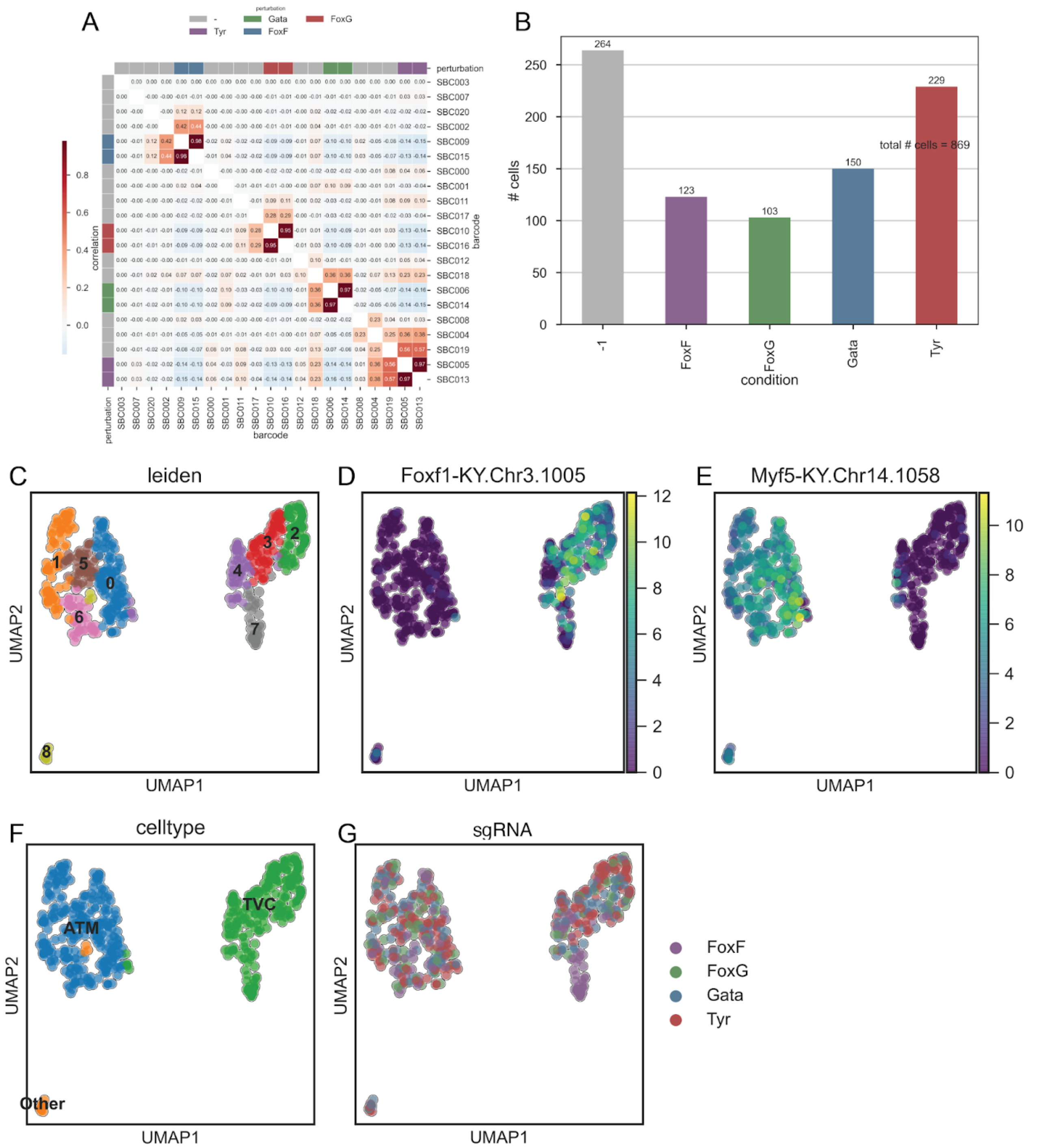

Appendix Figure S21 - Figure 7-S8. Sample barcode recovery in the CRISPR x scRNA-seq experiment.

A. Correlation plot between co-electroporated pairs of sample barcoding constructs.
B. Distribution of sample assignment following barcode recovery.
C. UMAPs showing the distribution of individual single cell transcriptomes across Leiden clusters.
D-F. Expression of key cardiopharyngeal (Foxf, D) and anterior tail muscle (Myod/Myf5, E) markers allowing annotation of Leiden clusters as cell types (F). Distribution of individual CRISPR/Cas9 perturbations across single cell transcriptomes. Note the offset position of Foxf^{CRISPR} transcriptomes (purple).

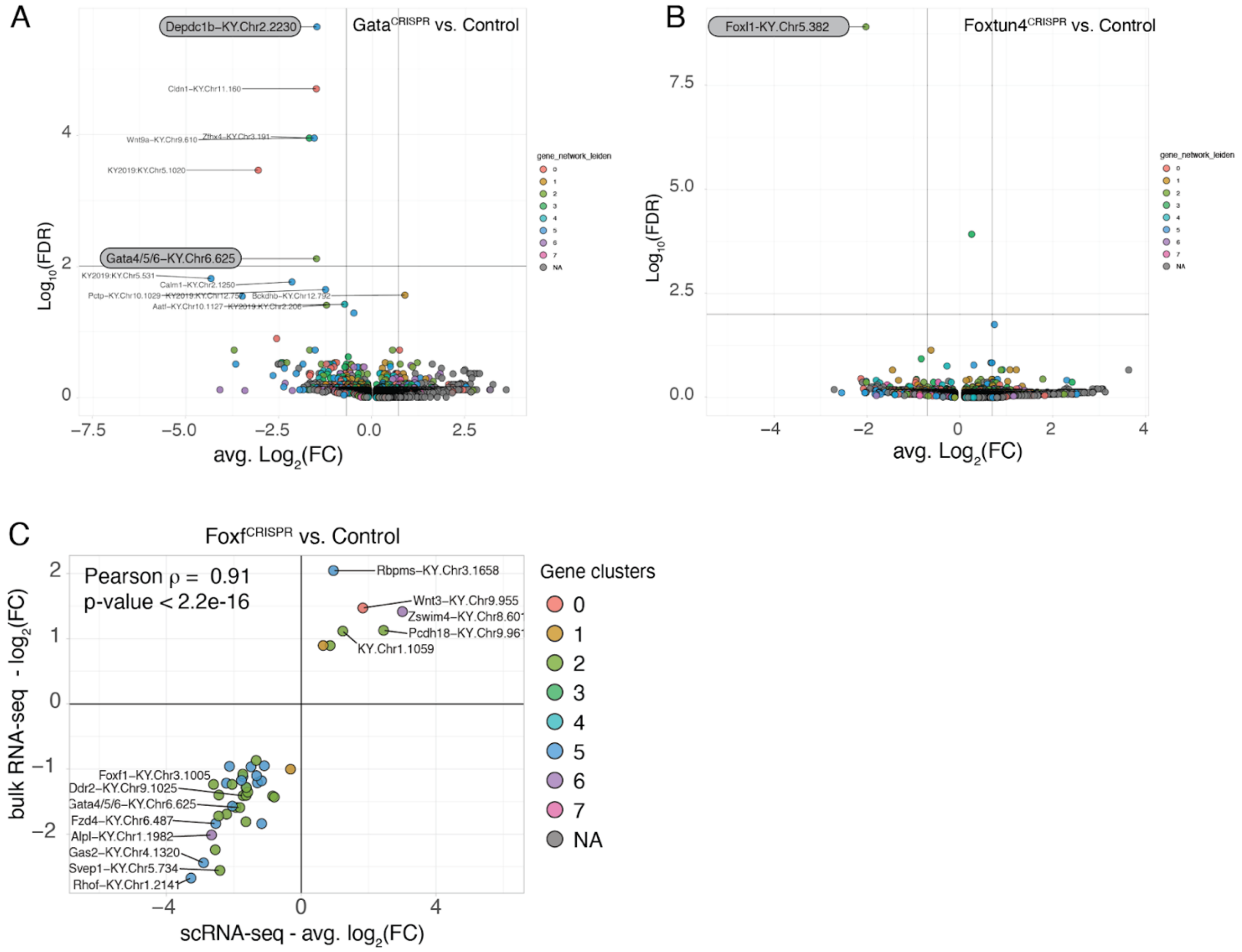

<u>Appendix Figure S22 - Figure 7-S9. CRISPR/Cas9-mediated mutagenesis targeting *Foxf*, *Gata4/5/6*, and *Foxtun4*, followed by scRNA-seq of FACS-purified cardiopharyngeal lineage cellss.</u>

A. Volcano plot of log$_2$ fold gene expression change in *Gata4/5/6*^{CRISPR} vs. Control conditions with colors of genes corresponding to their Leiden cluster identity.

B. Volcano plot of log$_2$ fold gene expression change in *Foxtun4*^{CRISPR} vs Control conditions.

C. Log$_2$ fold-change for top DE genes in *Foxf*^{CRISPR} vs control comparing the scRNA-seq from this paper to the bulk RNA-seq of FACS-sorted cells from Racioppi et al. 2019, with significant correlation values indicated.

In A-C, color codes correspond to the gene clusters defined as in Figure 3B.

A

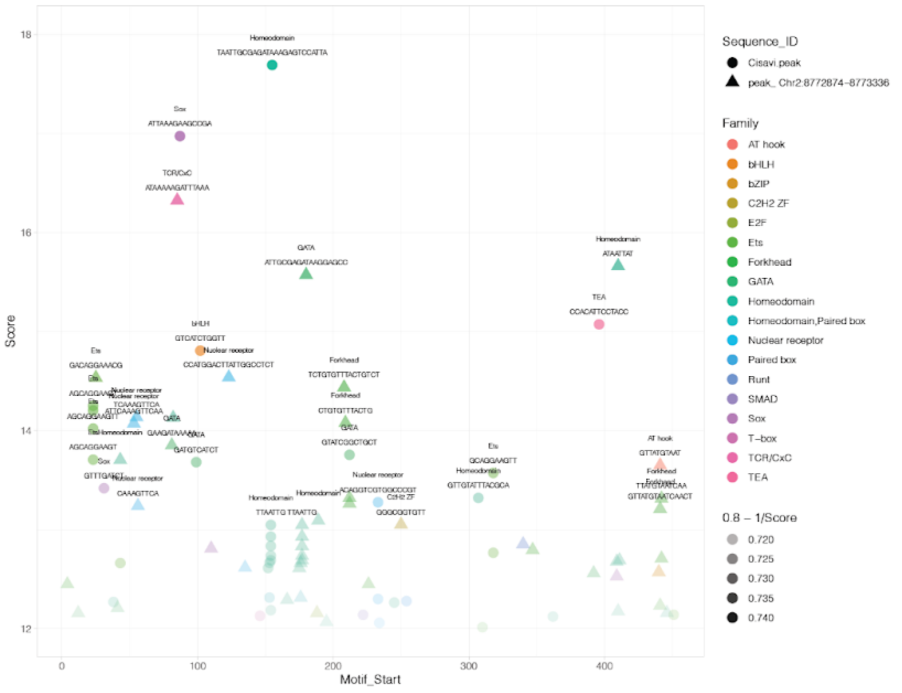

B >Cirobu.Depdc1b_promoter
CTACTACATGTATTCAAATTAAAGTTAAAATGTTTAAAACGTGCGTATGATTCAATGTG-
TATGCAGTTGTTGCTTTCGTGTTTTCGTTGGTTTGTTTACAAGCATGGTAATTAGGAGCAAACATTTAGGTTGAAGGTGTTTGT
ACGTAGTCGCACACTTTGCTGTTTTATCCGCCCTGTTGTTTACGAAAGTTTACAAAGGTTTTATTTCATTGCTGGCTTGTTTAC
TATTACGAGGTCCTTGTTAATGGGCACCATAGATCTTTAGTTCGGTACCATTGTTAATTGATCGCGGCACGACTATGACGTCAC
CGCTTGCGCGTGCTTAAAGCGCGAGACAATAGCGATAGAGAAAGGAGAATGCGGTCGCAGCGCGCGATTTTAAACGTCGCTTTT
TACGAAAAATGCAGAACATAGTTGTCGGTTCGCTGACGAGTAGGAACGCACAGTGCAAGTGCTTTAGTAGAGTGGGAGTGTGTT
GTATATGATACAGT

C

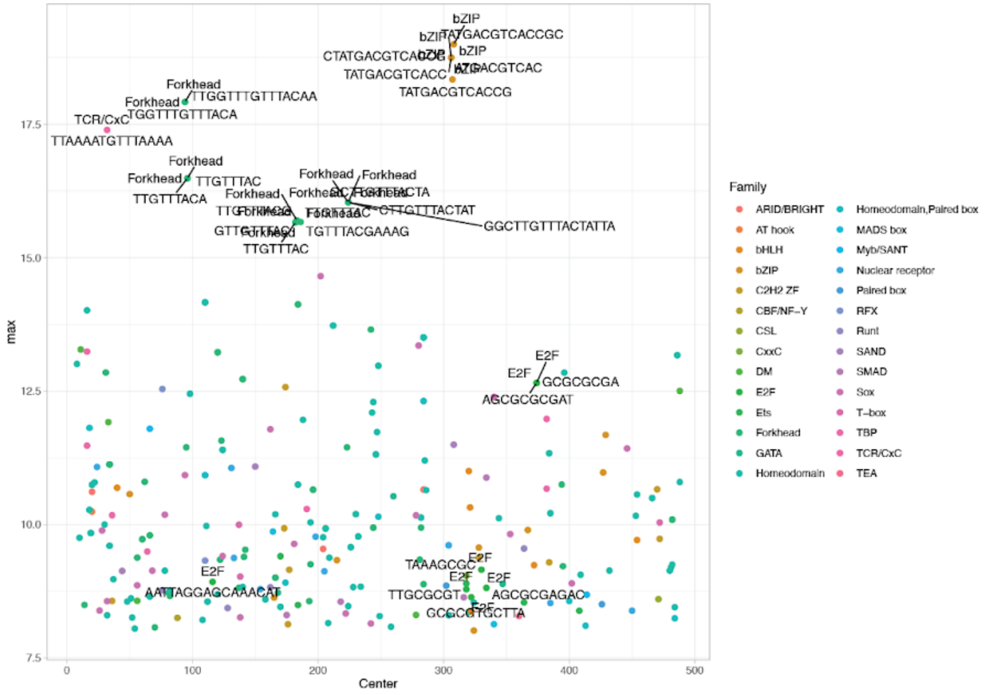

__Appendix Figure S23 - Figure 8-S1. Putative Transcription Factor Binding Motifs in the Depdc1b enhancer, which are conserved between Ciona robusta and Ciona savignyi, and in the Depdc1b proximal promoter region.__

. The sequences used correspond to the alignment shown in Figure 4, and the x axis correspond to the position on each sequence. The y axis shows the motif scores obtained

from Cis-BP, using motifs inferred by homology for Ciona homologs of transcription factors indicated as color codes. Transparency is also inversely correlated with motif score. The shapes correspond to the species, *Ciona savignyi* (dots) or *Ciona robusta* (triangles).

. Sequence of the proximal promoter region of Cirobu.Depdc1b with putative E2F motifs highlighted.

. Putative transcription factor binding sites, inferred by Cis-BP, with scores (y axis) mapped onto the position of the sequence (x axis) for indicated TF families.
