# Supplementary figures and images for: Cell cycle-driven transcriptome maturation confers multilineage competence to cardiopharyngeal progenitors
Source: EMBO J. 2025 Nov 3;44(24):7649–76. doi: 10.1038/s44318-025-00613-y (PMC12705688; doi:10.1038/s44318-025-00613-y)

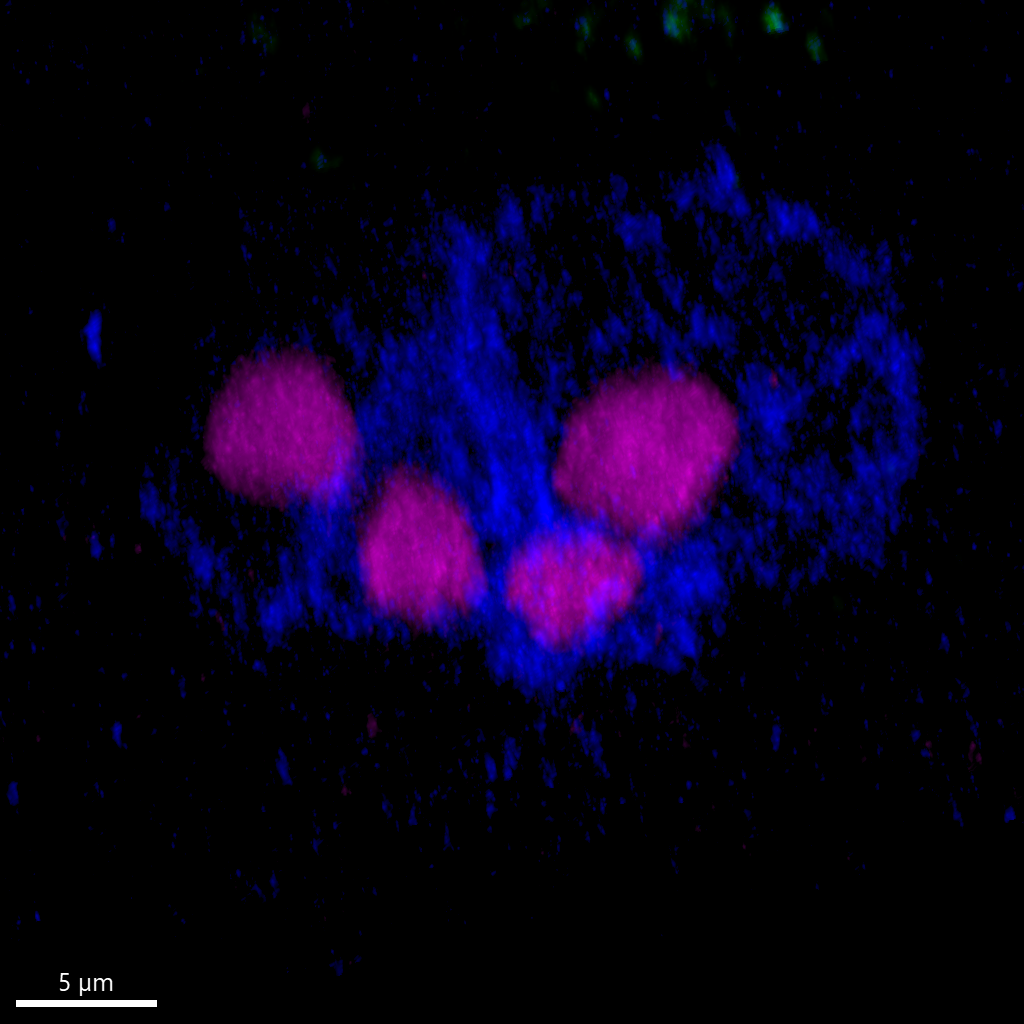

Supplement: Supplementary file 11 — Source data Fig. 1 [file 44318_2025_613_MOESM11_ESM.zip › Figure 1/1H/Figure1H. Cdc25HA_12.5H.tif]

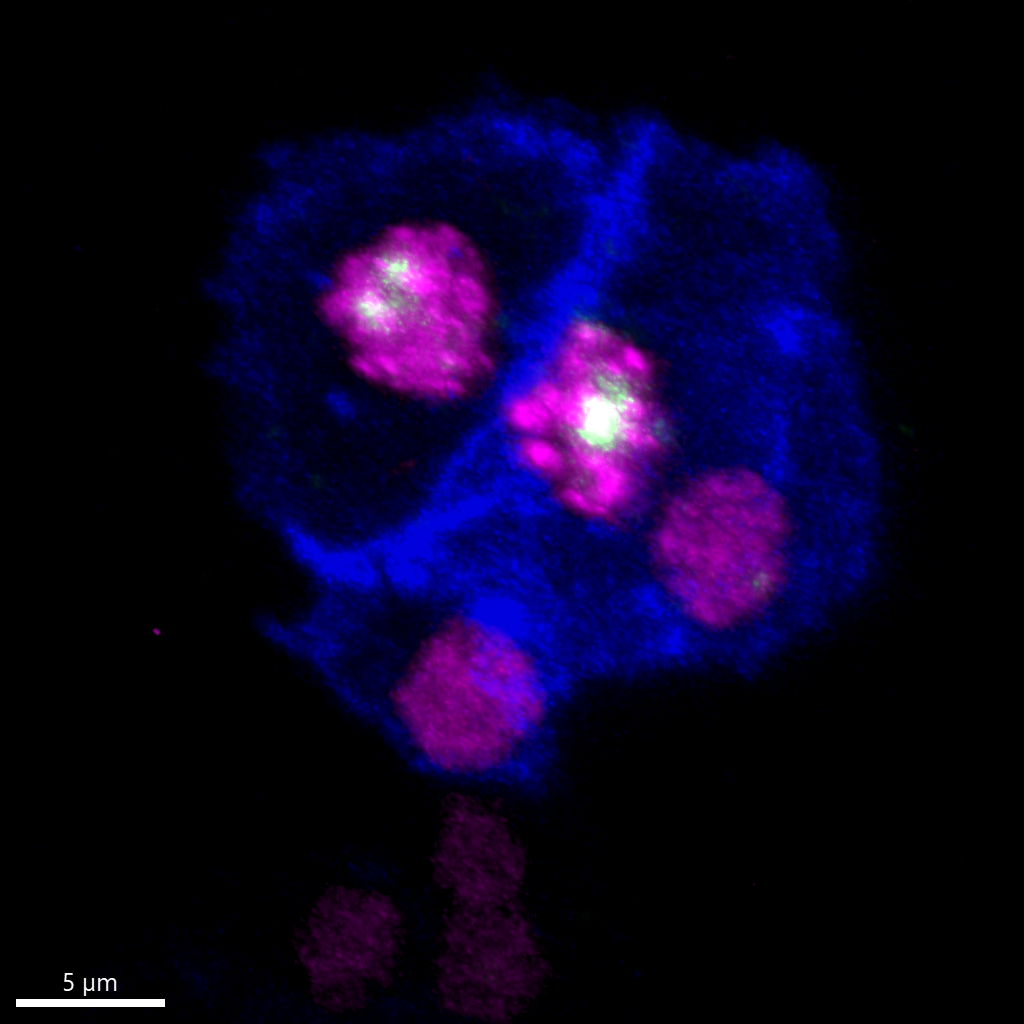

Supplement: Supplementary file 11 — Source data Fig. 1 [file 44318_2025_613_MOESM11_ESM.zip › Figure 1/1H/Figure1H'. Cdc25HA_14.5H.tif]

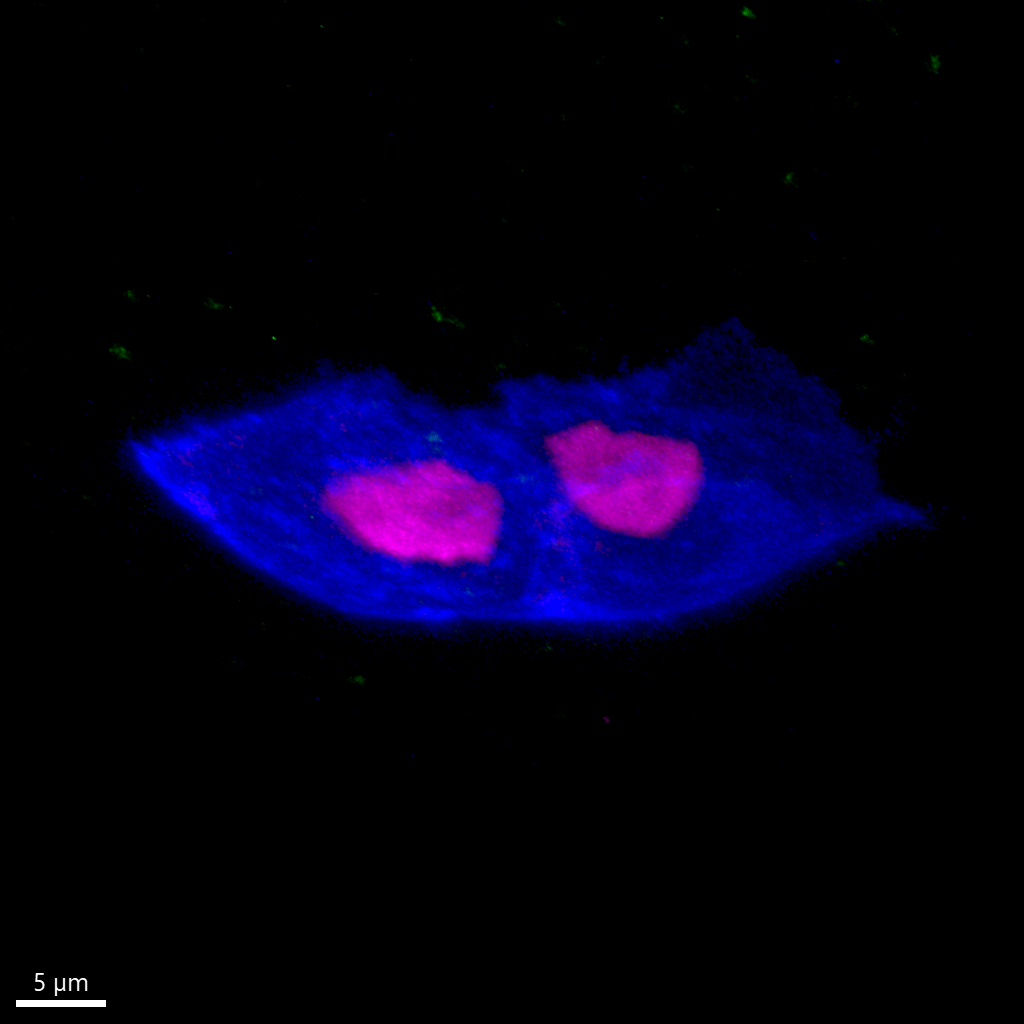

Supplement: Supplementary file 11 — Source data Fig. 1 [file 44318_2025_613_MOESM11_ESM.zip › Figure 1/1F/Figure1F'. Wee1HA_14.5H.tif]

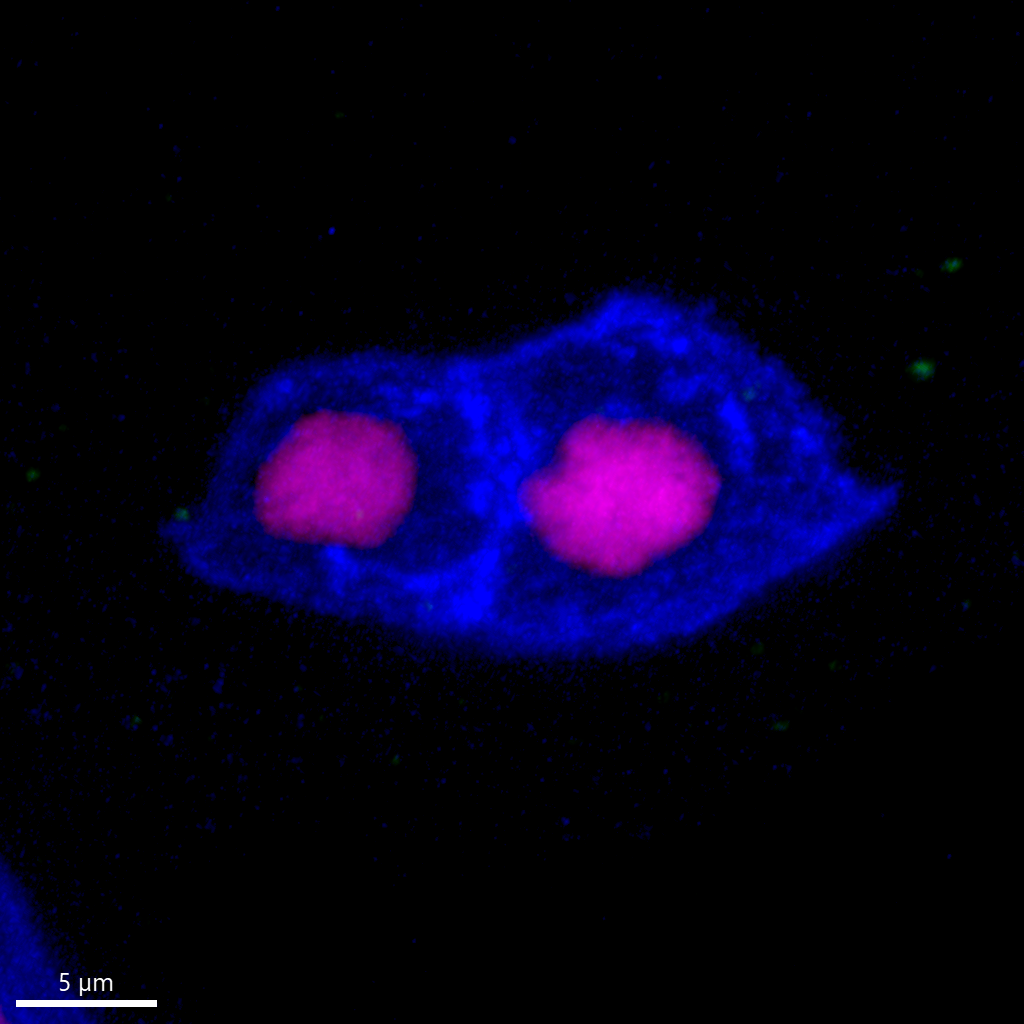

Supplement: Supplementary file 11 — Source data Fig. 1 [file 44318_2025_613_MOESM11_ESM.zip › Figure 1/1F/Figure1F. Wee1HA_12.5H.tif]

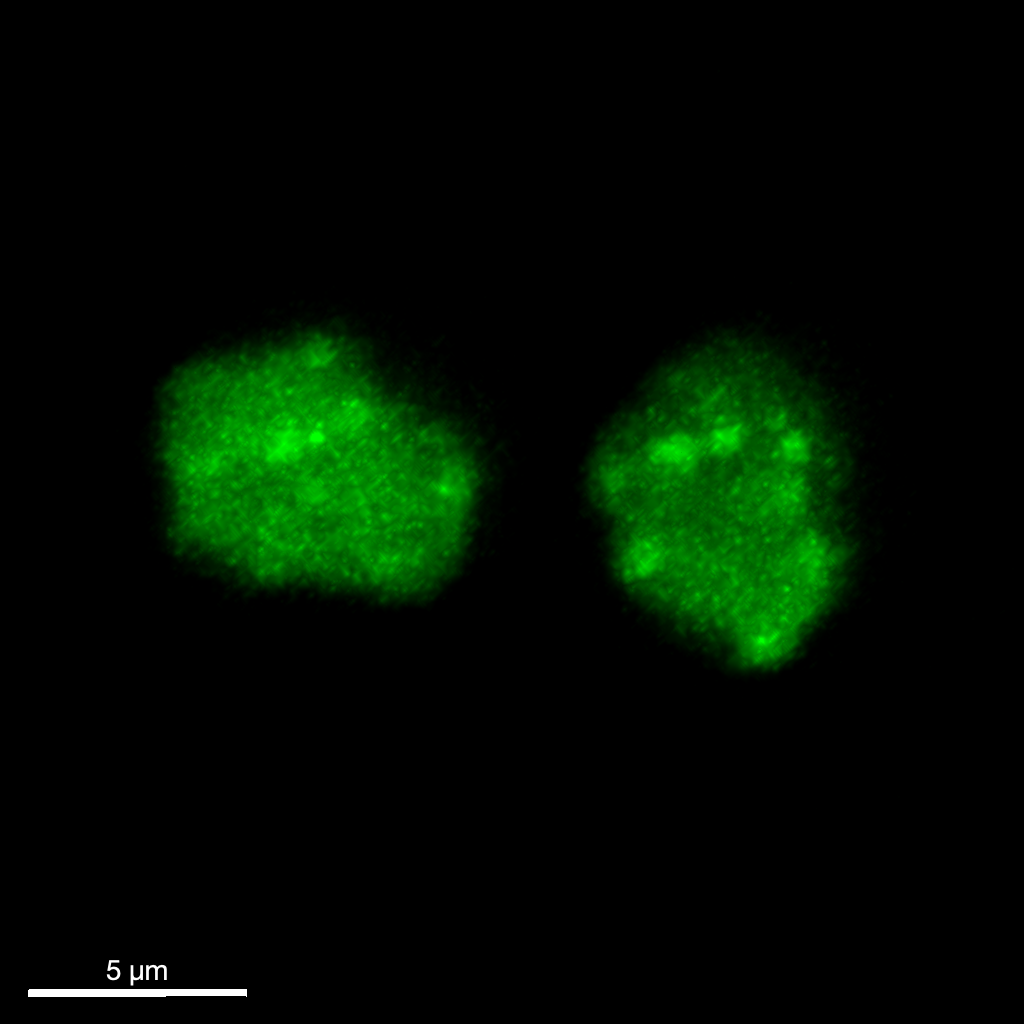

Supplement: Supplementary file 11 — Source data Fig. 1 [file 44318_2025_613_MOESM11_ESM.zip › Figure 1/1L/20210506_EP4_3rdSP8_10.0H_[ii21_#4_10.0H_41_Image_22]_2023-09-28T12-58-56.761.tif]

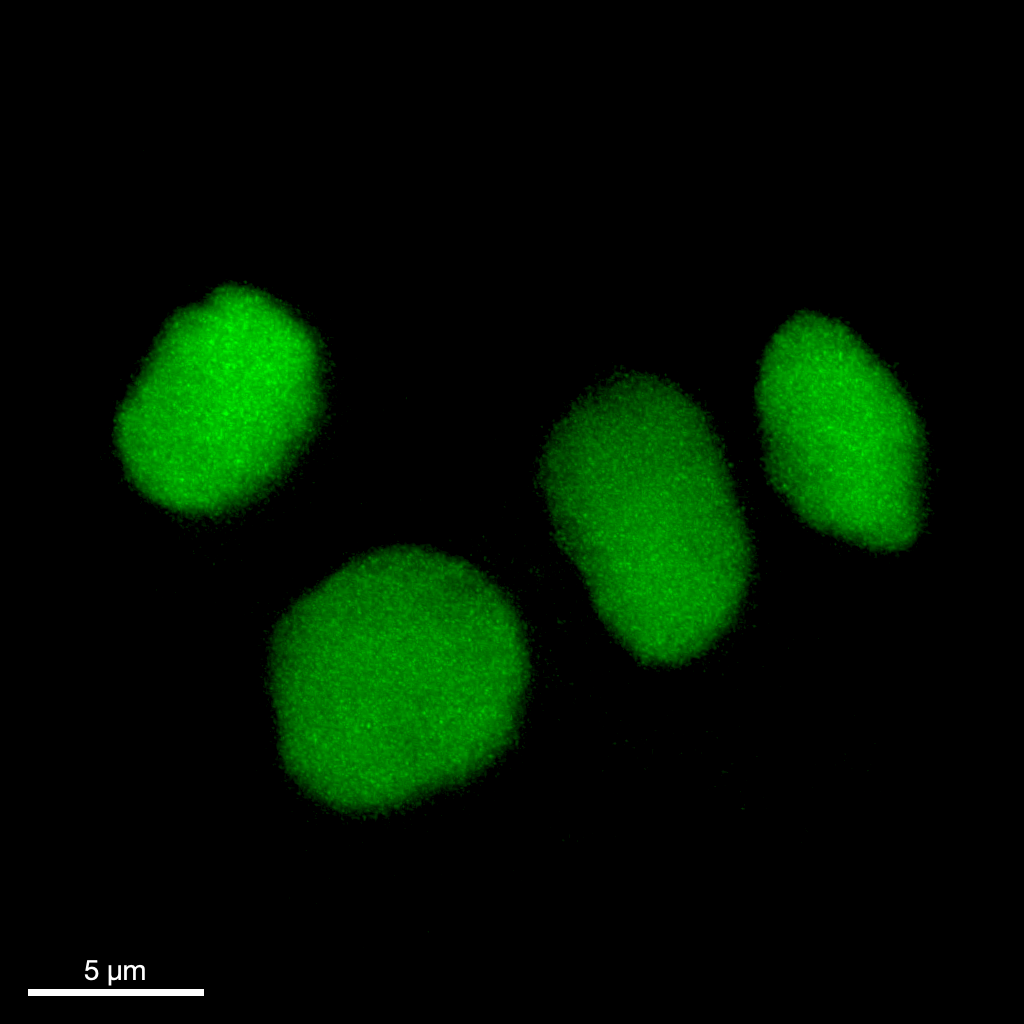

Supplement: Supplementary file 11 — Source data Fig. 1 [file 44318_2025_613_MOESM11_ESM.zip › Figure 1/1L/20210419 S phase SP8 2nd_[ii10_#6_8.5hpf_image5_1024_Image_11]_2023-09-28T12-52-23.506.tif]

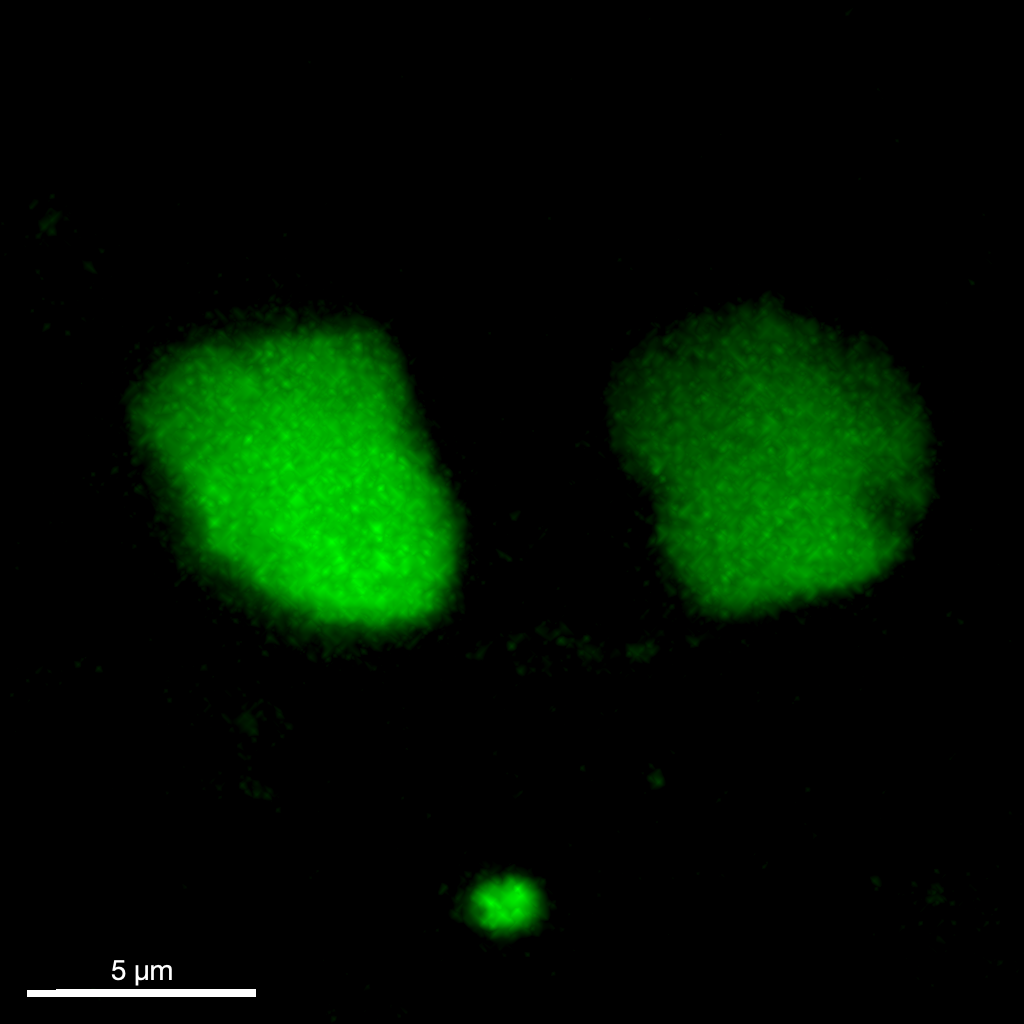

Supplement: Supplementary file 11 — Source data Fig. 1 [file 44318_2025_613_MOESM11_ESM.zip › Figure 1/1L/20210623_EP6_2nd_12H_[ii20_EP6_12.0H_19_Image_21]_2023-09-28T13-01-43.954.tif]

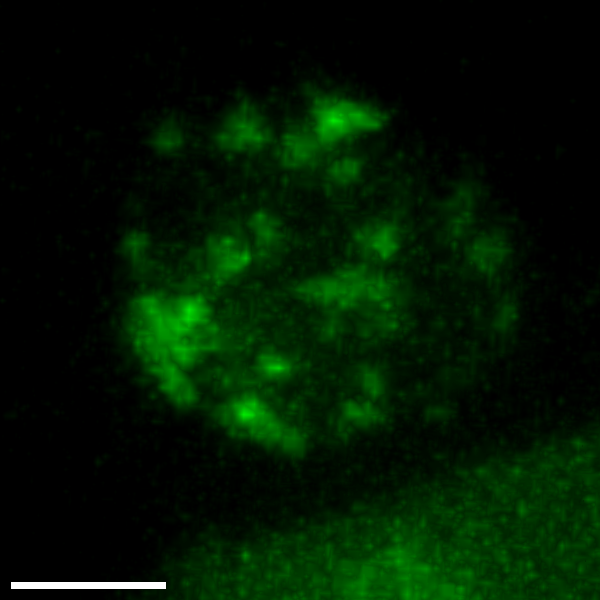

Supplement: Supplementary file 11 — Source data Fig. 1 [file 44318_2025_613_MOESM11_ESM.zip › Figure 1/1K/20210507_EP4_4thSP8_10.0H_[ii3_#4_10.0H_49_Image_4]_2021-05-19T13-59-31.363(midS)scalebar2.5.tif]

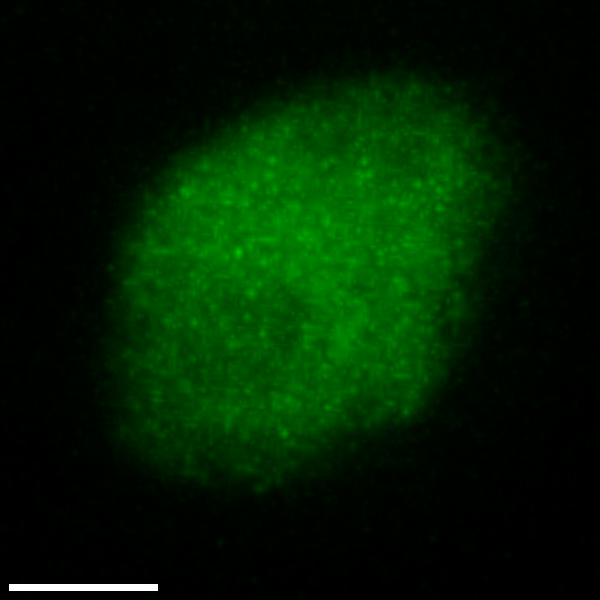

Supplement: Supplementary file 11 — Source data Fig. 1 [file 44318_2025_613_MOESM11_ESM.zip › Figure 1/1K/20210503_EP4_1stSP8_8.5H_[ii10_#1_8.5H_11_Image_11]_2021-05-19T13-15-06.601(Gphase)scalebar2.5.tif]

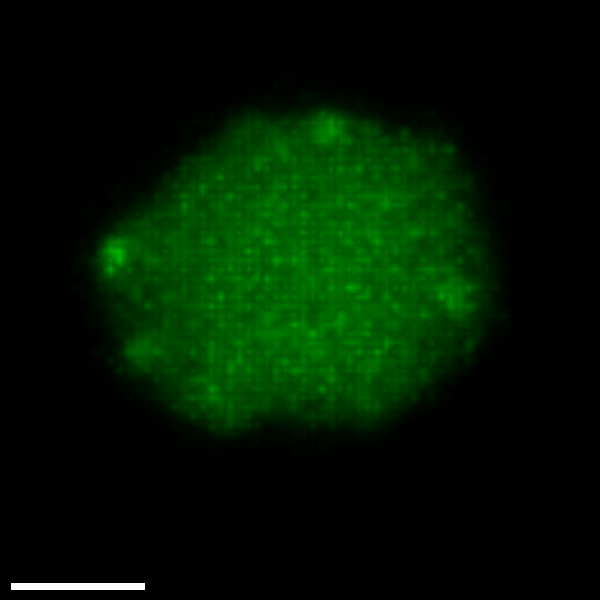

Supplement: Supplementary file 11 — Source data Fig. 1 [file 44318_2025_613_MOESM11_ESM.zip › Figure 1/1K/20210511_EP4_6thSP8_11.0H_6to31_[ii28_#6_11.0H_30_Image_29]_2021-05-19T12-01-31.876(EntryExit)scalebar2.5.tif]

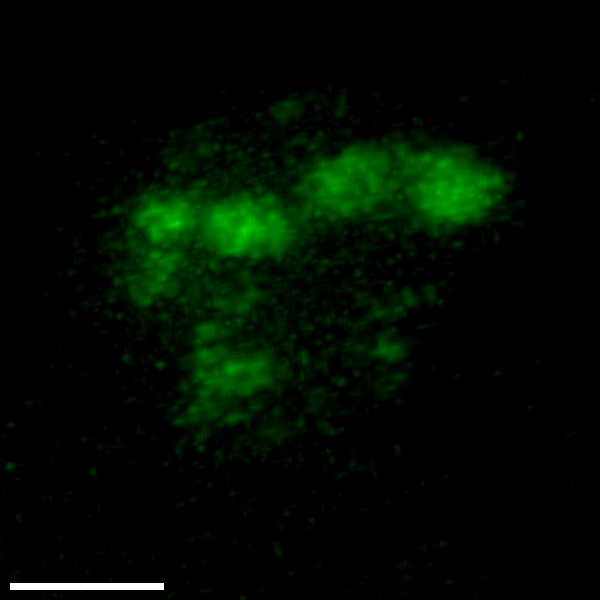

Supplement: Supplementary file 11 — Source data Fig. 1 [file 44318_2025_613_MOESM11_ESM.zip › Figure 1/1K/20210510_EP4_5thSP8_11.0H_[ii6_#6_11.0H_5_Image_7]_2021-05-20T13-31-32.617(lateS)scalebar2.5.tif]

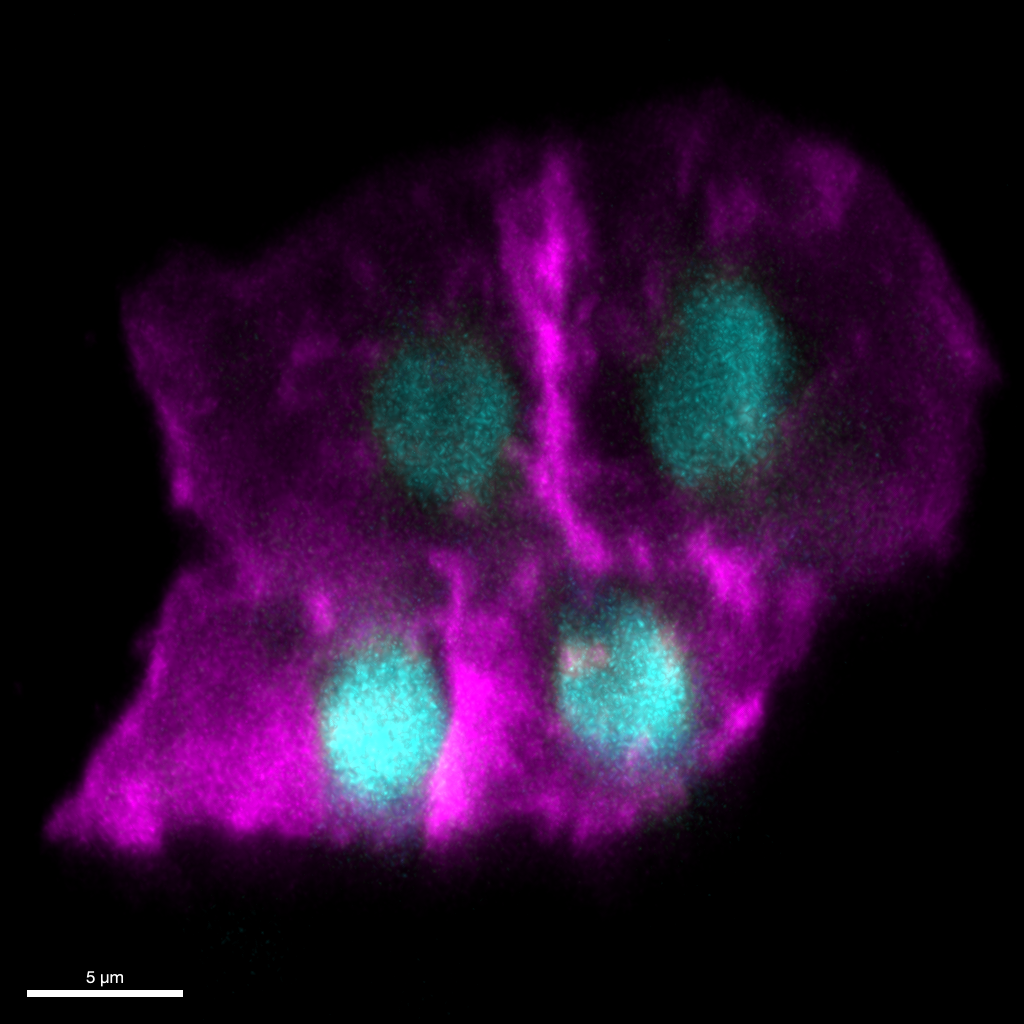

Supplement: Supplementary file 11 — Source data Fig. 1 [file 44318_2025_613_MOESM11_ESM.zip › Figure 1/1B/1B_FHP_STVC.tif]

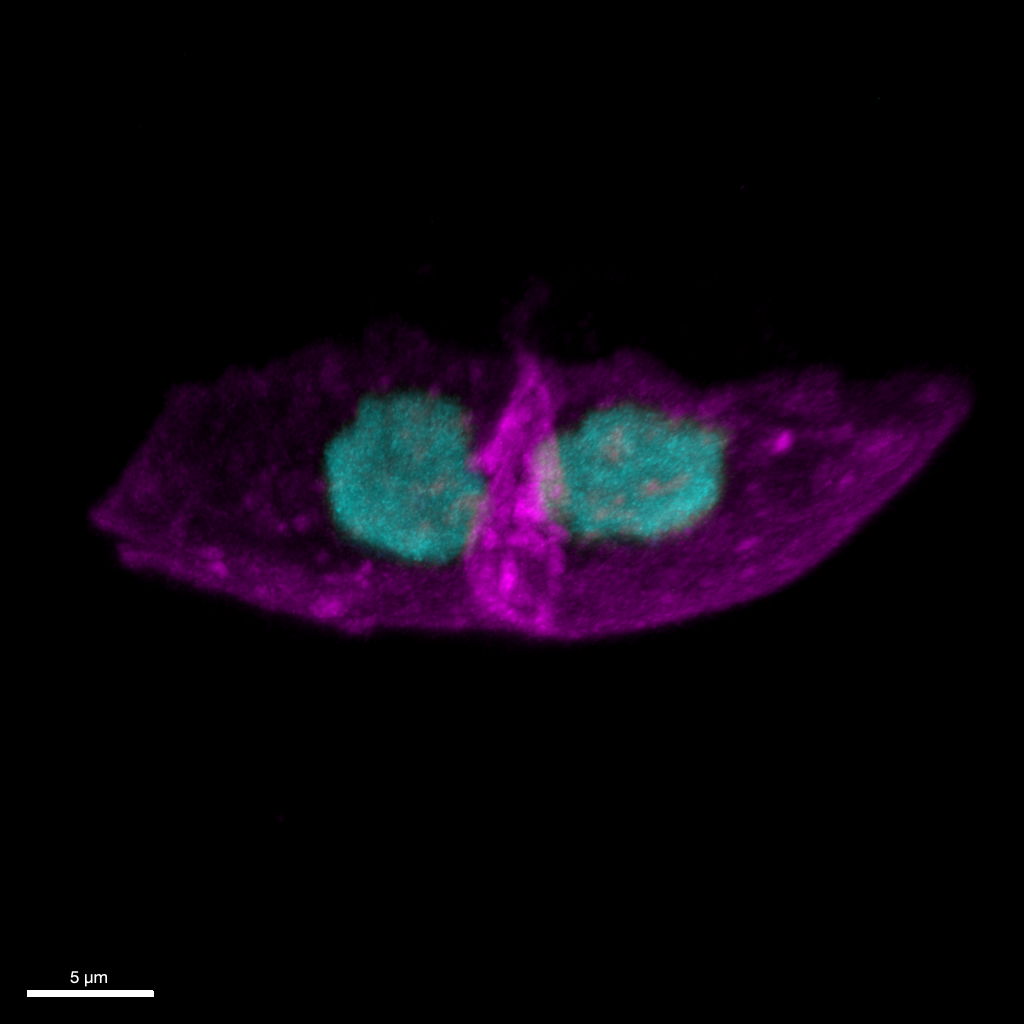

Supplement: Supplementary file 11 — Source data Fig. 1 [file 44318_2025_613_MOESM11_ESM.zip › Figure 1/1B/1B_TVC.tif]

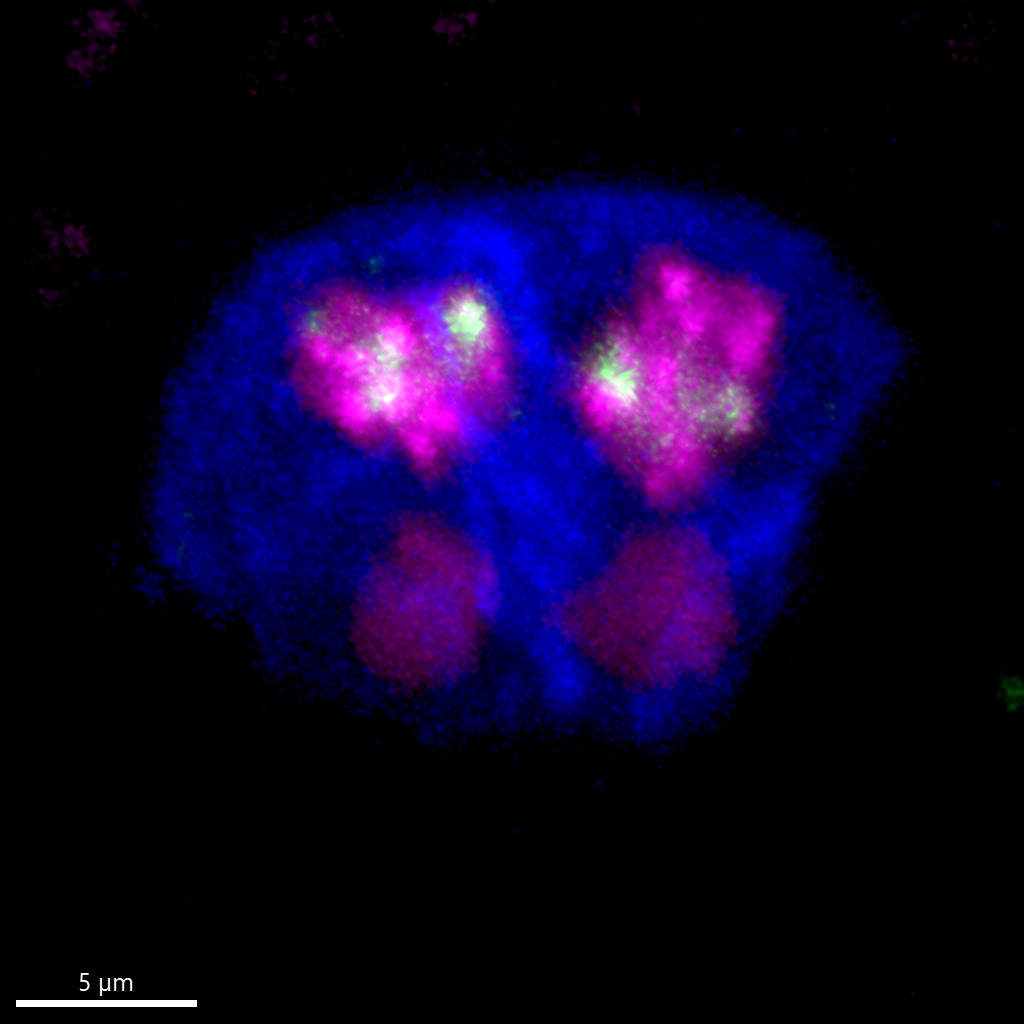

Supplement: Supplementary file 11 — Source data Fig. 1 [file 44318_2025_613_MOESM11_ESM.zip › Figure 1/1D/Figure1D'. 3xHAControl_14.5H(2).tif]

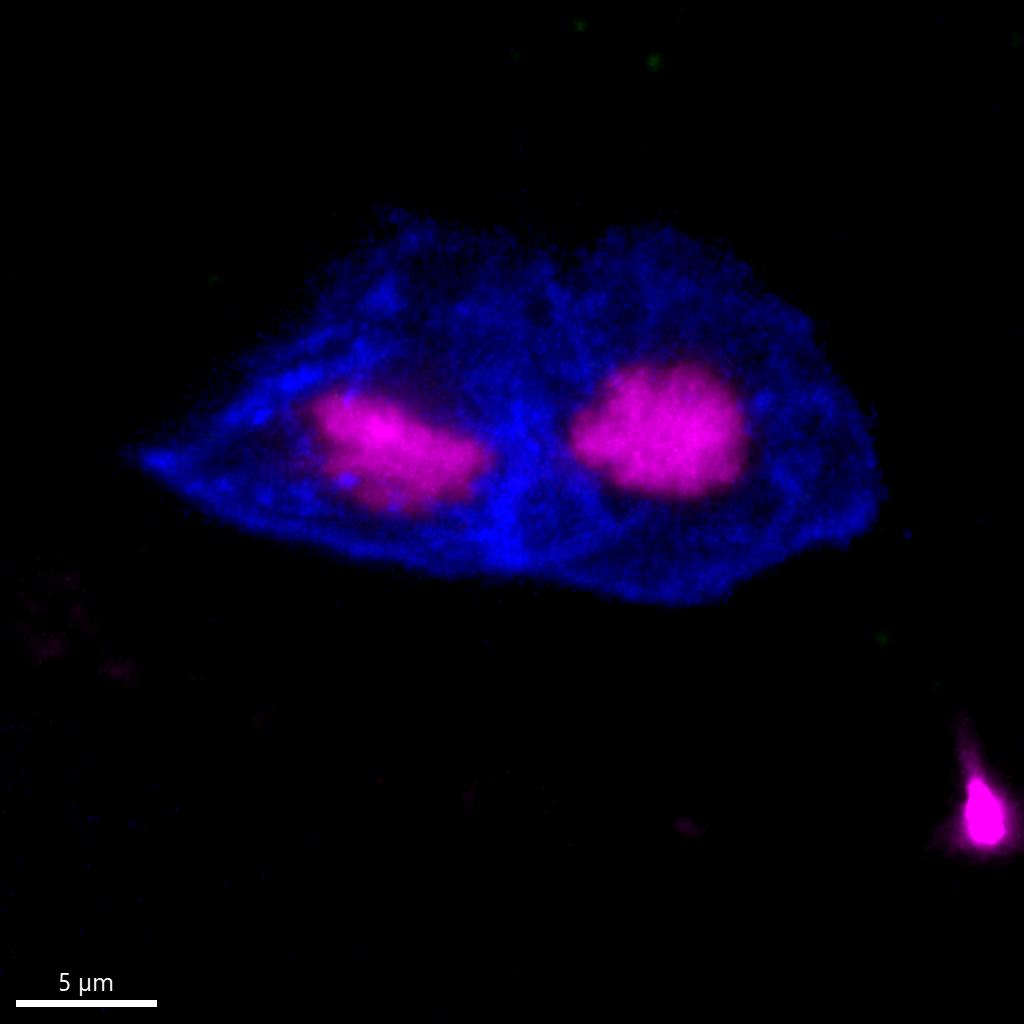

Supplement: Supplementary file 11 — Source data Fig. 1 [file 44318_2025_613_MOESM11_ESM.zip › Figure 1/1D/Figure1D. 3xHAControl_12.5H(1).tif]

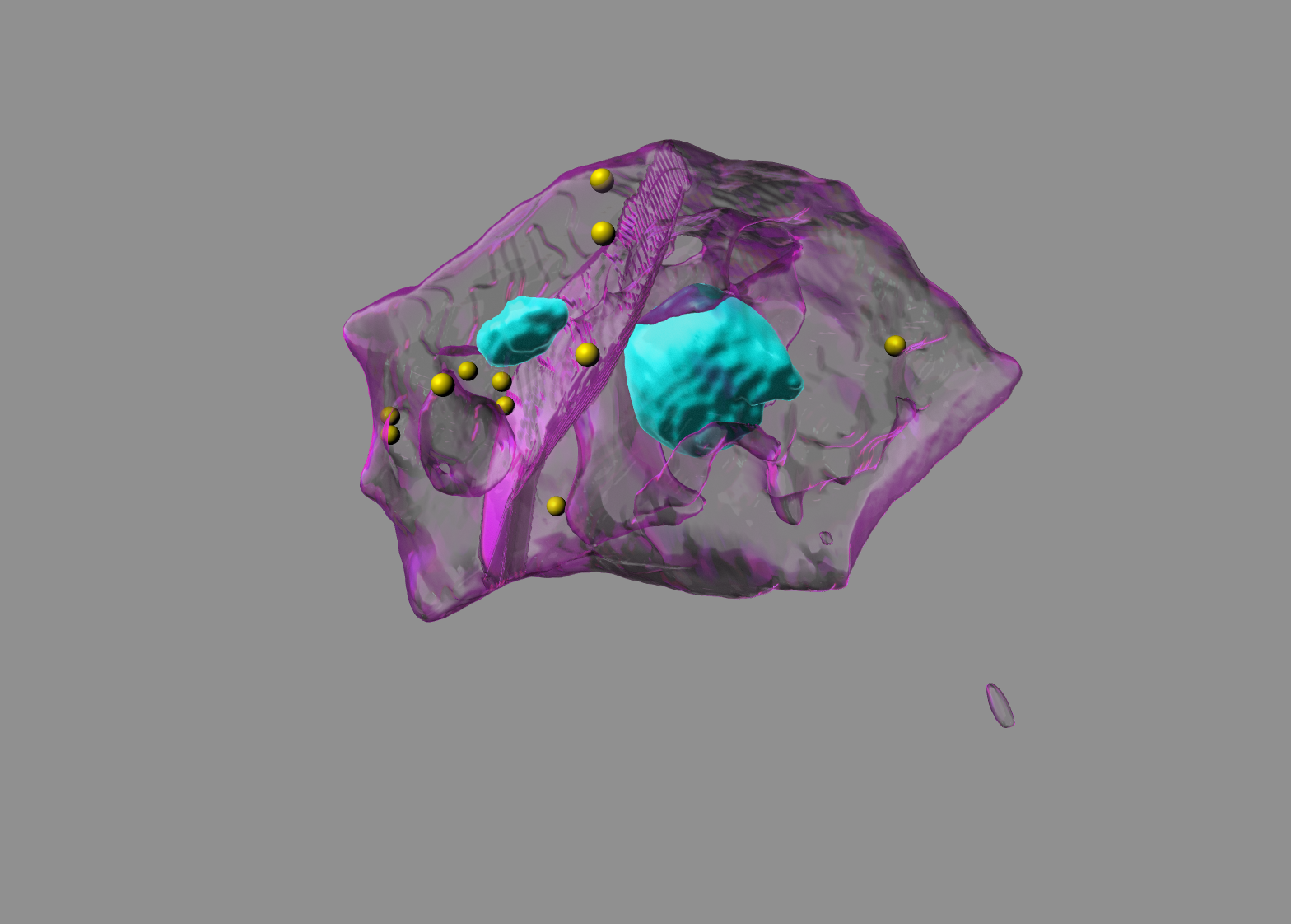

Supplement: Supplementary file 12 — Source data Fig. 3 [file 44318_2025_613_MOESM12_ESM.zip › Figure 3/3I/BMPER_cDNA_Probe_12hpf_d_Series004_2024-03-07T14-28-33.341.tif]

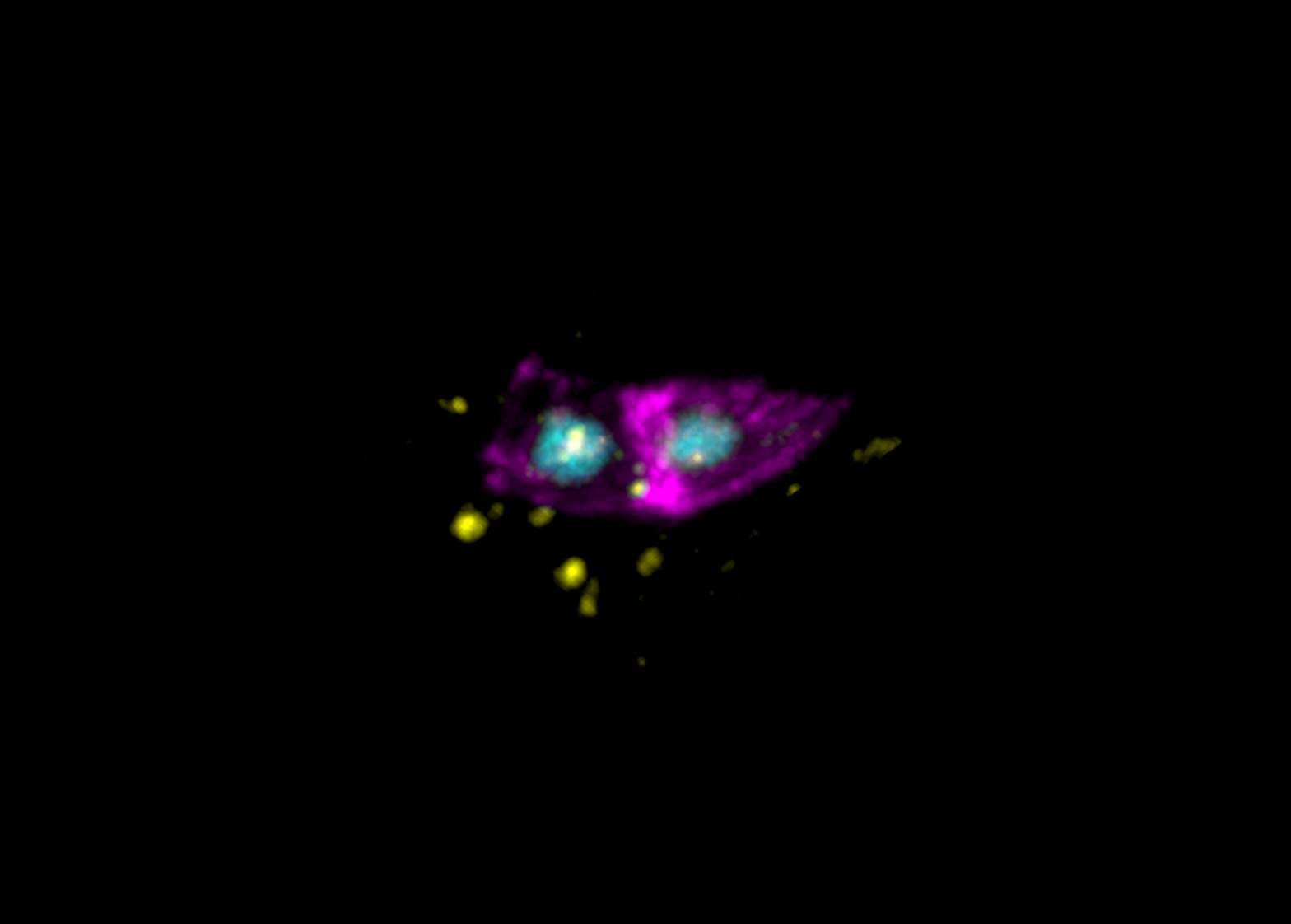

Supplement: Supplementary file 12 — Source data Fig. 3 [file 44318_2025_613_MOESM12_ESM.zip › Figure 3/3I/Ptch_cDNA_Probe_12hpf_h_Series008_2024-03-06T16-38-09.334.tif]

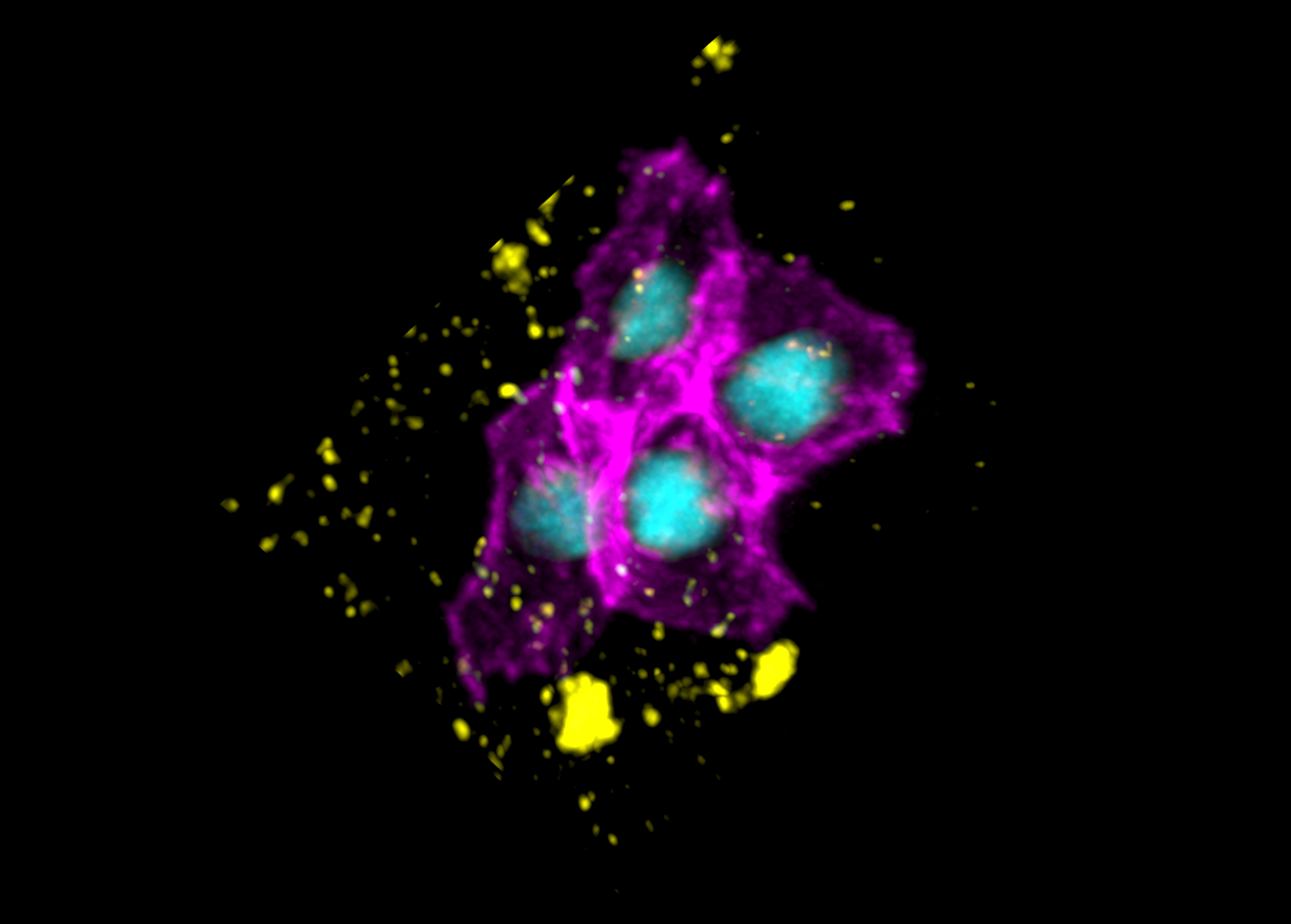

Supplement: Supplementary file 12 — Source data Fig. 3 [file 44318_2025_613_MOESM12_ESM.zip › Figure 3/3I/BMPER_cDNA_Probe_10hpf_a_Series001_2024-03-06T16-58-54.401.tif]

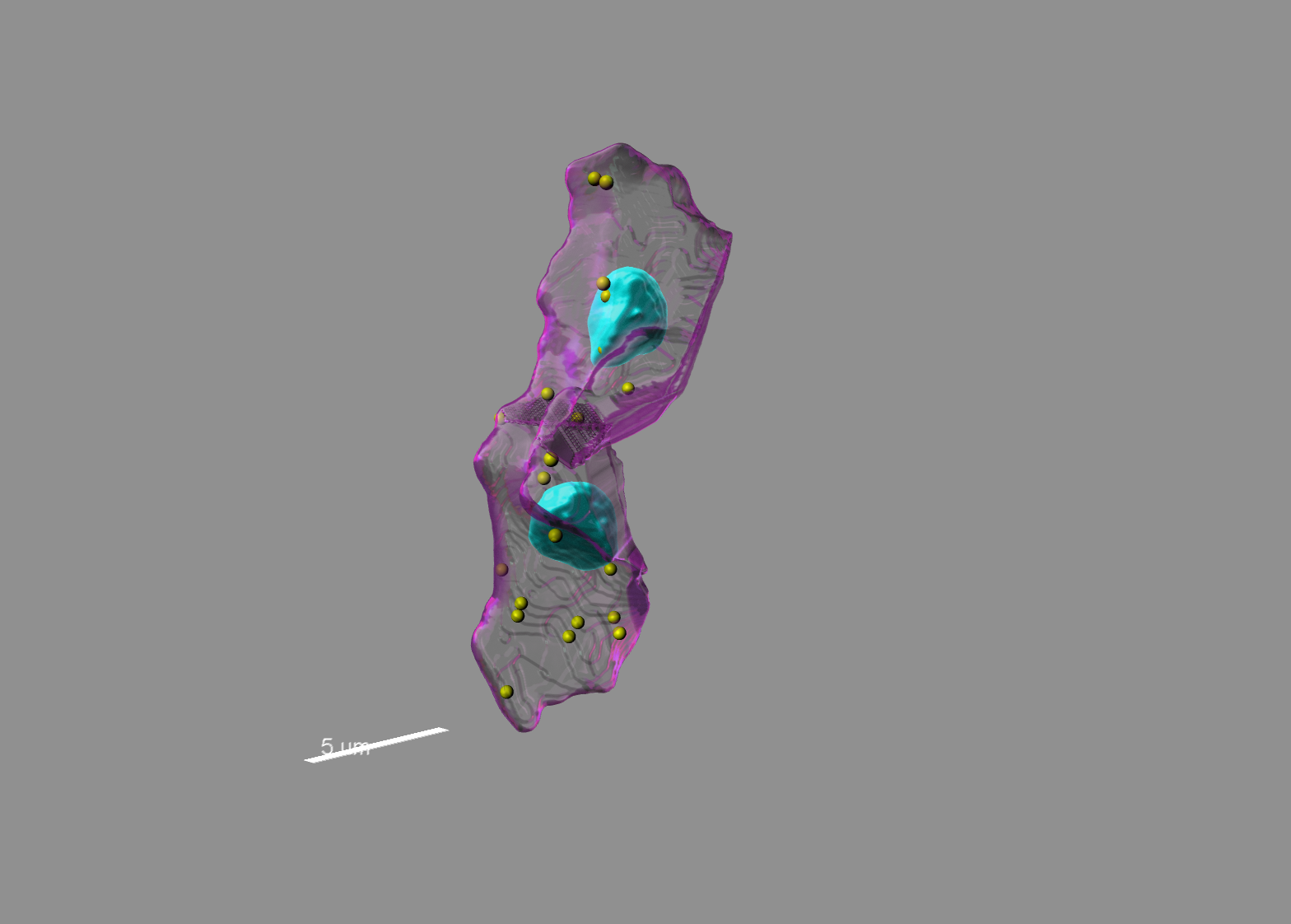

Supplement: Supplementary file 12 — Source data Fig. 3 [file 44318_2025_613_MOESM12_ESM.zip › Figure 3/3I/BMPER_cDNA_Probe_10hpf_a_Series001_2024-03-06T16-59-36.056.tif]

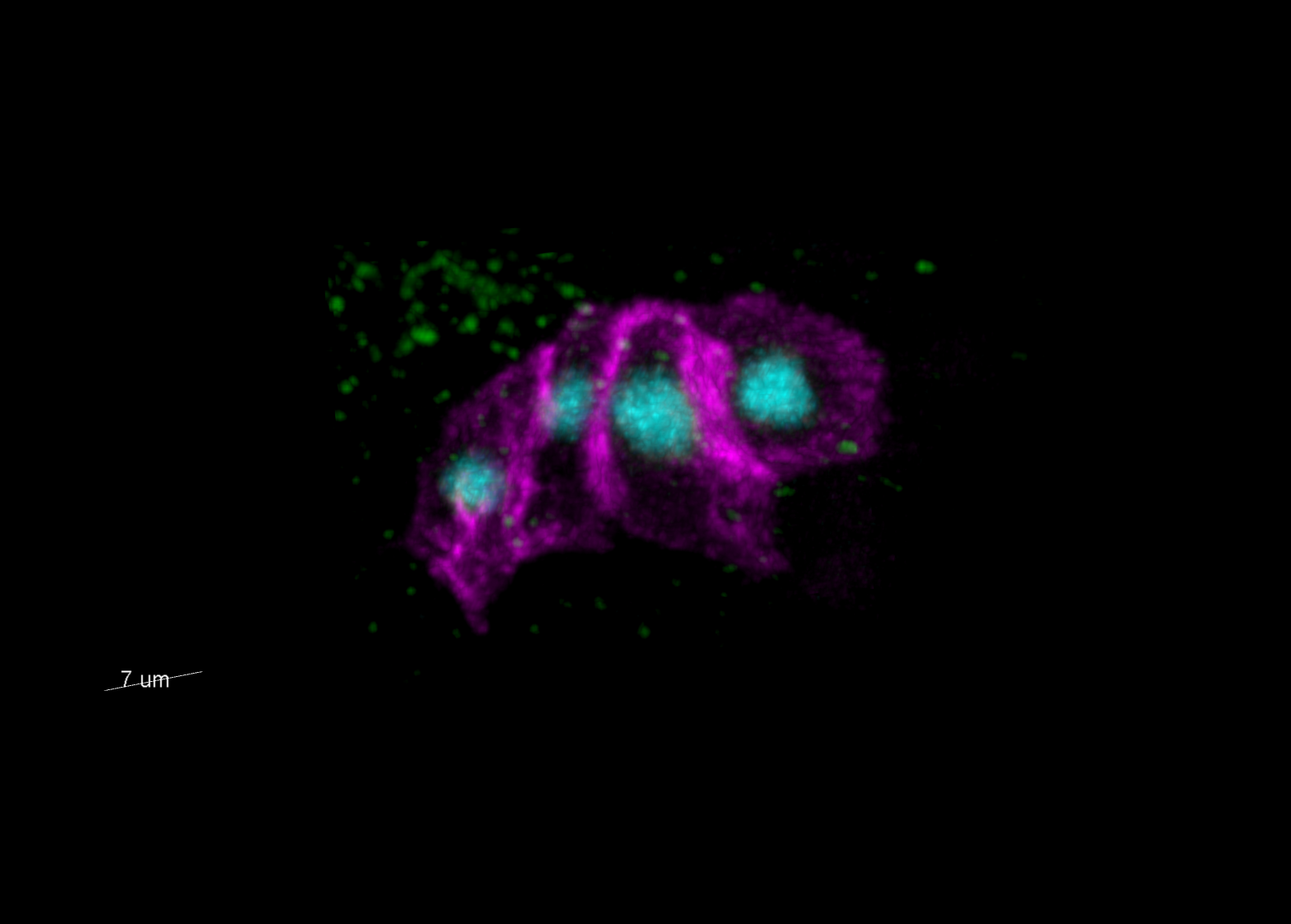

Supplement: Supplementary file 12 — Source data Fig. 3 [file 44318_2025_613_MOESM12_ESM.zip › Figure 3/3I/Ptch_cDNA_Probe_10hpf_d_Series004_2024-03-06T16-23-02.143.tif]

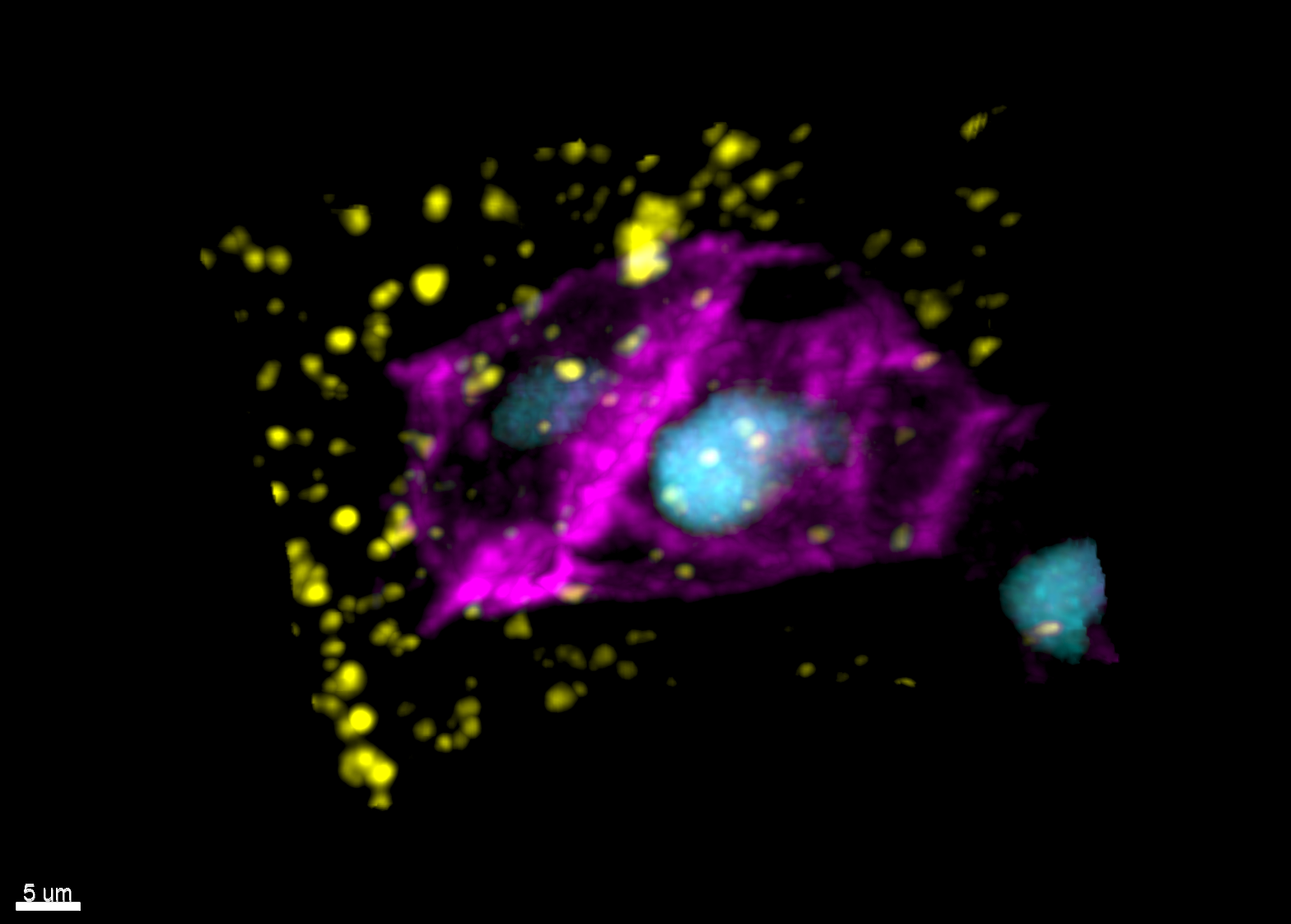

Supplement: Supplementary file 12 — Source data Fig. 3 [file 44318_2025_613_MOESM12_ESM.zip › Figure 3/3I/BMPER_cDNA_Probe_12hpf_d_Series004_2024-03-07T14-28-05.064.tif]

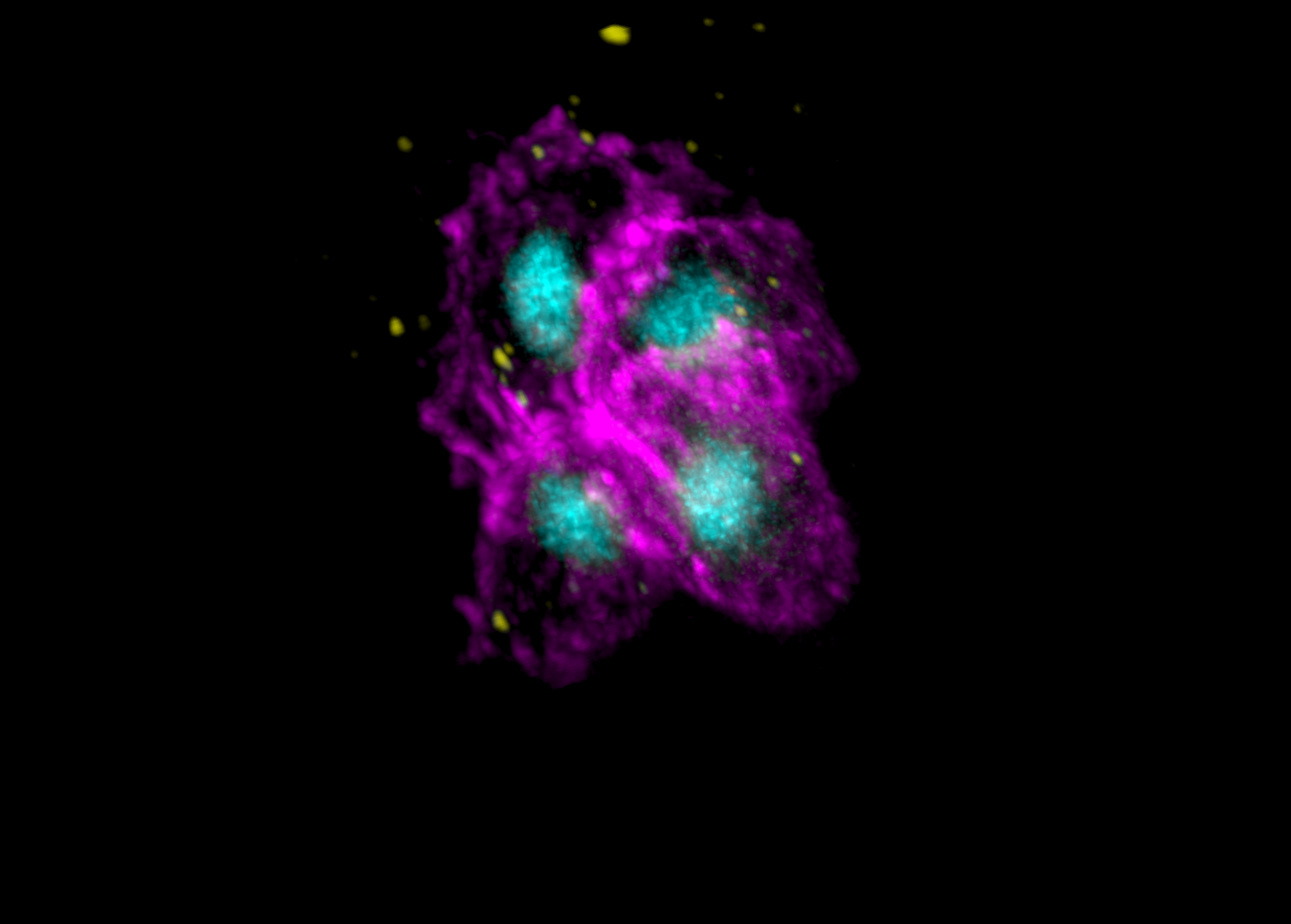

Supplement: Supplementary file 12 — Source data Fig. 3 [file 44318_2025_613_MOESM12_ESM.zip › Figure 3/3I/BMPER_cDNA_Probe_8hpf_a_Series001_2024-03-06T16-50-28.203.tif]

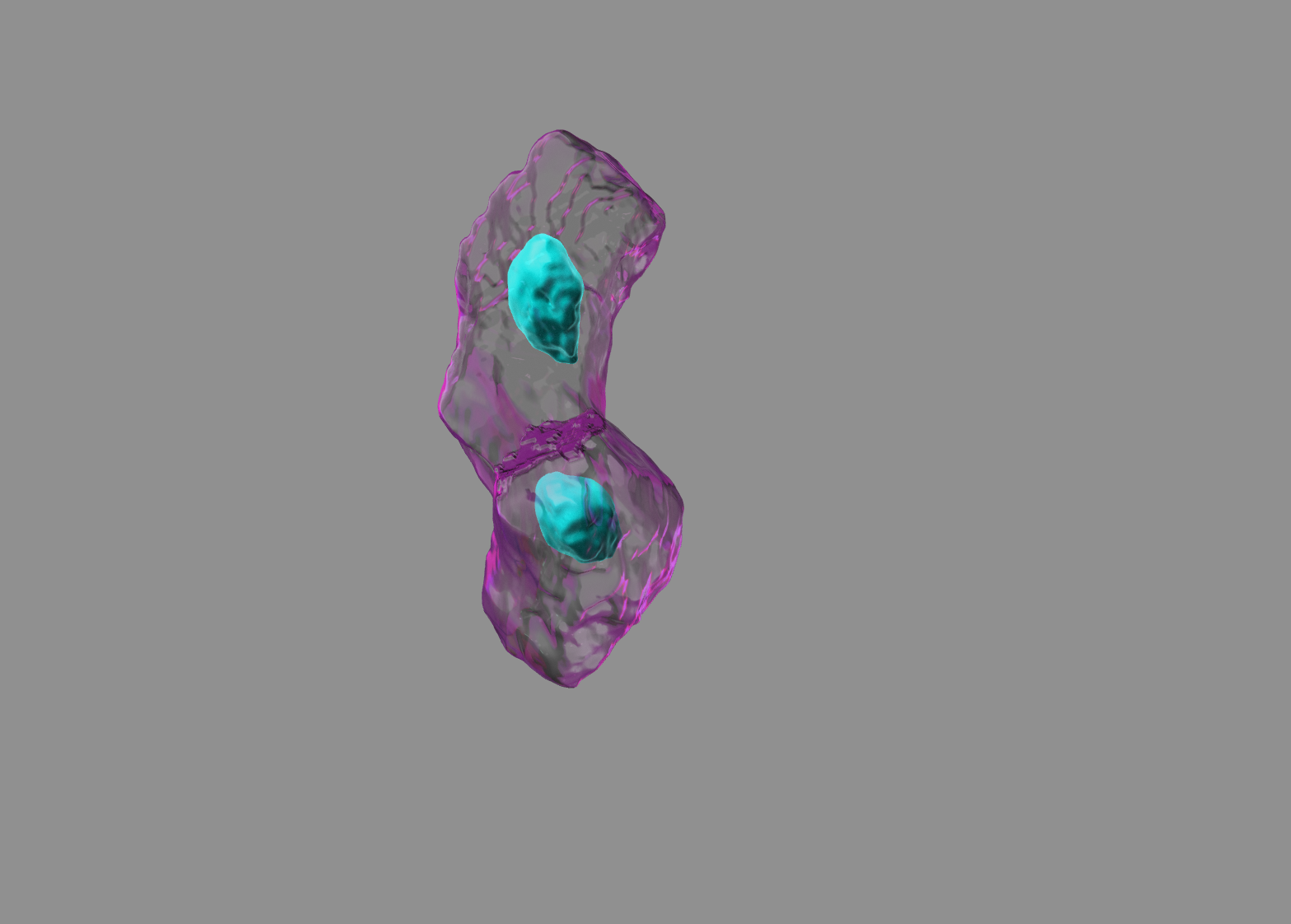

Supplement: Supplementary file 12 — Source data Fig. 3 [file 44318_2025_613_MOESM12_ESM.zip › Figure 3/3I/BMPER_cDNA_Probe_8hpf_a_Series001_2024-03-06T16-51-16.109.tif]

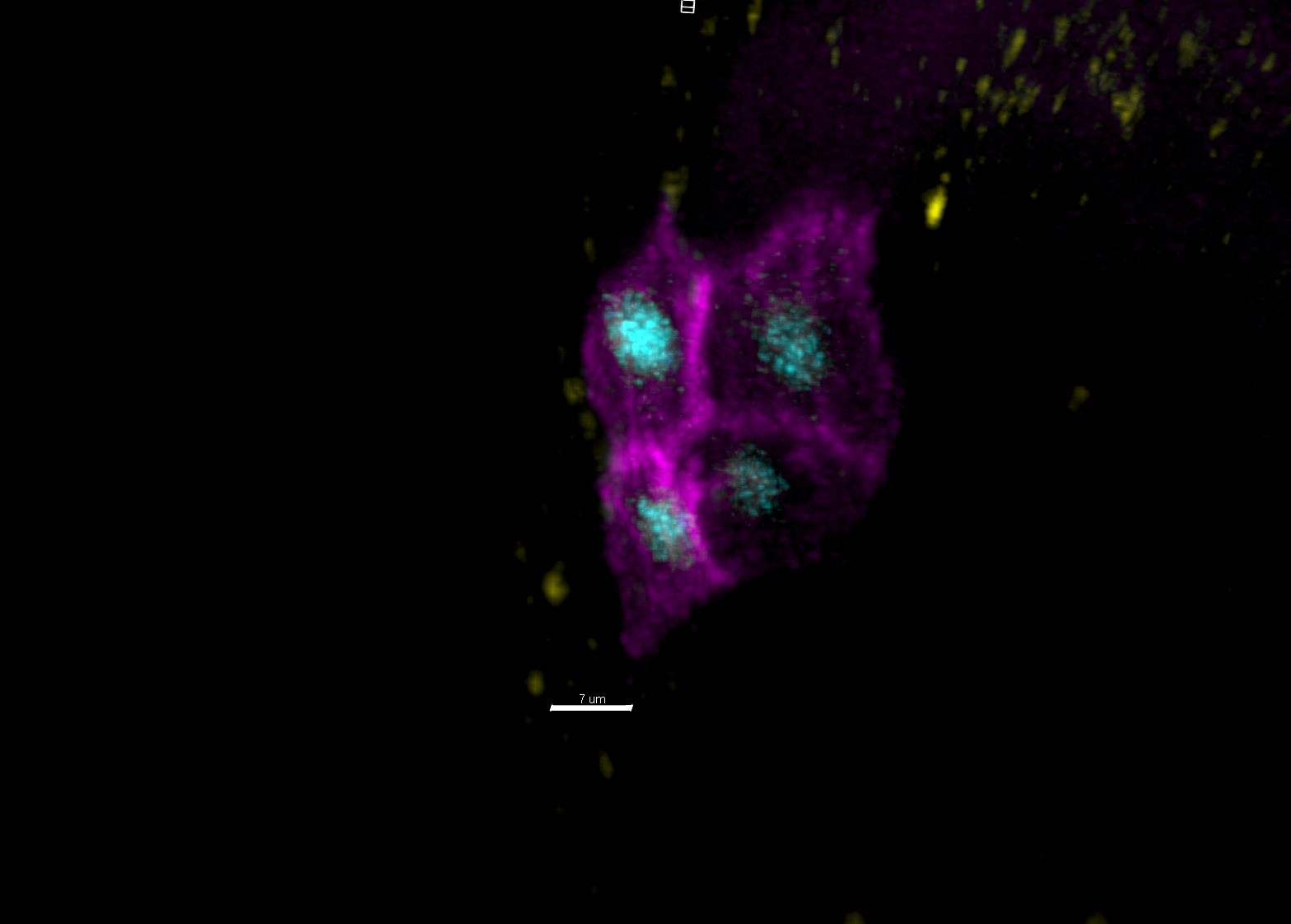

Supplement: Supplementary file 12 — Source data Fig. 3 [file 44318_2025_613_MOESM12_ESM.zip › Figure 3/3I/Ptch_cDNA_Probe_8hpf_h_Series008_2024-03-06T16-14-26.183.tif]

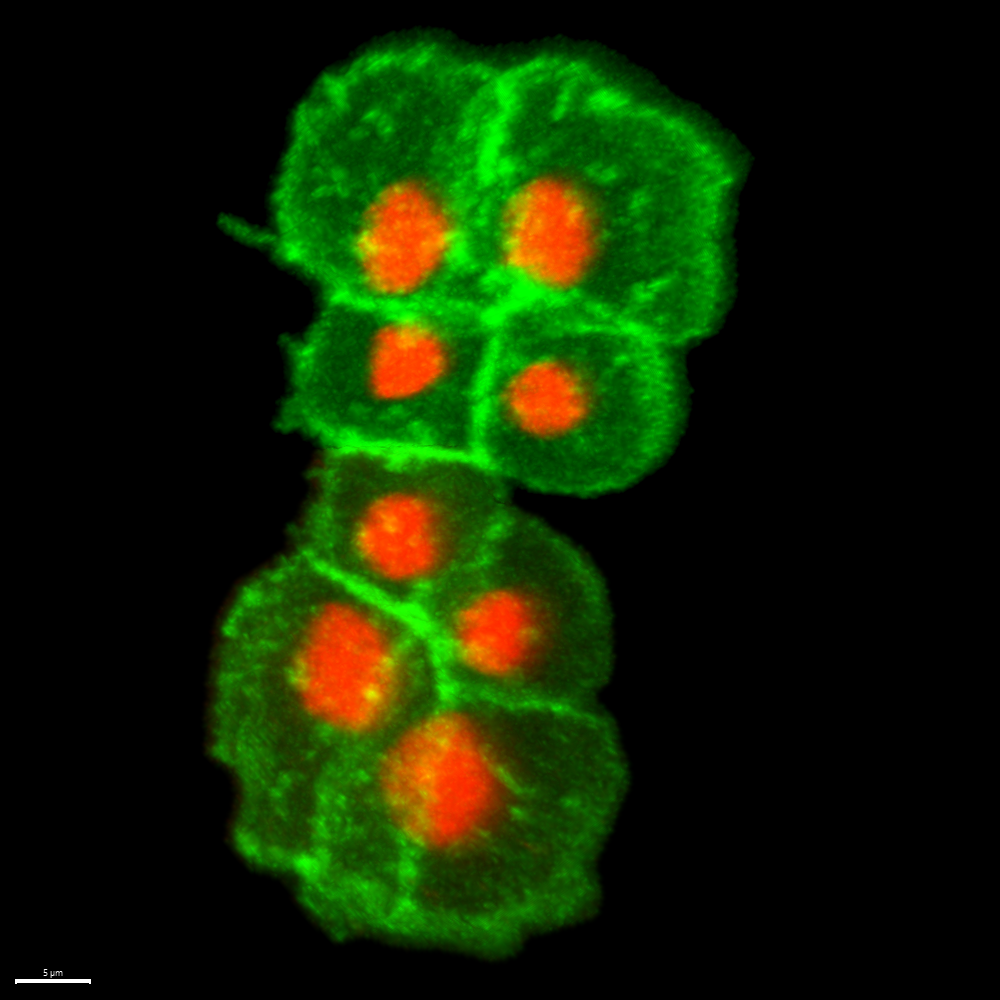

Supplement: Supplementary file 14 — Source data Fig. 6 [file 44318_2025_613_MOESM14_ESM.zip › Figure 6/6B/Tyrosinase slide2 02062020_[ii10_Series011_Image_11]_2024-05-08T09-44-06.711.tif]

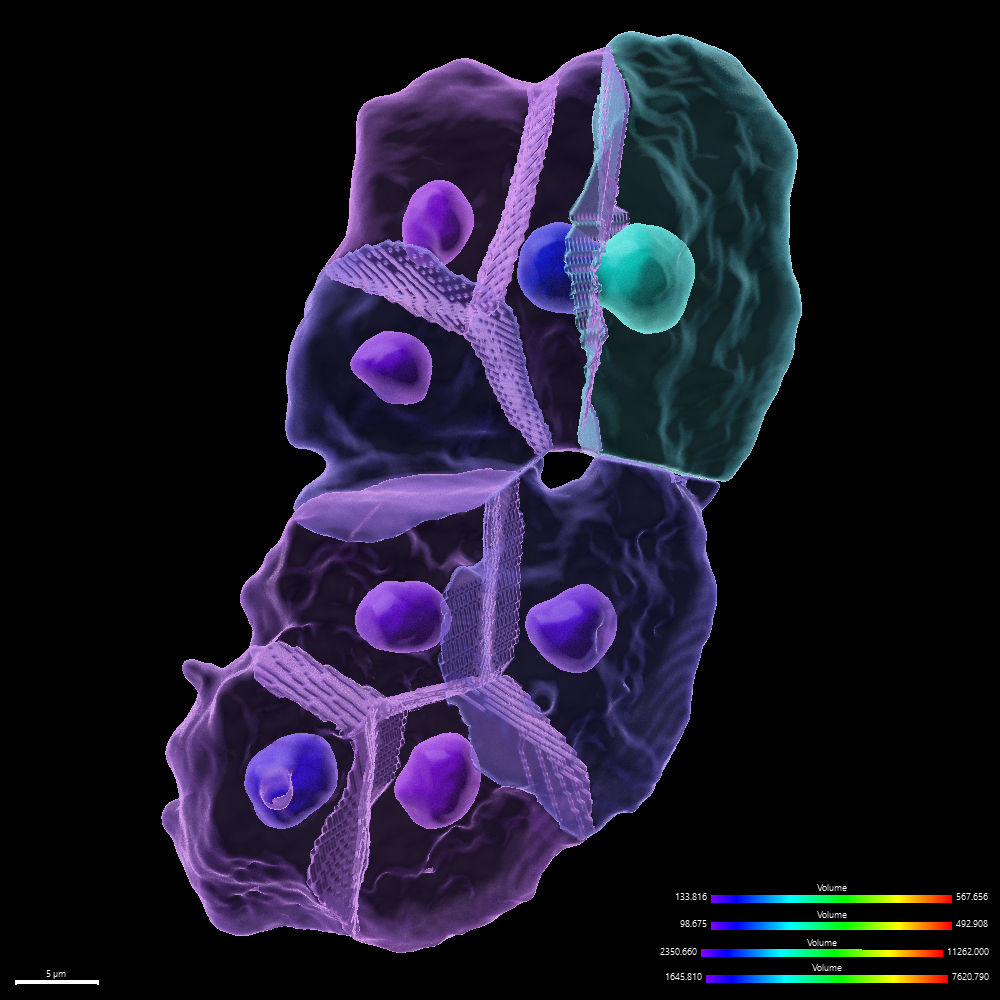

Supplement: Supplementary file 14 — Source data Fig. 6 [file 44318_2025_613_MOESM14_ESM.zip › Figure 6/6B/Depdc slide1 02062020_[ii14_Series015_Image_15]_2024-05-08T09-49-26.745.tif]

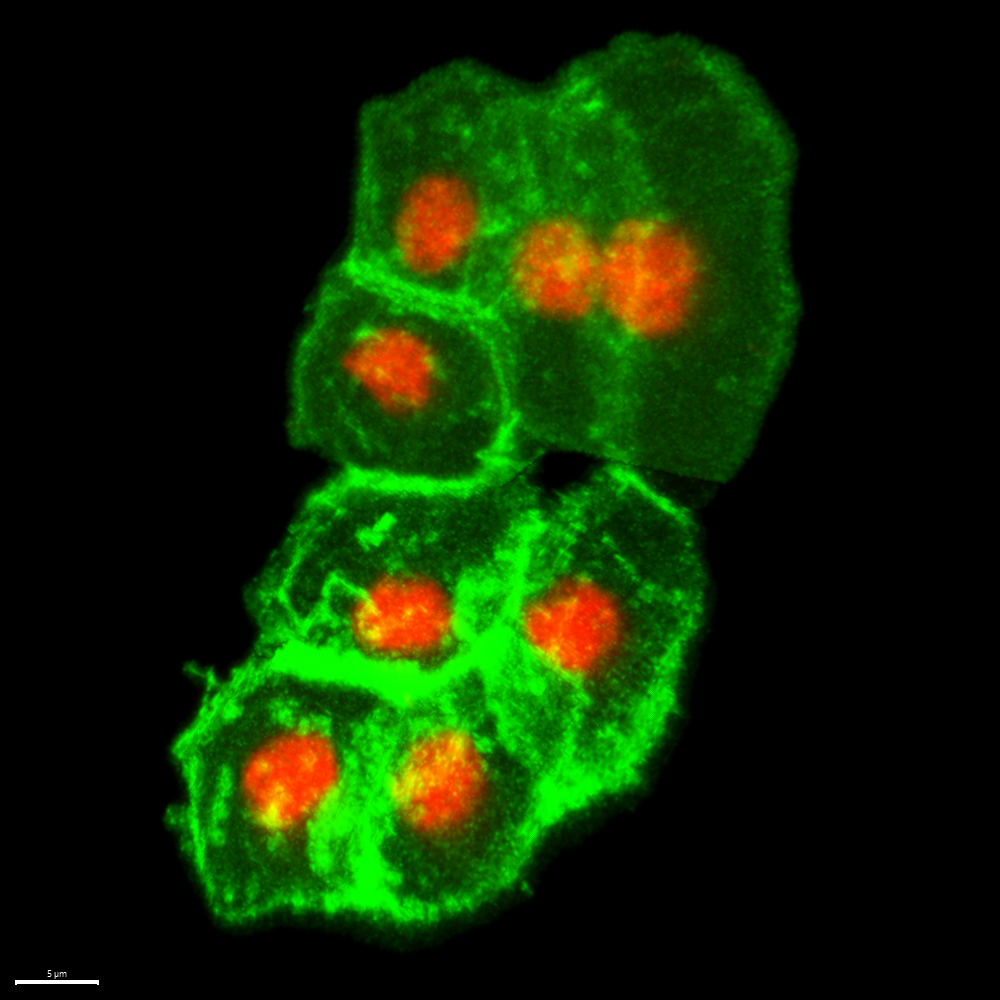

Supplement: Supplementary file 14 — Source data Fig. 6 [file 44318_2025_613_MOESM14_ESM.zip › Figure 6/6B/Depdc slide1 02062020_[ii14_Series015_Image_15]_2024-05-08T09-49-38.698.tif]

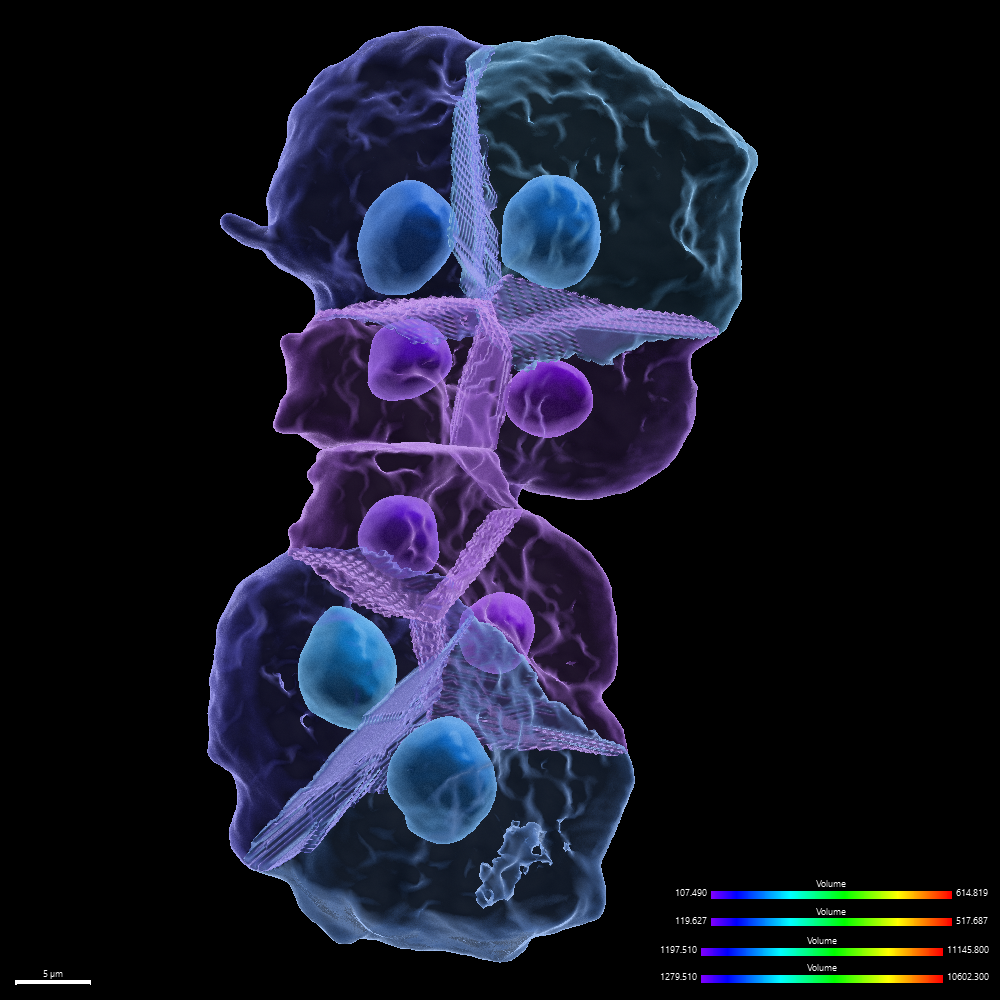

Supplement: Supplementary file 14 — Source data Fig. 6 [file 44318_2025_613_MOESM14_ESM.zip › Figure 6/6B/Tyrosinase slide2 02062020_[ii10_Series011_Image_11]_2024-05-08T09-44-17.694.tif]

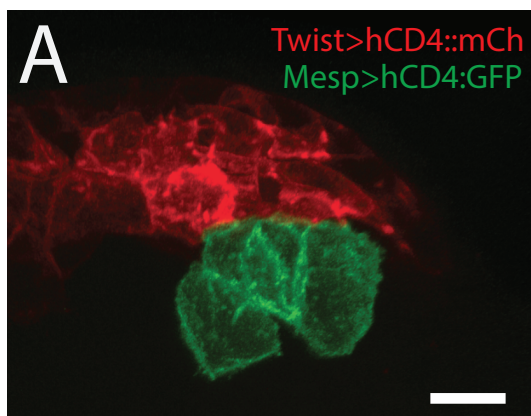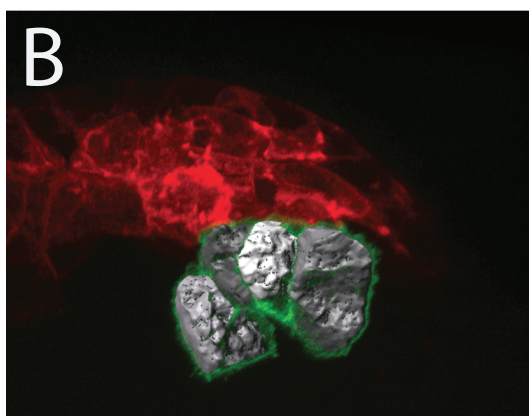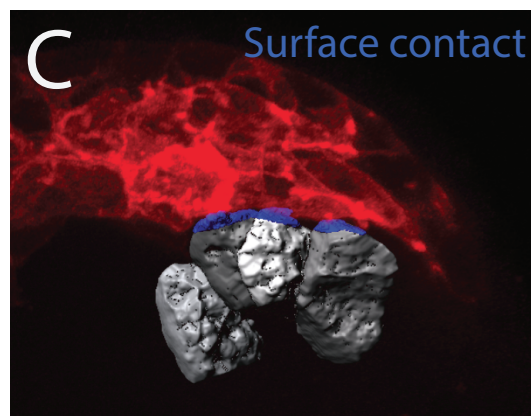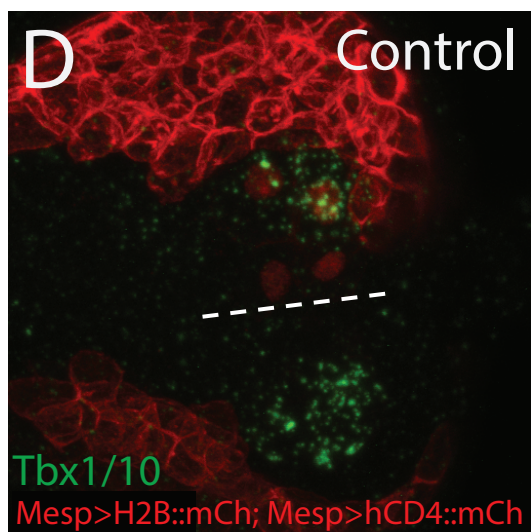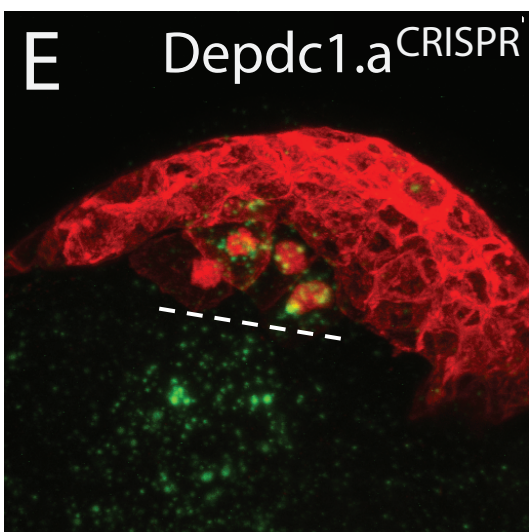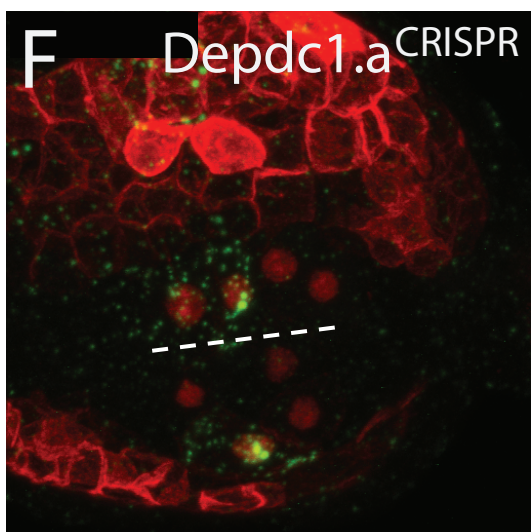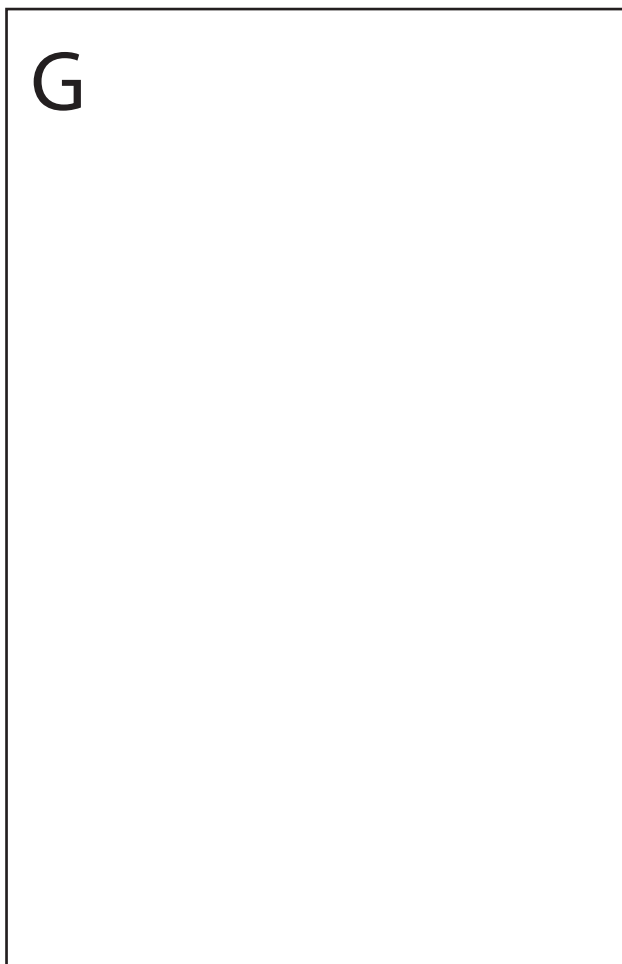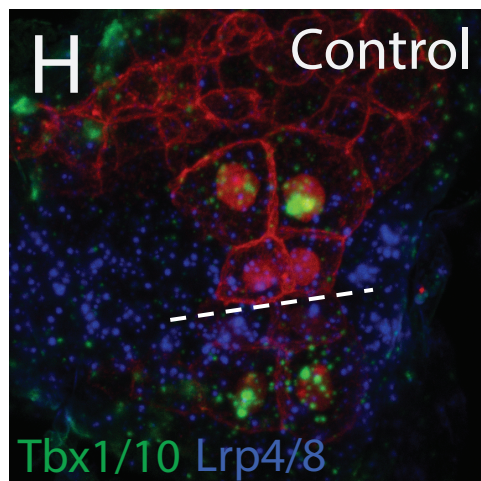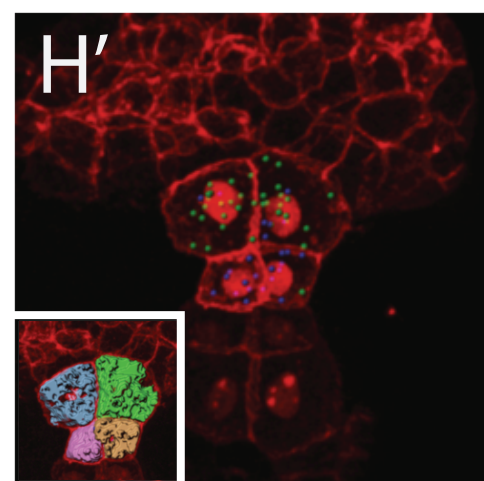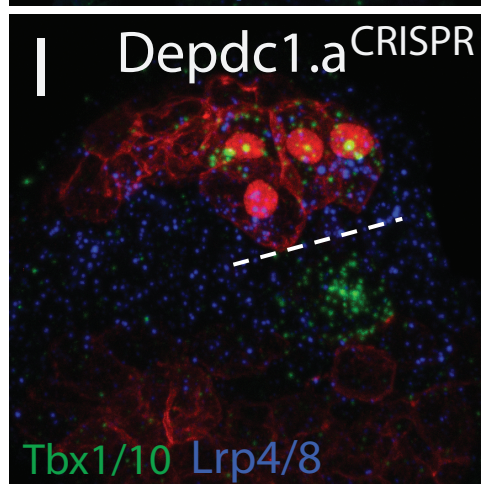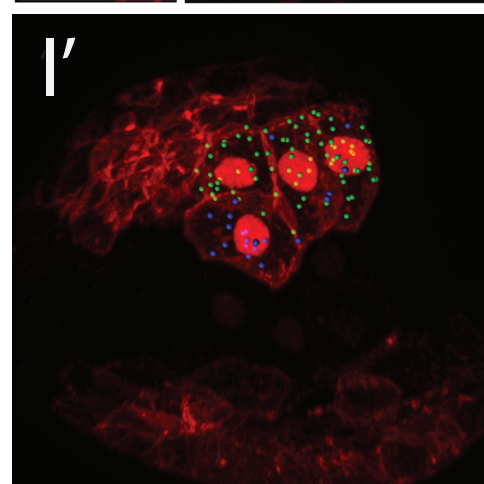

Supplement: Supplementary file 14 — Source data Fig. 6 [file 44318_2025_613_MOESM14_ESM.zip › Figure 6/6C/niche-Fig3.pdf]

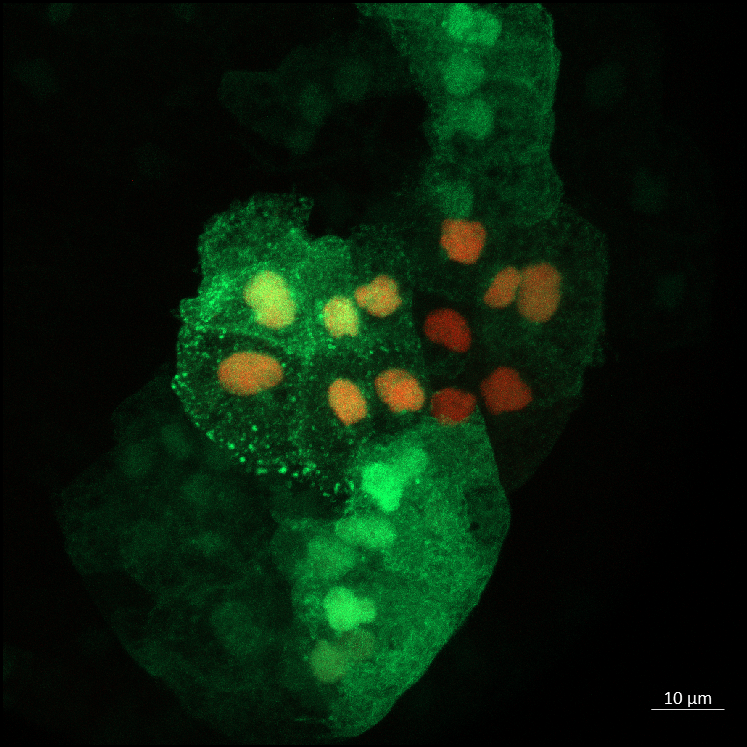

Supplement: Supplementary file 15 — Source data Fig. 8 [file 44318_2025_613_MOESM15_ESM.zip › Figure 8/Figure 8O/GATA.tif]

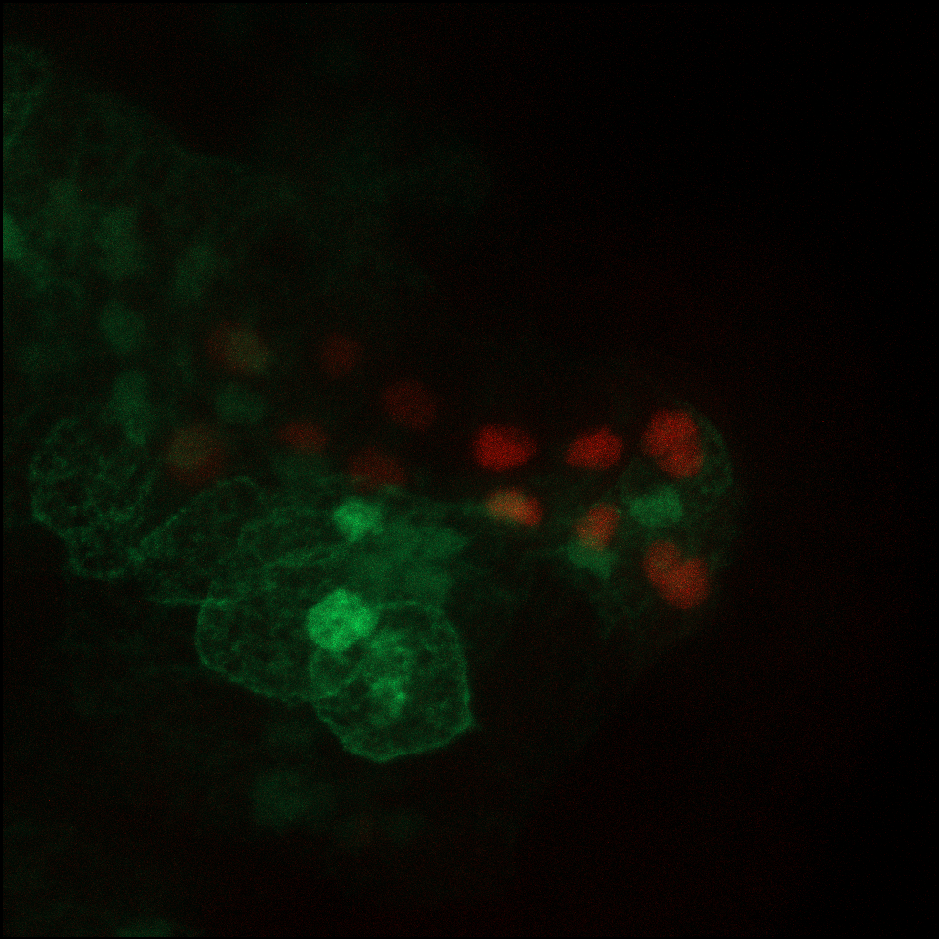

Supplement: Supplementary file 15 — Source data Fig. 8 [file 44318_2025_613_MOESM15_ESM.zip › Figure 8/Figure 8O/Flank+GATA.tif]

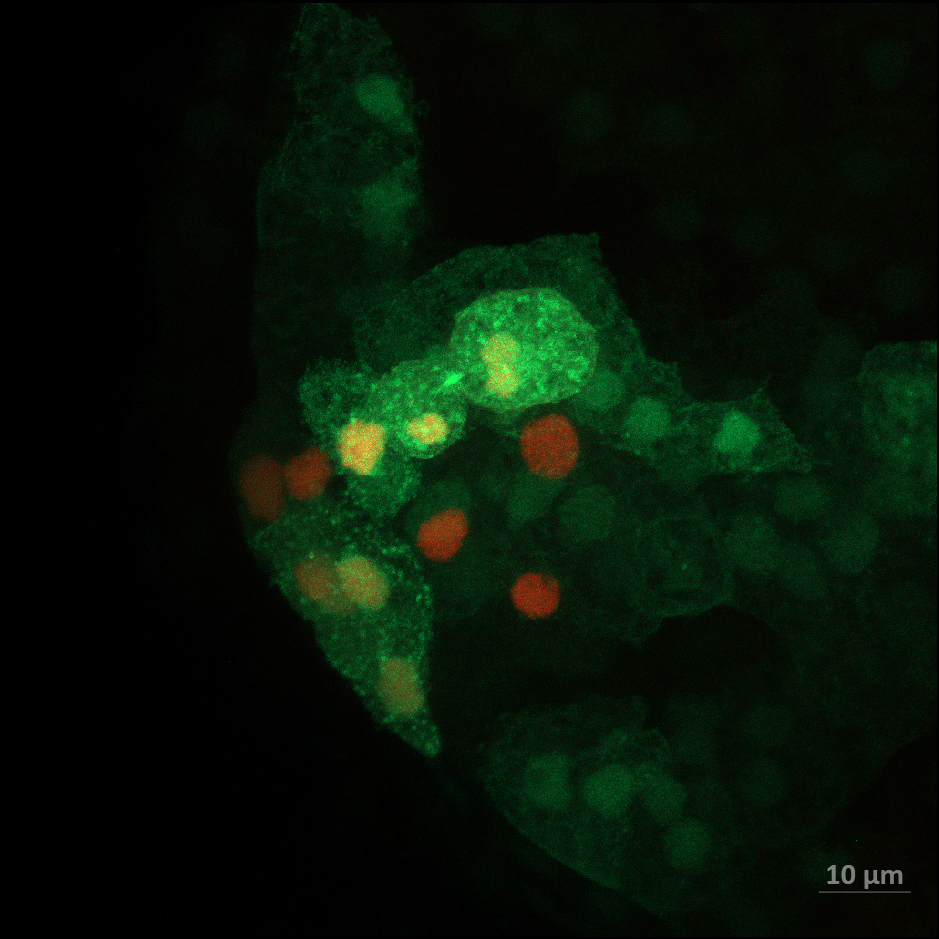

Supplement: Supplementary file 15 — Source data Fig. 8 [file 44318_2025_613_MOESM15_ESM.zip › Figure 8/Figure 8O/FOX.tif]

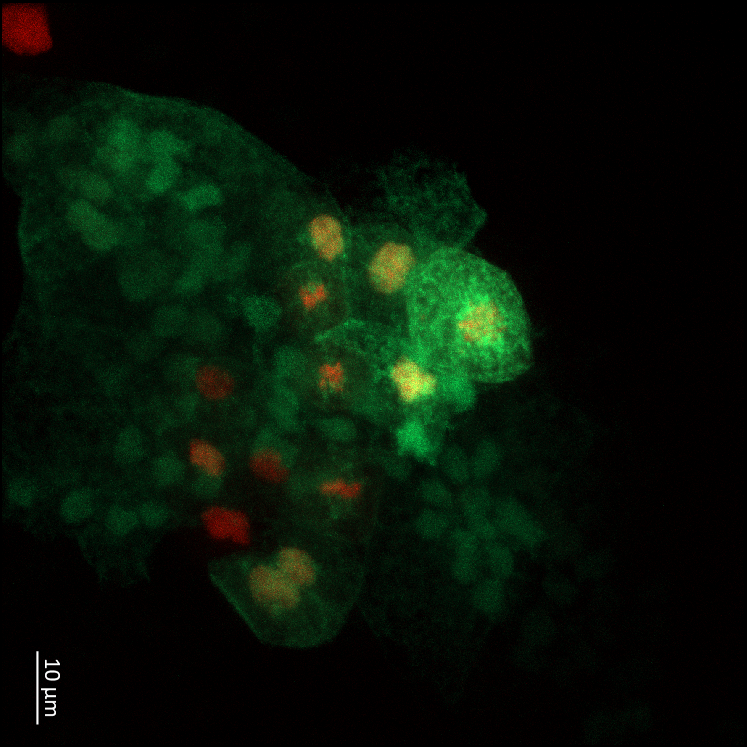

Supplement: Supplementary file 15 — Source data Fig. 8 [file 44318_2025_613_MOESM15_ESM.zip › Figure 8/Figure 8O/Flank.tif]

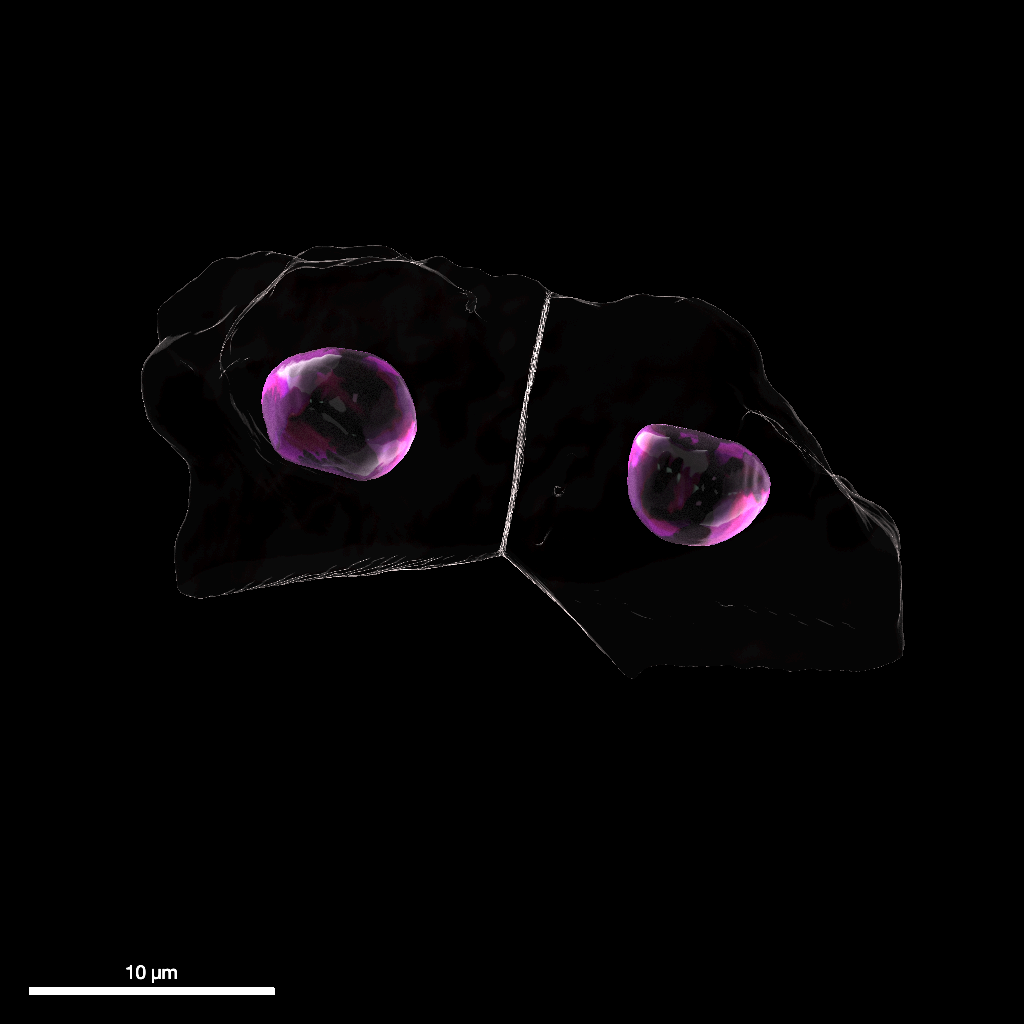

Supplement: Supplementary file 15 — Source data Fig. 8 [file 44318_2025_613_MOESM15_ESM.zip › Figure 8/8A/20230317 DepdcGata_dFISH_8H_[ii2_DepdcGata_dFISH_8H_11_Image_3]_2024-03-22T06-39-15.390 copy.tif]

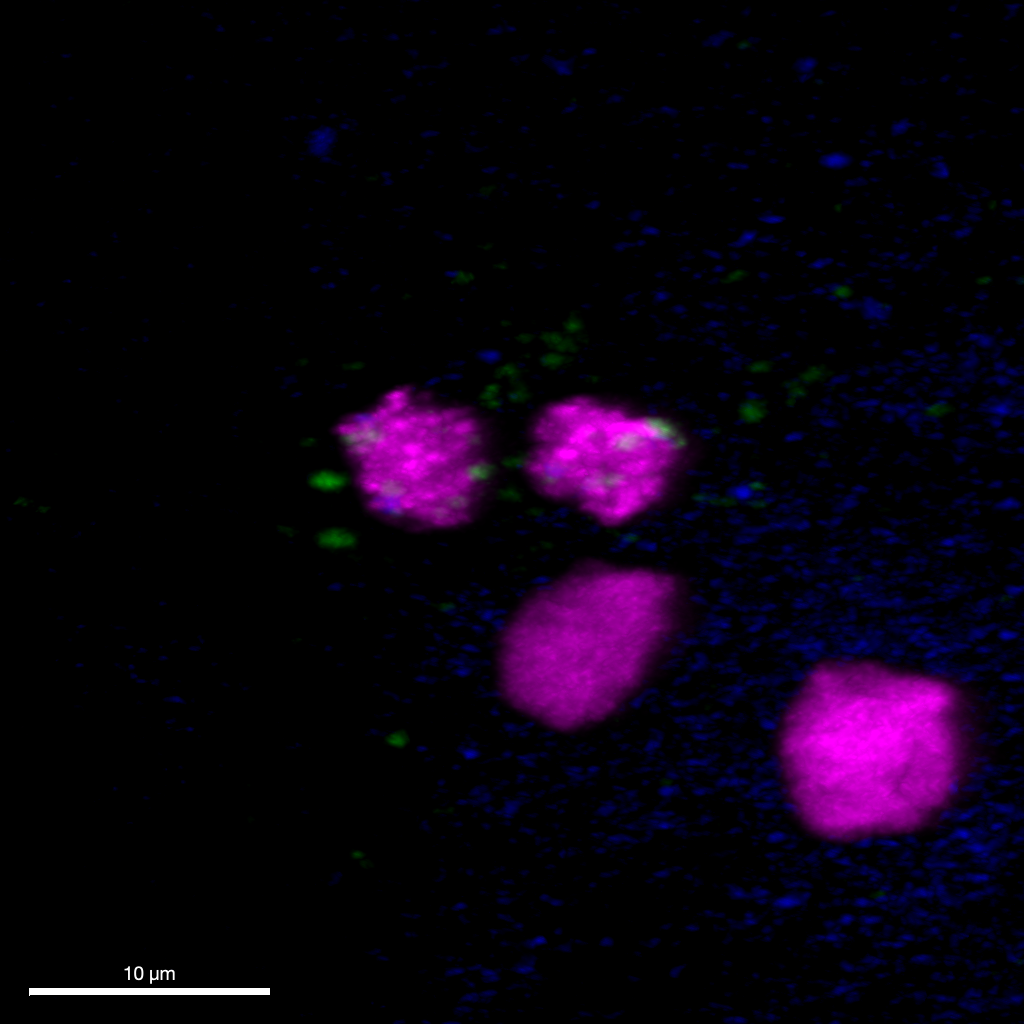

Supplement: Supplementary file 15 — Source data Fig. 8 [file 44318_2025_613_MOESM15_ESM.zip › Figure 8/8A/20230316 DepdcGata_dFISH_10H_[ii8_DepdcGata_dFISH_10H_14-1024_Image_9]_2024-03-22T06-32-25.024.tif]

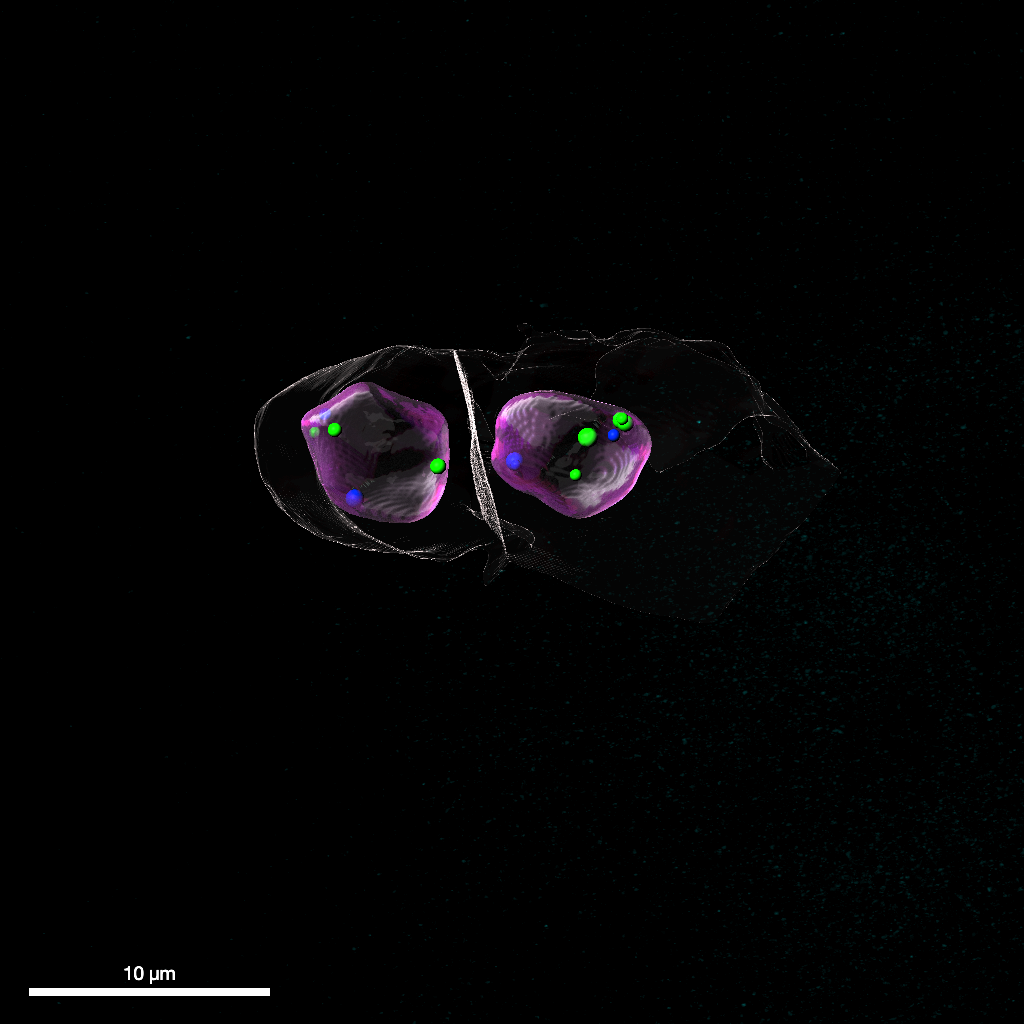

Supplement: Supplementary file 15 — Source data Fig. 8 [file 44318_2025_613_MOESM15_ESM.zip › Figure 8/8A/20230316 DepdcGata_dFISH_10H_[ii8_DepdcGata_dFISH_10H_14-1024_Image_9]_2024-03-22T06-27-46.121 copy.tif]

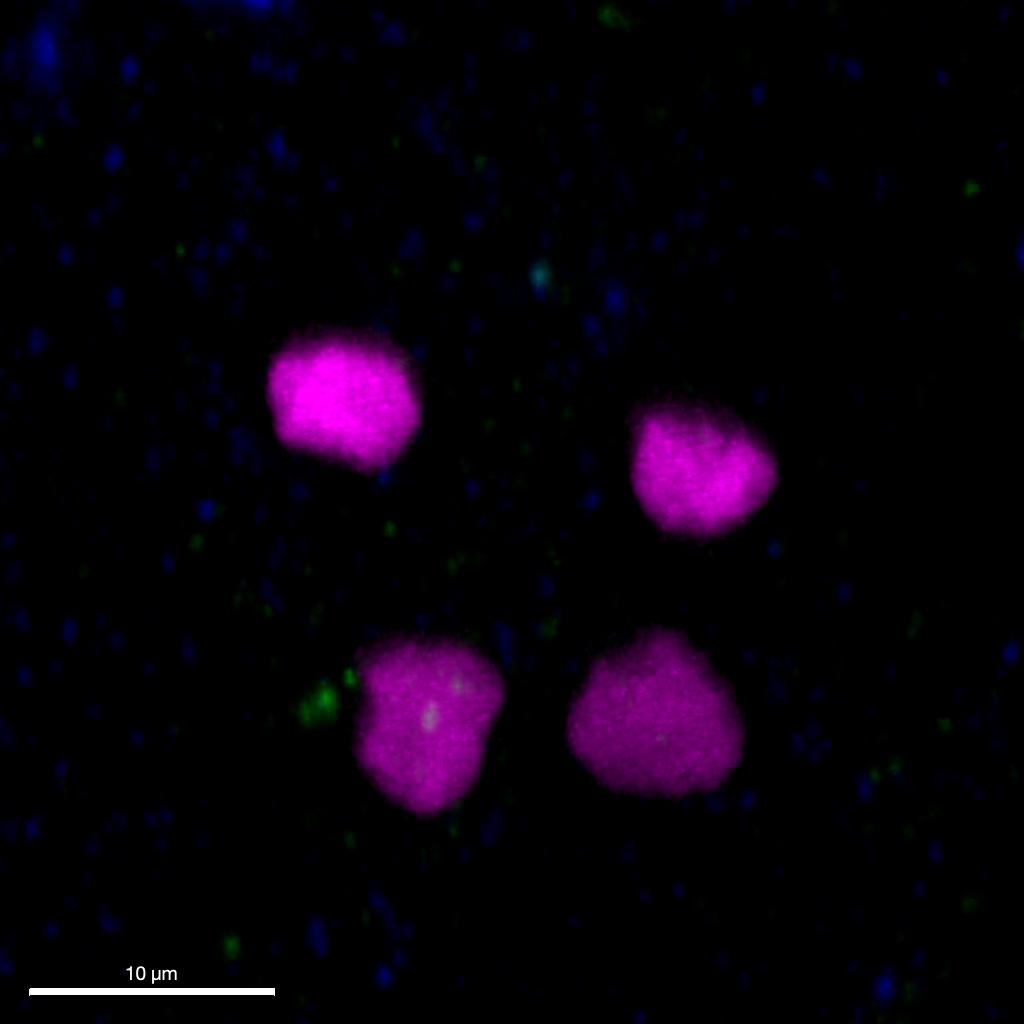

Supplement: Supplementary file 15 — Source data Fig. 8 [file 44318_2025_613_MOESM15_ESM.zip › Figure 8/8A/20230317 DepdcGata_dFISH_8H_[ii2_DepdcGata_dFISH_8H_11_Image_3]_2024-03-22T06-23-51.017.tif]

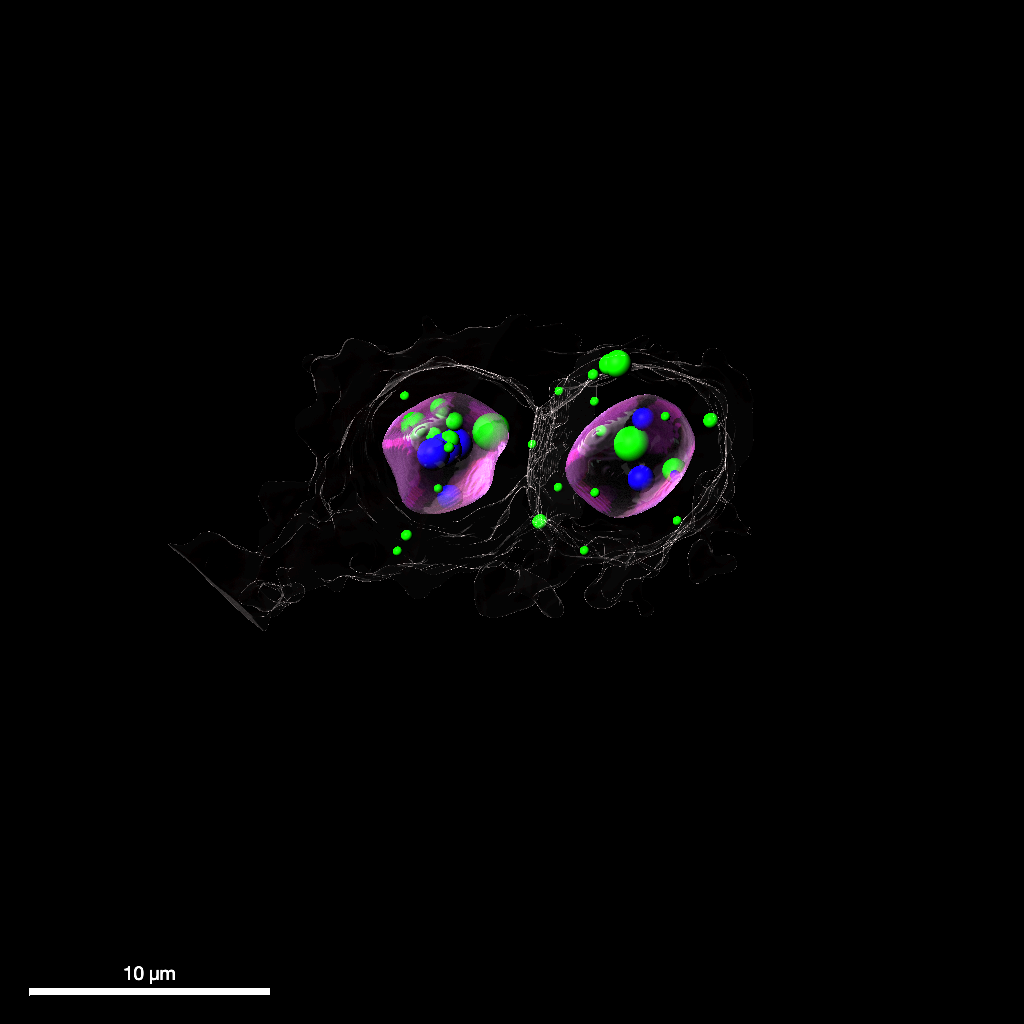

Supplement: Supplementary file 15 — Source data Fig. 8 [file 44318_2025_613_MOESM15_ESM.zip › Figure 8/8A/20230308 DepdcGata_dFISH_12H_[ii4_DepdcGata_dFISH_12H_9-1024_Image_5]_2024-03-22T06-37-36.709 copy.tif]

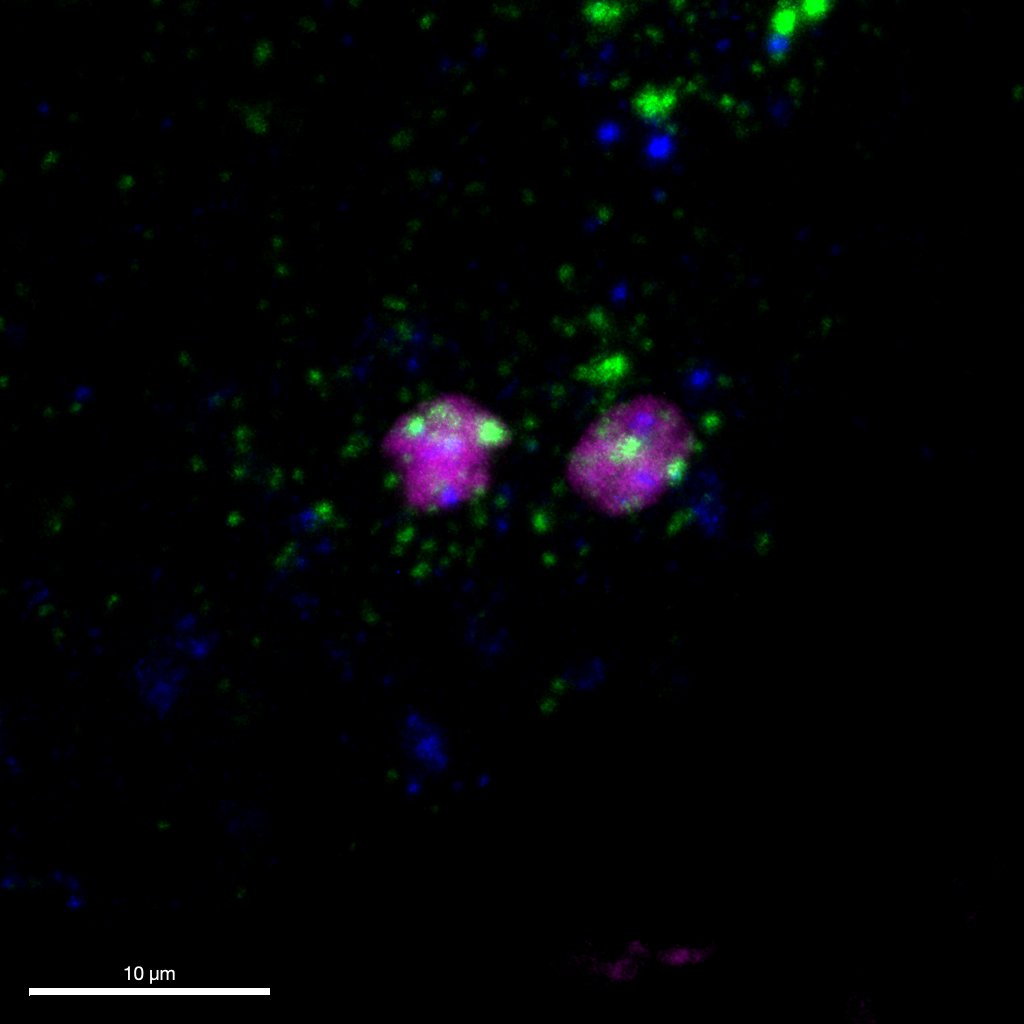

Supplement: Supplementary file 15 — Source data Fig. 8 [file 44318_2025_613_MOESM15_ESM.zip › Figure 8/8A/20230308 DepdcGata_dFISH_12H_[ii4_DepdcGata_dFISH_12H_9-1024_Image_5]_2024-03-22T06-37-03.834.tif]

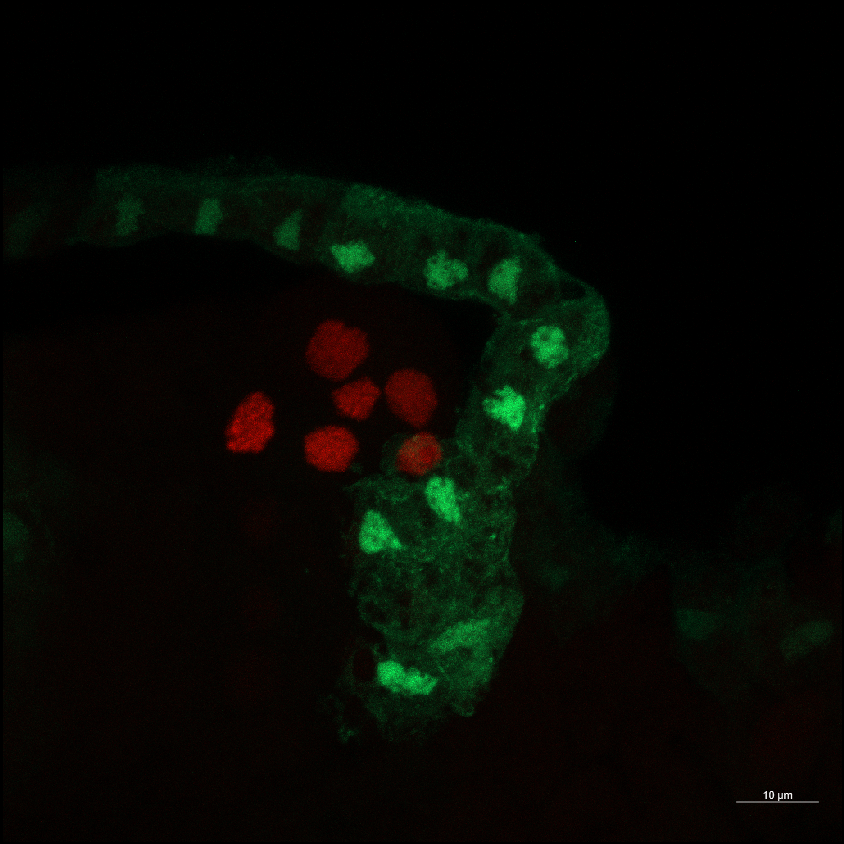

Supplement: Supplementary file 15 — Source data Fig. 8 [file 44318_2025_613_MOESM15_ESM.zip › Figure 8/Figure 8K/-1969_-1.tif]

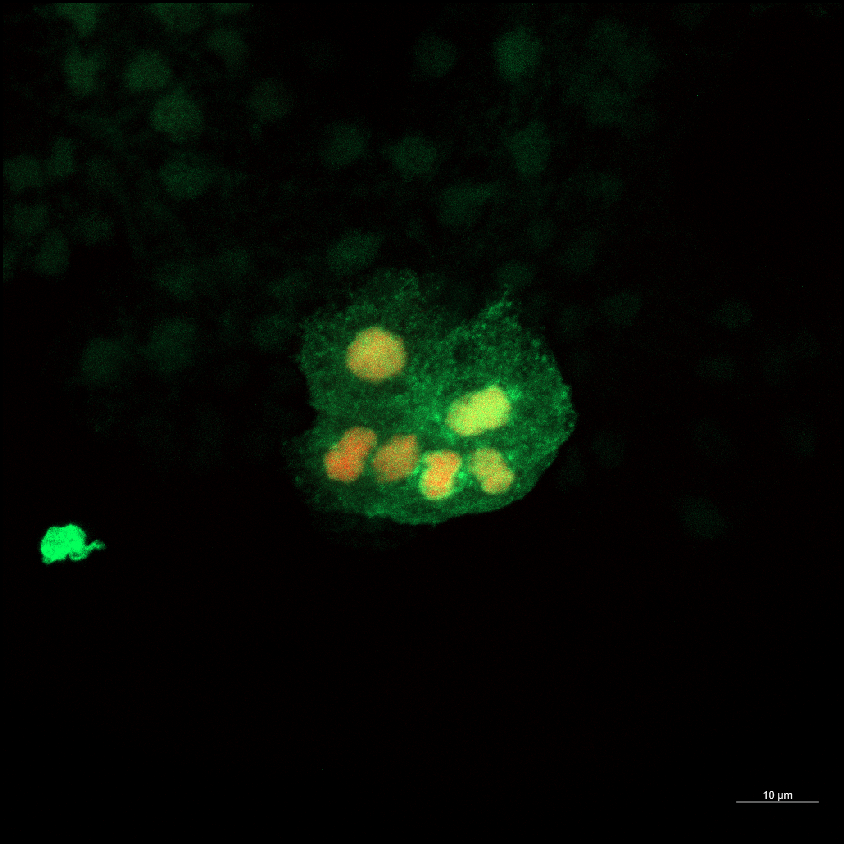

Supplement: Supplementary file 15 — Source data Fig. 8 [file 44318_2025_613_MOESM15_ESM.zip › Figure 8/Figure 8K/-2537_-1.tif]

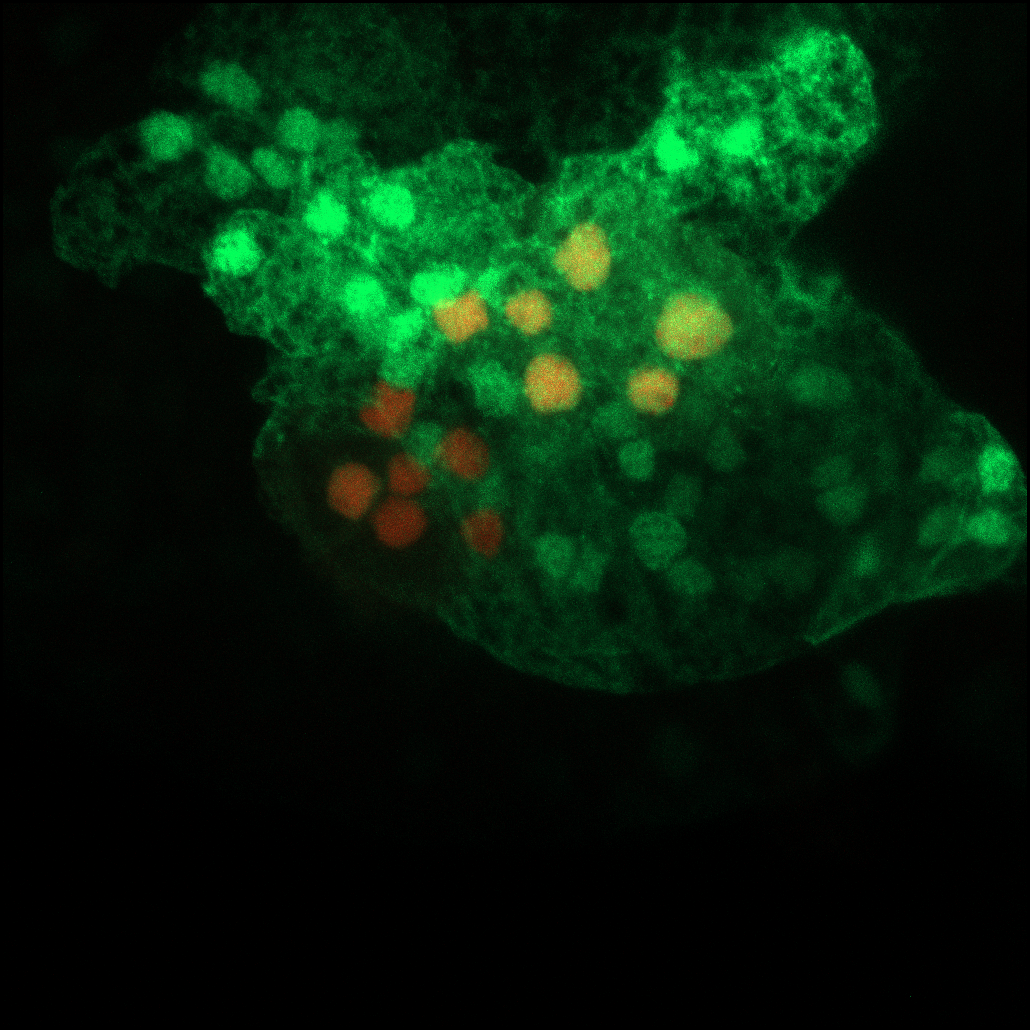

Supplement: Supplementary file 15 — Source data Fig. 8 [file 44318_2025_613_MOESM15_ESM.zip › Figure 8/Figure 8K/-3691_-1.tif]

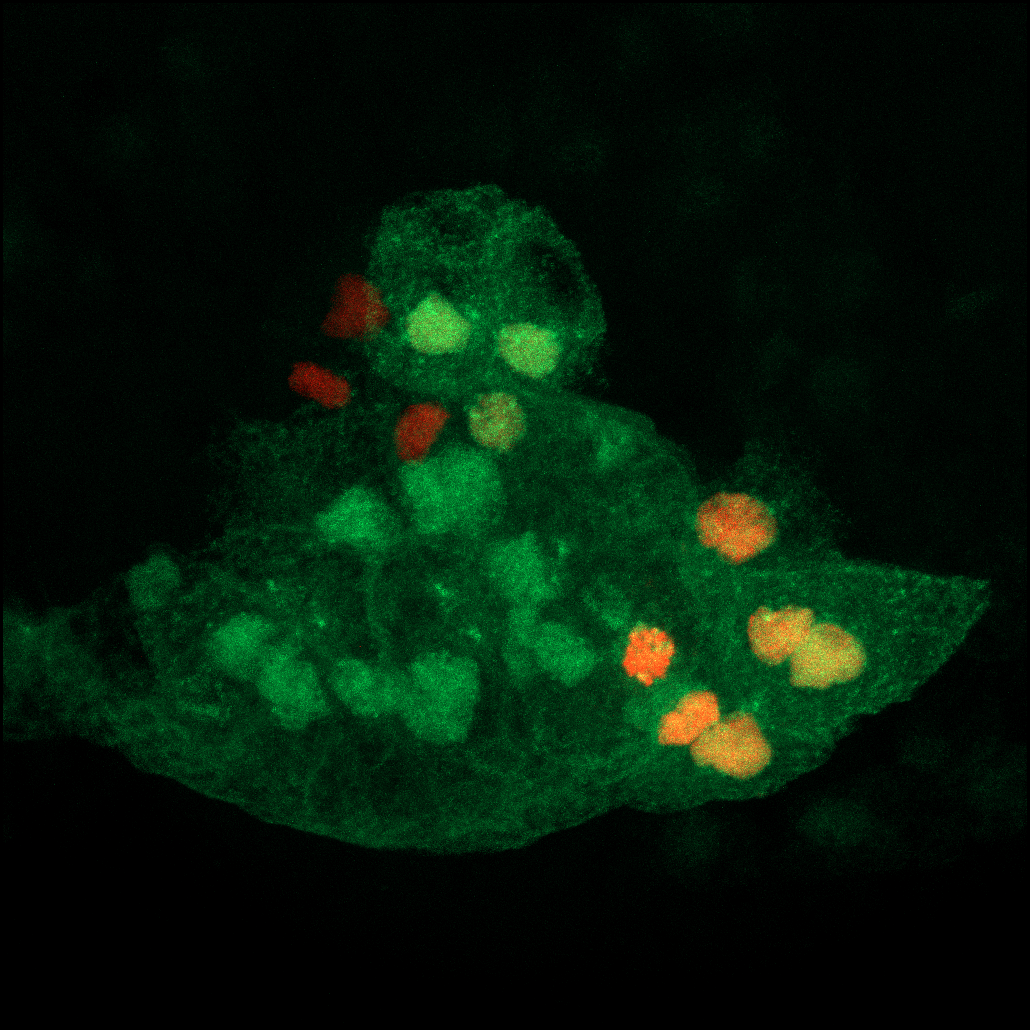

Supplement: Supplementary file 15 — Source data Fig. 8 [file 44318_2025_613_MOESM15_ESM.zip › Figure 8/Figure 8K/-2786_-2062.tif]

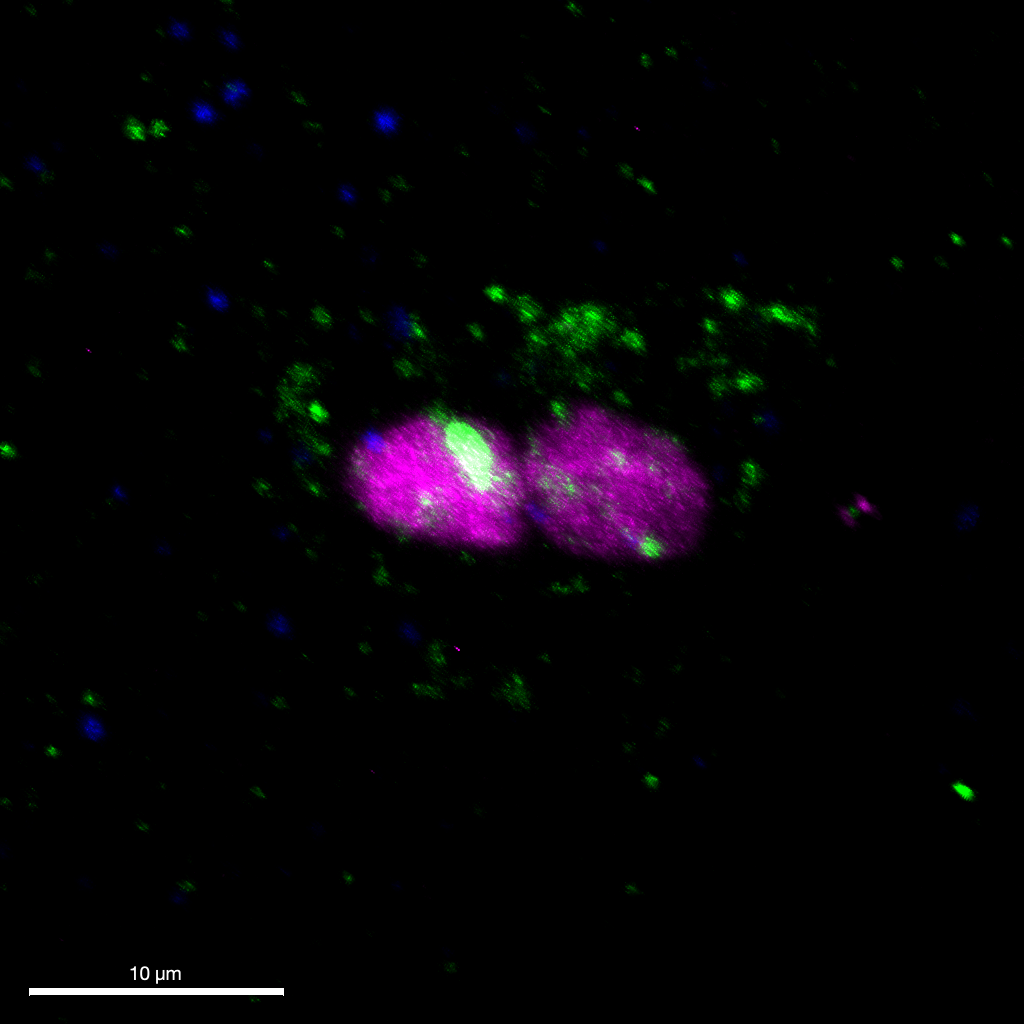

Supplement: Supplementary file 15 — Source data Fig. 8 [file 44318_2025_613_MOESM15_ESM.zip › Figure 8/8E/20231004 EP33III_Tyr_11H_[ii13_EP33III_gataDep_Tyr_11H_14-1024_Image_14]_2024-03-22T06-09-37.978.tif]

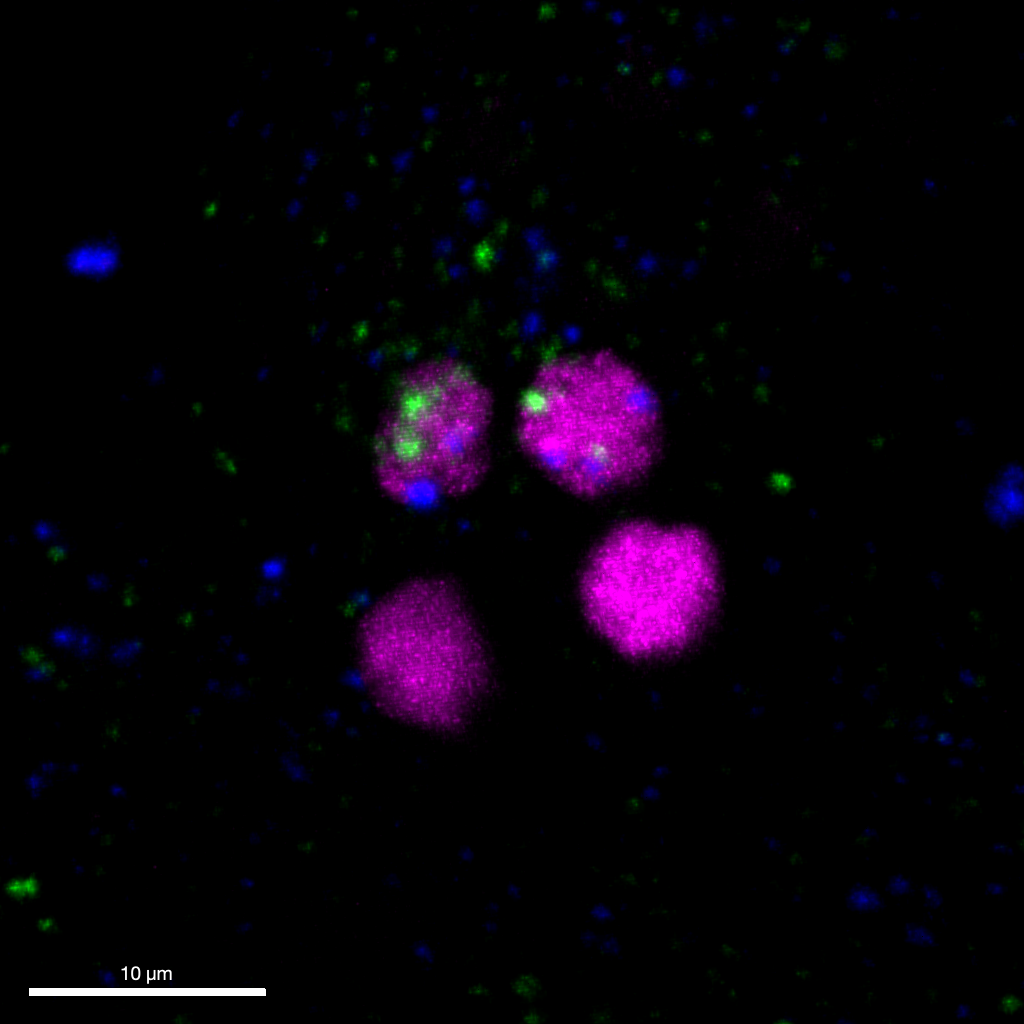

Supplement: Supplementary file 15 — Source data Fig. 8 [file 44318_2025_613_MOESM15_ESM.zip › Figure 8/8E/20231005 EP33III_Tyr_10H_[ii6_EP33III_gataDep_Tyr_10H_6_Image_7]_2024-03-22T06-06-34.526.tif]

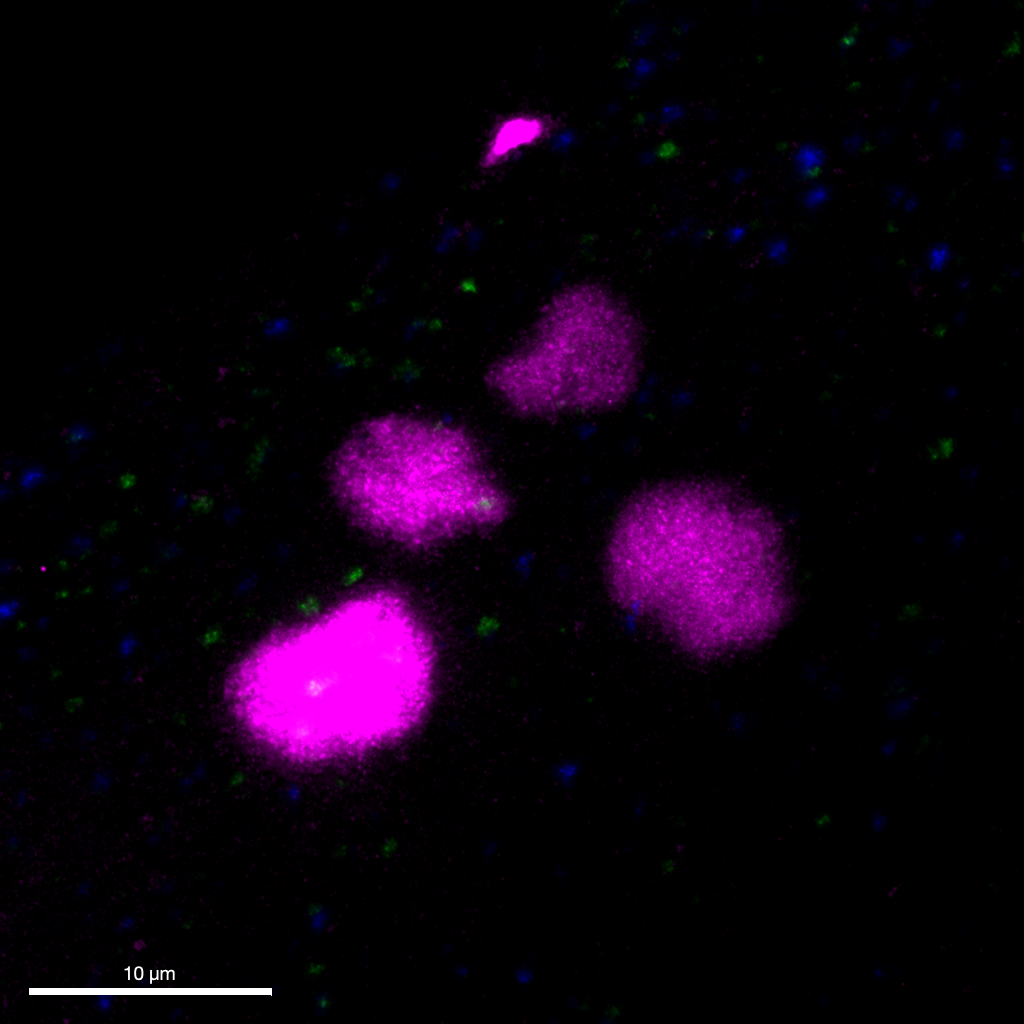

Supplement: Supplementary file 15 — Source data Fig. 8 [file 44318_2025_613_MOESM15_ESM.zip › Figure 8/8E/20231006 EP33III_Tyr_9H_[ii9_EP33III_gataDep_Tyr_9H_10-0.22_Image_10]_2024-03-22T06-04-21.806.tif]

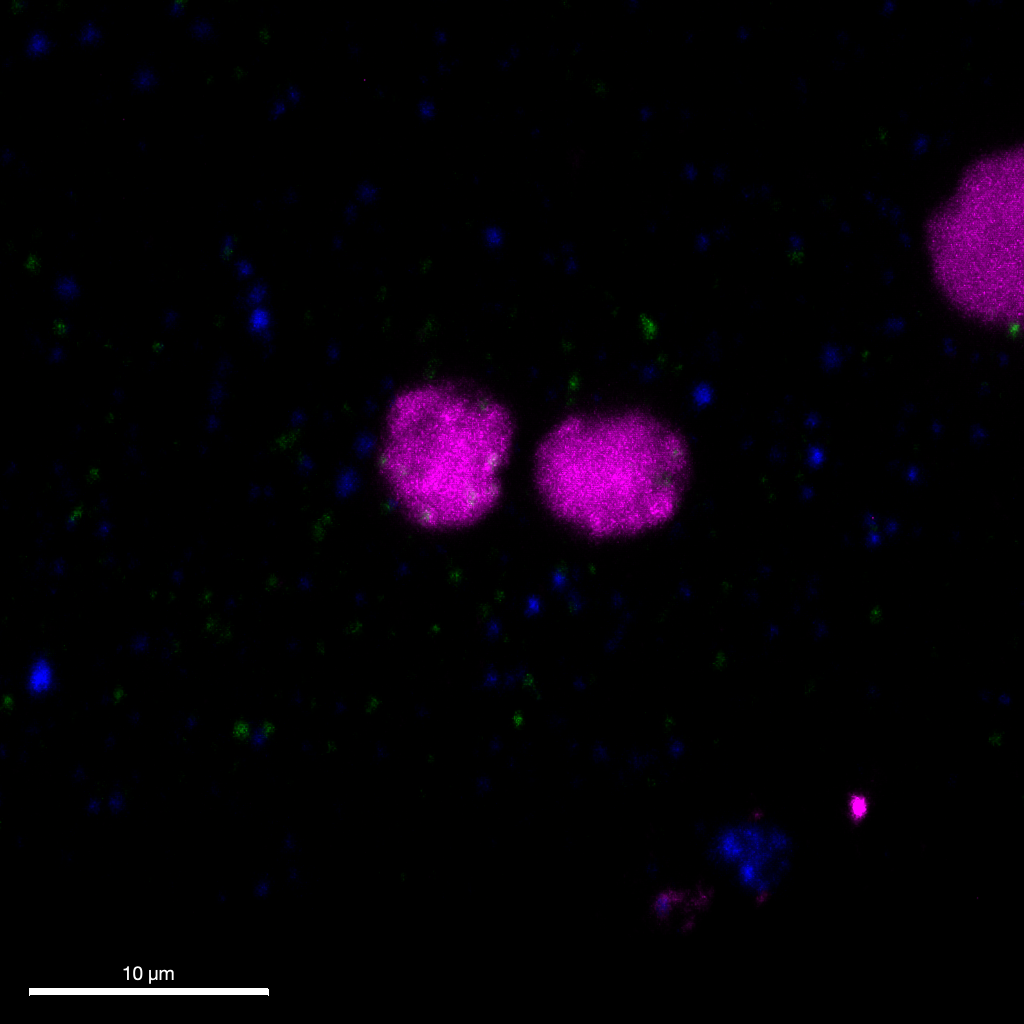

Supplement: Supplementary file 15 — Source data Fig. 8 [file 44318_2025_613_MOESM15_ESM.zip › Figure 8/8E/20231004 EP33III_Gata_11H_[ii7_EP33III_gataDep_Gata_11H_7-1024_Image_8]_2024-03-22T05-59-10.676.tif]

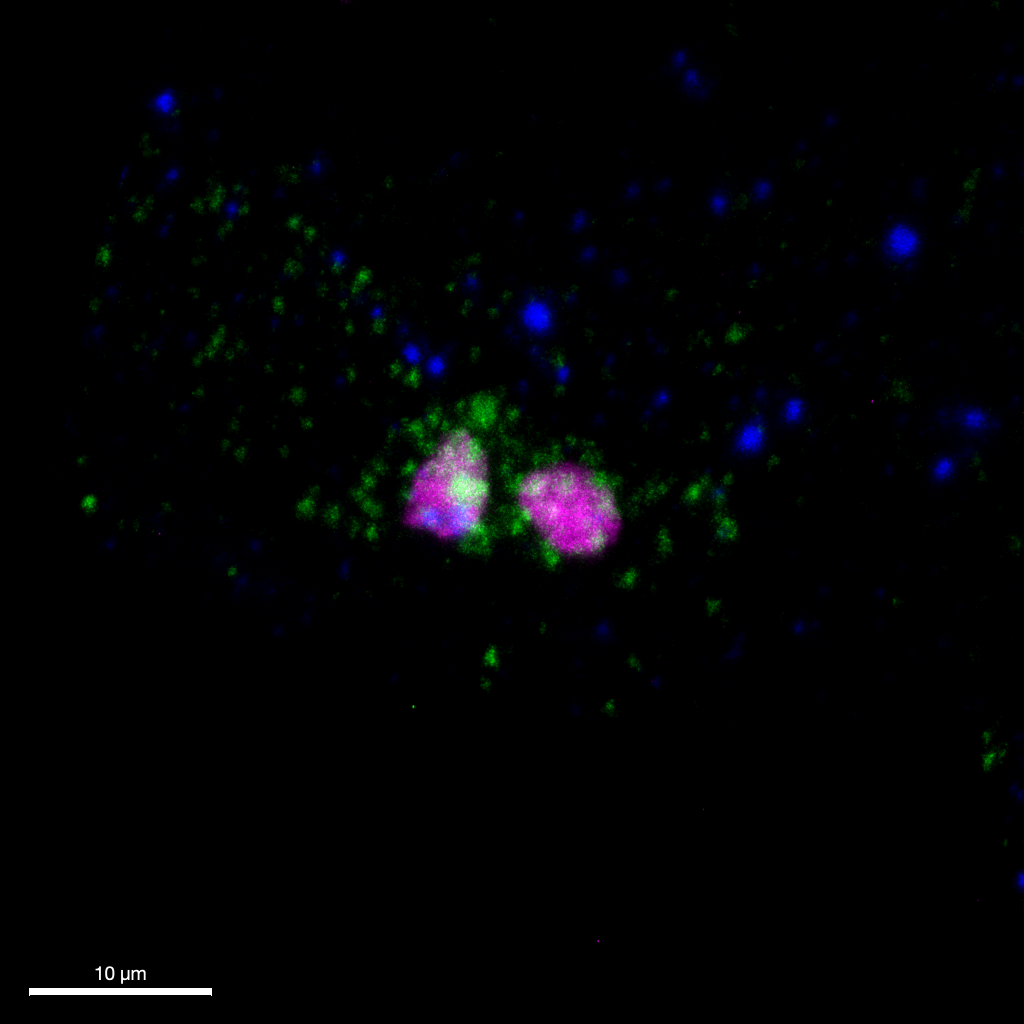

Supplement: Supplementary file 15 — Source data Fig. 8 [file 44318_2025_613_MOESM15_ESM.zip › Figure 8/8E/20231005 EP33III_Tyr_12H_[ii4_EP33III_gataDep_Tyr_12H_5-1024_Image_5]_2024-03-22T09-19-39.672.tif]

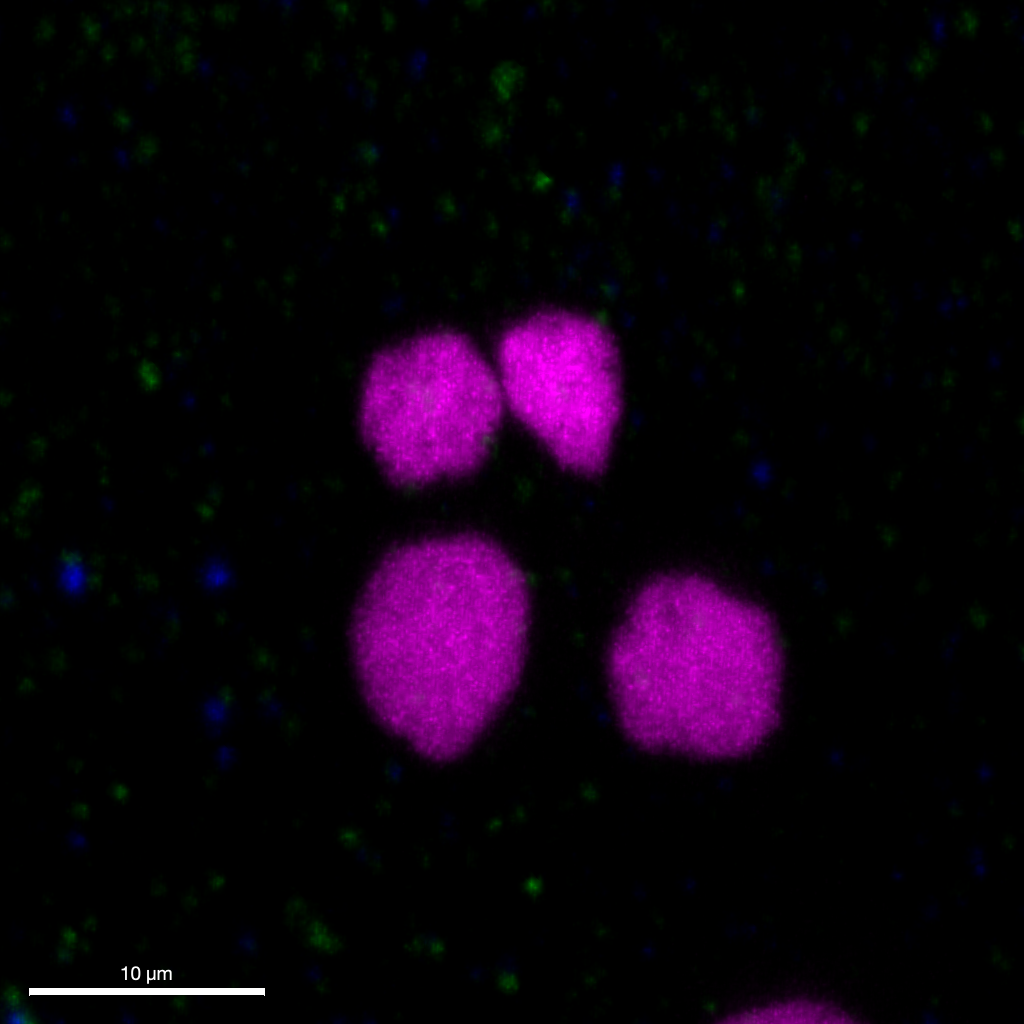

Supplement: Supplementary file 15 — Source data Fig. 8 [file 44318_2025_613_MOESM15_ESM.zip › Figure 8/8E/20231006 EP33III_Gata_9H_[ii2_EP33III_gataDep_Gata_9H_3_Image_3]_2024-03-22T05-55-16.930.tif]

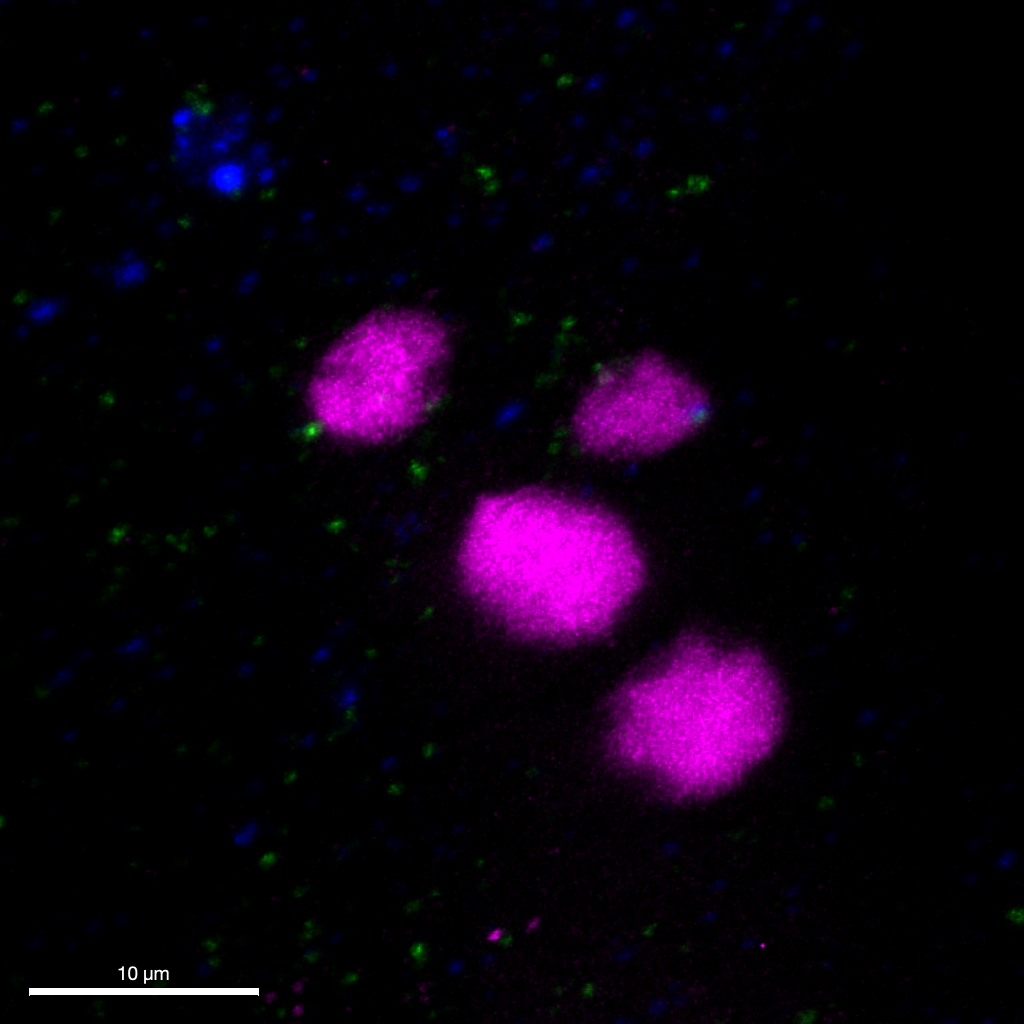

Supplement: Supplementary file 15 — Source data Fig. 8 [file 44318_2025_613_MOESM15_ESM.zip › Figure 8/8E/20231005 EP33III_Gata_10H_[ii3_EP33III_gataDep_Gata_10H_4_Image_4]_2024-03-22T05-57-35.762.tif]

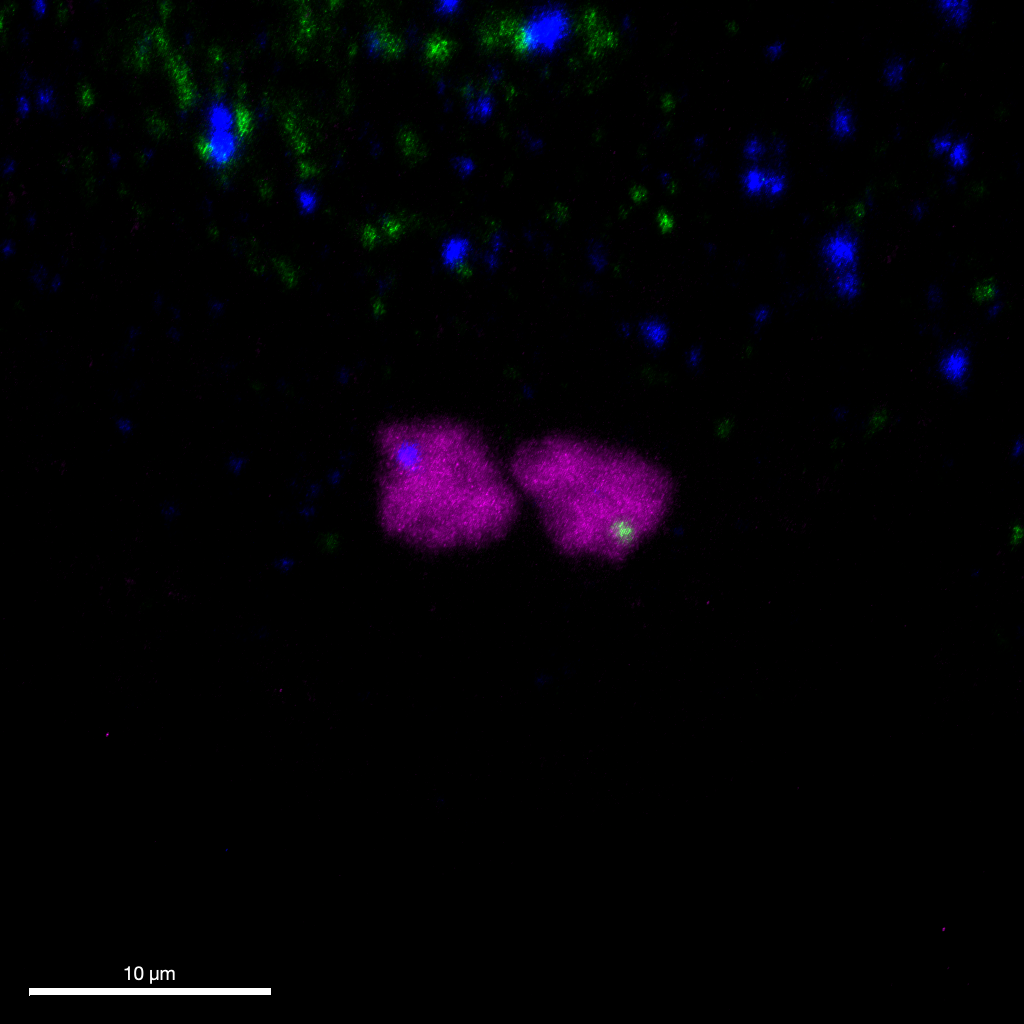

Supplement: Supplementary file 15 — Source data Fig. 8 [file 44318_2025_613_MOESM15_ESM.zip › Figure 8/8E/20231005 EP33III_Gata_12H_[ii2_EP33III_gataDep_Gata_12H_3-1024_Image_3]_2024-03-22T06-02-41.336.tif]

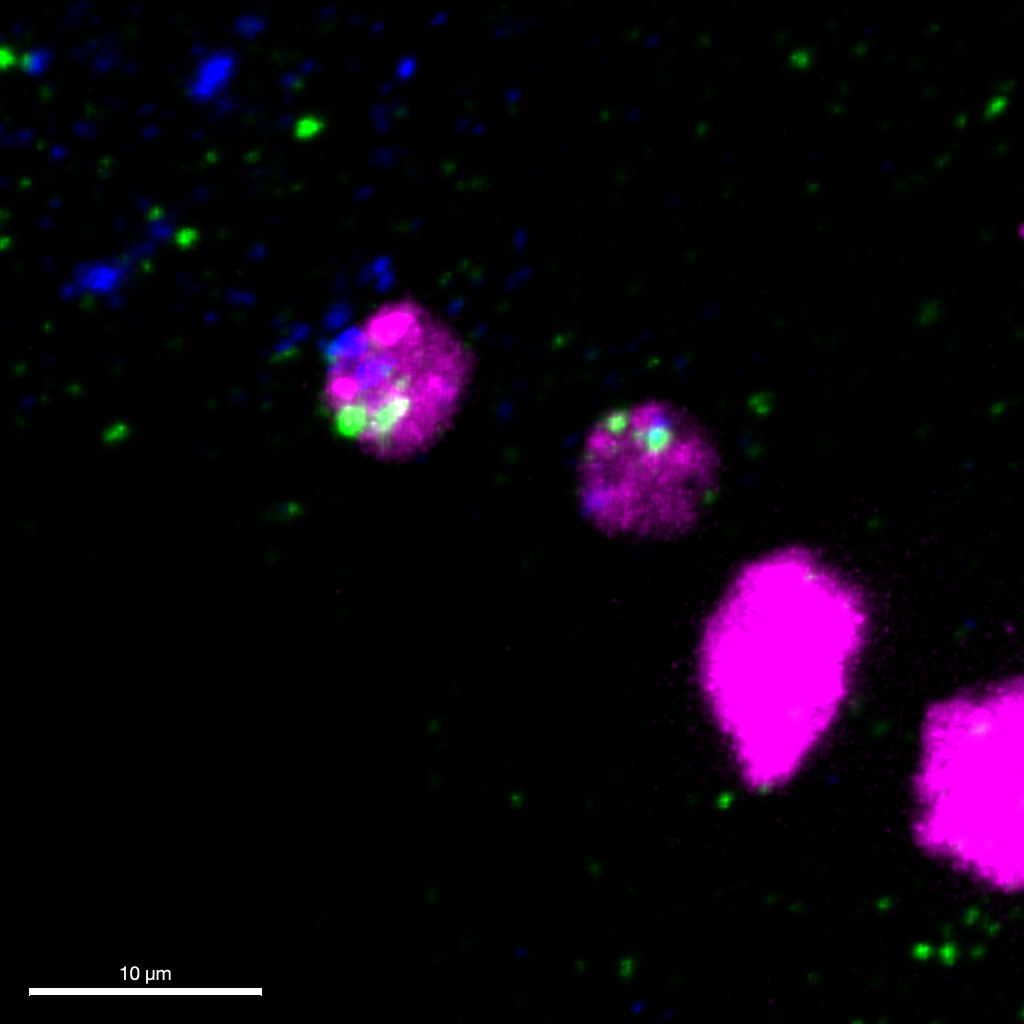

Supplement: Supplementary file 16 — Source data Fig. 9 [file 44318_2025_613_MOESM16_ESM.zip › Figure 9/9B/20230718 EP32_HA_10H_Re_[ii5_EP32_HA_10H_16_Image_6]_2024-03-22T06-57-20.088.tif]

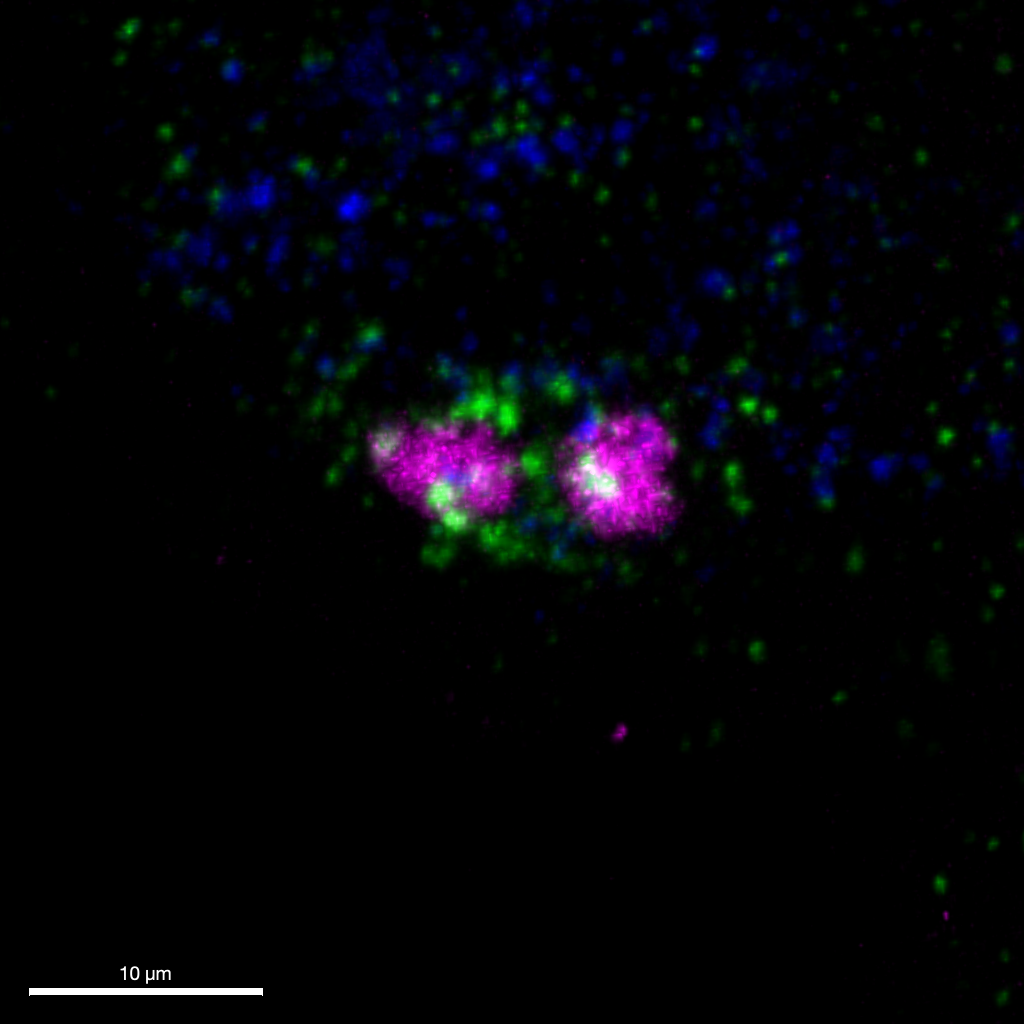

Supplement: Supplementary file 16 — Source data Fig. 9 [file 44318_2025_613_MOESM16_ESM.zip › Figure 9/9B/20230713 EP32_HA_12H_[ii9_EP32_HA_12H_10_Image_10]_2024-03-22T07-02-30.499.tif]

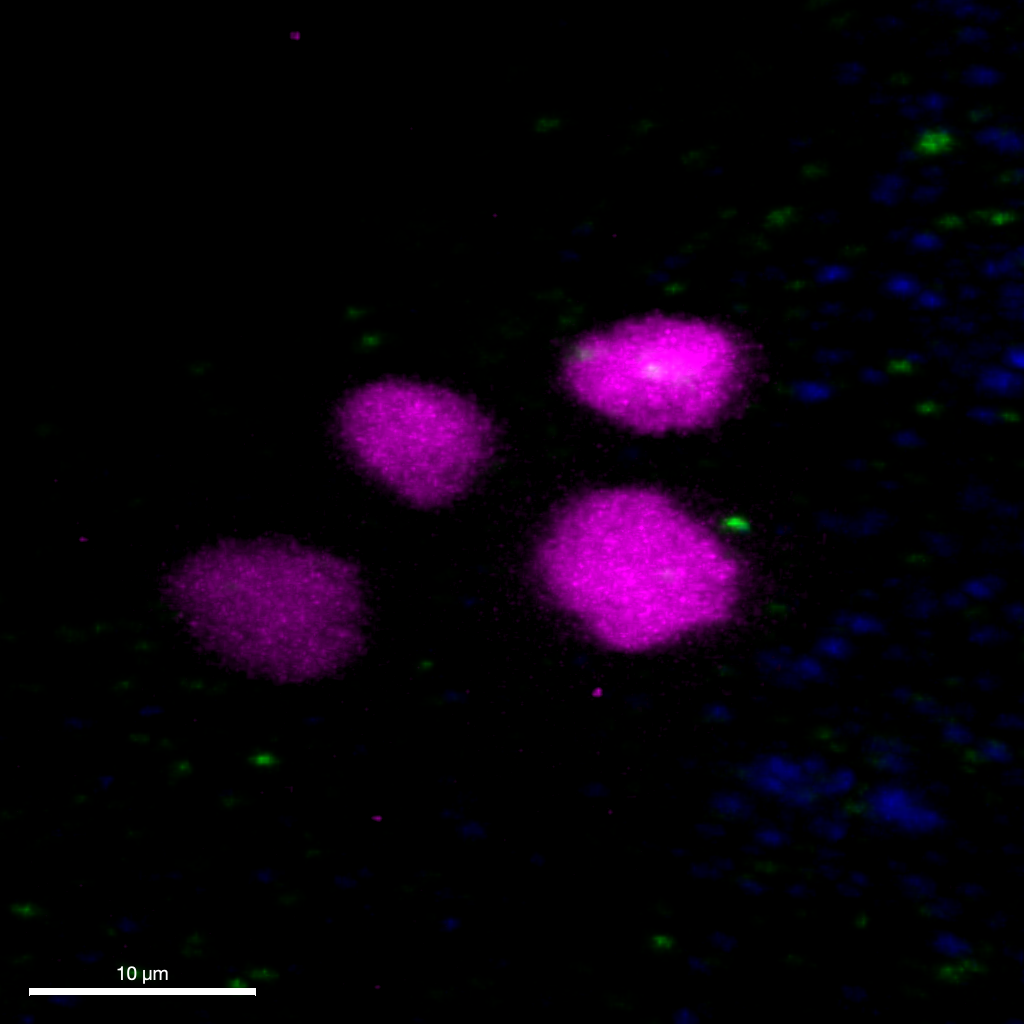

Supplement: Supplementary file 16 — Source data Fig. 9 [file 44318_2025_613_MOESM16_ESM.zip › Figure 9/9B/20230713 EP32_HA_8H_[ii5_EP32_HA_8H_12_Image_6]_2024-03-22T06-51-56.267.tif]

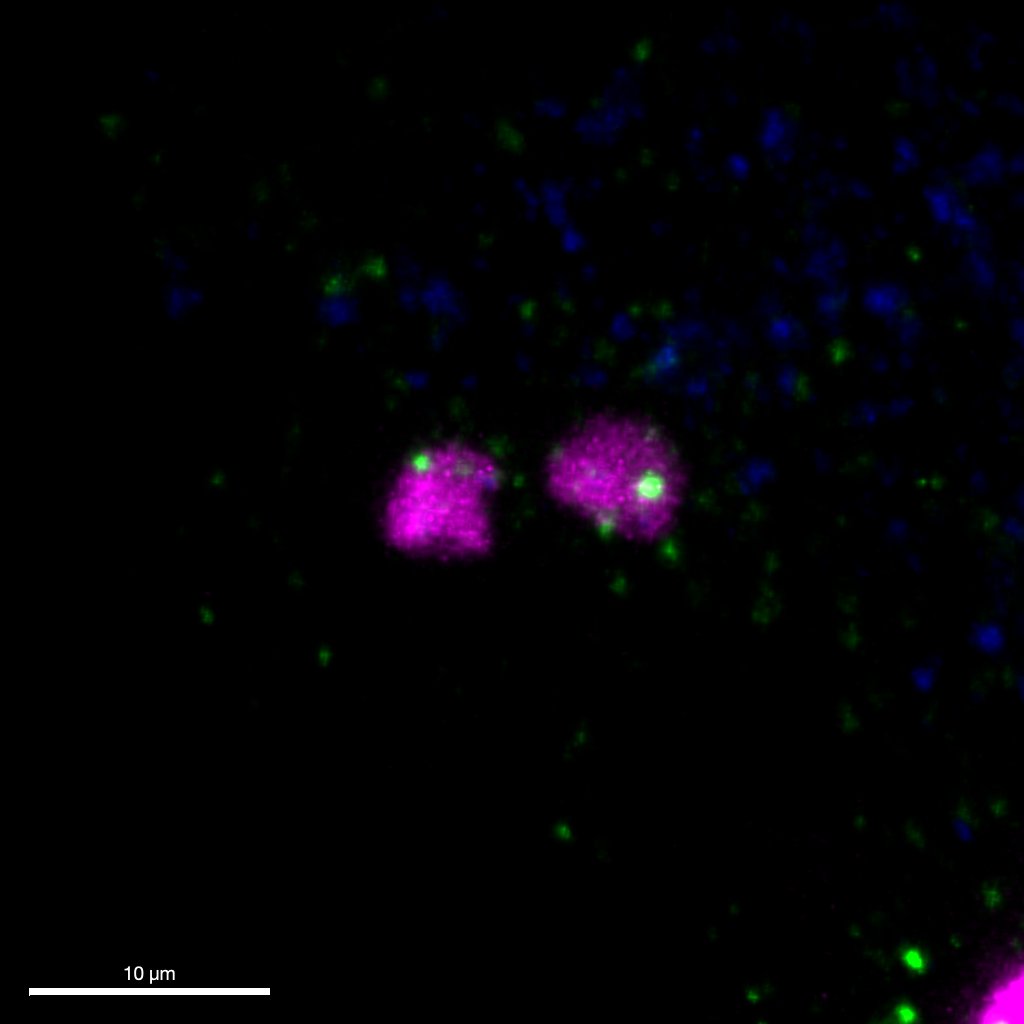

Supplement: Supplementary file 16 — Source data Fig. 9 [file 44318_2025_613_MOESM16_ESM.zip › Figure 9/9B/20230713 EP32_Cdkn1b_12H_[ii10_EP32_Cdkn1b_12H_10_Image_11]_2024-03-22T07-00-00.617.tif]

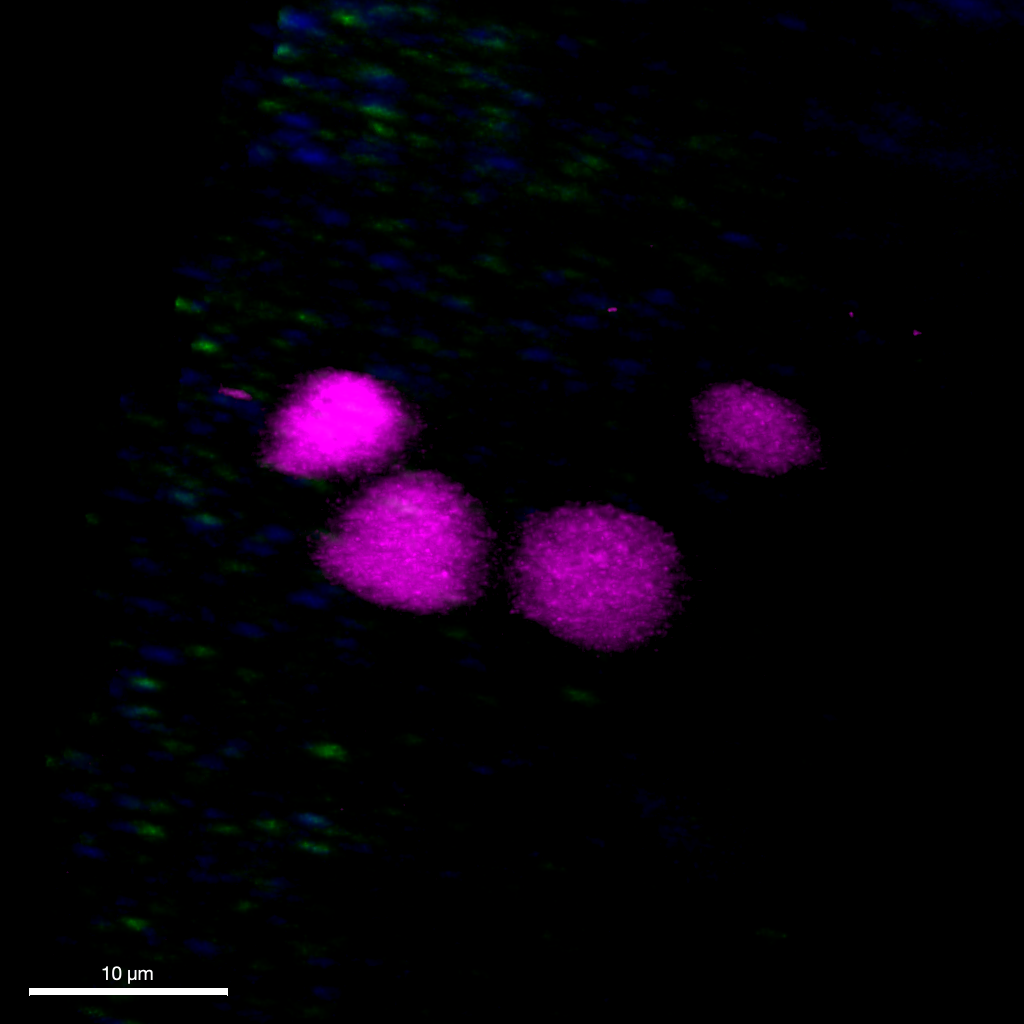

Supplement: Supplementary file 16 — Source data Fig. 9 [file 44318_2025_613_MOESM16_ESM.zip › Figure 9/9B/20230713 EP32_Cdkn1b_8H_[ii1_EP32_Cdkn1b_8H_2_Image_2]_2024-03-22T06-50-27.421.tif]

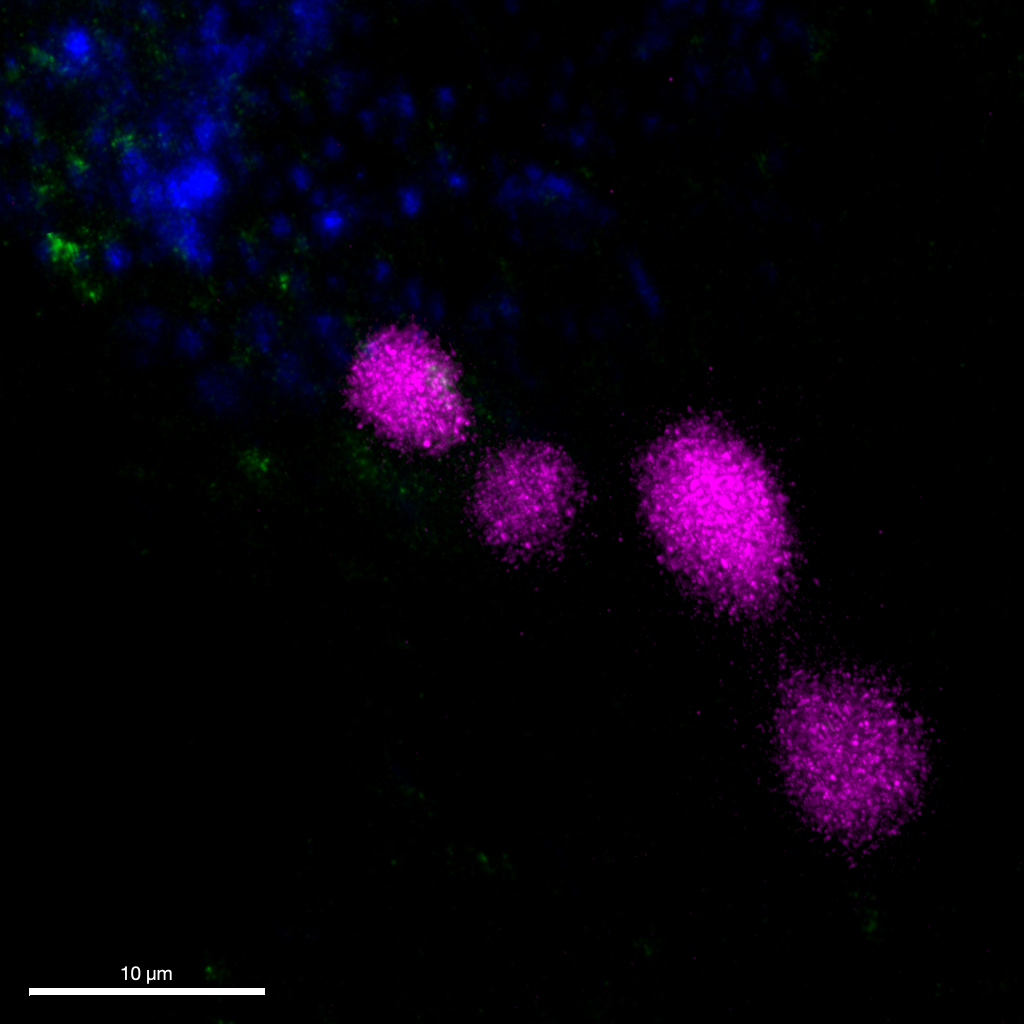

Supplement: Supplementary file 16 — Source data Fig. 9 [file 44318_2025_613_MOESM16_ESM.zip › Figure 9/9B/20230718 EP32_Cdkn1b_10H_Re_[ii14_EP32_Cdkn1b_10H_32_Image_15]_2024-03-22T06-55-18.581.tif]
