# Supplementary material for: Cell cycle-driven transcriptome maturation confers multilineage competence to cardiopharyngeal progenitors
Source: EMBO J. 2025 Nov 3;44(24):7649–76. doi: 10.1038/s44318-025-00613-y (PMC12705688; doi:10.1038/s44318-025-00613-y)
Supplement: Supplementary file 15 — Source data Fig. 8 [file 44318_2025_613_MOESM15_ESM.zip › Figure 8/Figure 8O/Readme.docx]

Confocal z-stacks were processed with maximum projection and adjusted for brightness before export as .tif files. Adobe Illustrator was used to rotate, crop, and add a dashed midline and arrows to the original .tif file.
